# Effect of Medications for Type 2 Diabetes on Cardiovascular Events and Mortality: A Comprehensive Pairwise and Network Meta‐Analysis

**DOI:** 10.1111/dom.71128

**Published:** 2026-07-30

**Authors:** Giovanni Antonio Silverii, Valerio Velardi, Christian Marinelli, Antonino Lax, Matteo Monami, Edoardo Mannucci

**Affiliations:** ^1^ Diabetology Unit Careggi Hospital Florence Italy; ^2^ University of Florence Florence Italy; ^3^ Department of Medical Surgical and Health Sciences University of Trieste Trieste Italy

**Keywords:** antidiabetic drug, cardiovascular disease, meta‐analysis, network meta‐analysis, type 2 diabetes

## Abstract

**Aim:**

To assess the effect of all the approved classes of medications for type 2 diabetes on Major Adverse Cardiovascular events (MACE) and all‐cause mortality.

**Methods:**

We performed a pairwise and network meta‐analysis, including randomised controlled trials with a duration of at least 40 weeks, comparing medications versus placebo or an active comparator, in which MACE and deaths were adjudicated.

**Results:**

We included 93 studies. In pairwise meta‐analyses, versus all comparators, GLP‐1 receptor agonists (GLP1RA), SGLT‐2 inhibitors (SGLT2i), metformin and pioglitazone were associated with a significant reduction of MACE and sulfonylureas with increased MACE. Furthermore, tirzepatide, SGLT2i and GLP1RA were associated with a significantly reduced all‐cause mortality. In the principal (frequentist) network meta‐analysis, metformin (OR 0.69, 95% CI 0.53–0.89), SGLT2i (0.82, 0.75–0.90), GLP1RA (0.87, 0.83–0.92) and tirzepatide (0.82, 0.72–0.93) were associated with a significant reduction of MACE versus placebo; no effect was observed for insulin, DPP4 inhibitors (DPP4i) or sulfonylureas. Tirzepatide (0.72, 0.64–0.82), SGLT2i (0.85, 0.80–0.91) and GLP1RA (0.85, 0.80–0.91) were associated with a significantly reduced mortality versus placebo; no effect was detected for other drugs. The sensitivity (Bayesian) analysis did not confirm the significance of results for pioglitazone on MACE. The certainty of evidence was moderate‐to‐high for GLP1RA, SGLT2i, Tirzepatide, DPP4i; low for metformin; low‐to‐moderate for other drugs.

**Conclusion:**

SGLT2i, GLP1RA, tirzepatide and (with lower quality of evidence) pioglitazone and metformin are associated with a reduced incidence of cardiovascular events; tirzepatide, SGLT2i and GLP1RA are also associated with a reduced all‐cause mortality.

## 1 Introduction

The treatment of type 2 diabetes is aimed at reducing the risk of acute and chronic complications of hyperglycemia. Among those, cardiovascular disease is a major cause of death and disability. Beyond the possible benefits of glycemic control on cardiovascular risk [1], the choice of glucose‐lowering medications is a major determinant of cardiovascular outcomes [2]. In fact, randomised controlled trials, often originally designed for the assessment of the long‐term safety of specific medications, have shown that some classes of drugs are able to reduce cardiovascular morbidity and mortality in patients with type 2 diabetes. In particular, metformin [3], Sodium‐Glucose Transporter 2 inhibitors (SGLT2i) and Glucagon‐like peptide 1 Receptor agonists (GLP1RA) have been reported to reduce the incidence of major adverse cardiovascular events (MACE) in people with type 2 diabetes mellitus [4, 5, 6]. The effect of pioglitazone, which could reduce MACE but increases the risk of hospitalisation for heart failure, is still debated [7, 8], whereas Dipeptidyl Peptidase‐4 inhibitors (DPP4i) and insulin do not appear to have any relevant effect on cardiovascular risk [9]. Conversely, sulfonylureas may increase the risk for MACE [10].

The evidence base for assessing the effects of drugs for type 2 diabetes on cardiovascular outcomes evolves with the progressive accumulation of new trials. In recent years, the development of new drugs (most notably, the dual GLP1/GIP receptor agonist tirzepatide) and the publication of results of new trials on other already available drugs have changed the general landscape. Notably, although most trials compare an active drug with a placebo, some studies are head‐to‐head comparisons between active drugs. In particular, the largest trial with adjudicated cardiovascular outcomes performed with tirzepatide is a comparison versus dulaglutide, which had already demonstrated cardiovascular benefits [11]. In this context, the use of traditional meta‐analyses can be misleading, underestimating potential benefits if trials versus active comparators are included and reducing the evidence base if they are excluded. A Network Meta‐Analysis (NMA), allowing multiple comparisons with the inclusion of both placebo‐ and active comparator‐controlled trials, can overcome such issues, providing a comprehensive summary of current evidence [12]. However, results of NMA need to be considered with some caution, because differences in characteristics of enrolled samples and/or trial procedures across different studies can produce distortions in indirect comparisons. A living systematic review with NMA, including all classes of drugs for type 2 diabetes and assessing their effects on cardiovascular events and all‐cause mortality [13], does not yet include the recently published cardiovascular outcome trial with tirzepatide [11], not allowing a reliable estimate of the effects of this agent on cardiovascular morbidity and mortality. In addition, that living systematic review is not specifically aimed at assessing cardiovascular outcomes, considering a wider range of endpoints; consequently, it includes shorter‐term trials (useful for metabolic outcomes, but potentially attenuating differences on cardiovascular endpoints) and drugs specifically aimed at other conditions associated with diabetes, such as finerenone. The present NMA is specifically designed for summarising all current evidence on the effect of drugs for type 2 diabetes on MACE and all‐cause mortality, also providing data for tirzepatide.

## 2 Methods

This NMA was reported following the criteria of Preferred Reporting Items for Systematic Reviews and Meta‐Analyses (PRISMA) statement [14]. The protocol of the present meta‐analysis was published on the PROSPERO website (https://www.crd.york.ac.uk/prospero/#recordDetails; registration number: CRD420261290664).

The principal endpoint was MACE, a composite of Cardiovascular Death, nonfatal myocardial infarction and nonfatal stroke. The secondary endpoints are all‐cause mortality, nonfatal stroke and nonfatal myocardial infarction.

### 2.1 Search Strategy

A Medline, Embase and Cochrane central search was performed up to January 31, 2026; detailed information on the search strategy and keywords used is reported in Table S1. Animal studies were excluded, whereas no language or date restriction was imposed.

### 2.2 Inclusion Criteria

We included all randomised controlled trials with a duration of 40 weeks or longer performed in adults (≥ 18 years) with established type 2 diabetes mellitus, in which any glucose‐lowering drug approved by the European Medicines Agency (EMA) as a treatment for type 2 diabetes mellitus was compared with placebo or any other EMA‐approved glucose‐lowering drug and cardiovascular events and deaths were independently adjudicated by an external committee. Studies were included independent of blinding. The choice of a 40‐week duration as an inclusion criterion is somewhat arbitrary (like any other threshold in that respect) and it allows for a duration of treatment of at least 6 months after drug titration, for those agents needing progressive up‐titration at the beginning of treatment. This threshold was chosen as a reasonable compromise between the need to exclude shorter‐term trials, which could blunt differences between drug classes for cardiovascular endpoints and the need to retrieve a sufficiently large number of trials, with relevant sample sizes to increase the reliability of comparisons.

We excluded trials in which the control arm consisted of two or more different drugs (either as predefined combination therapies or as two different treatments prescribed separately to each or different subjects) and no data on subgroups were provided, as well as trials in which intervention and comparator arms consisted of different doses of the same drug.

### 2.3 Data Extraction

Information on the baseline characteristics of the enrolled samples and the incidence of MACE and all‐cause mortality was extracted from the principal publication when available; when needed, secondary publications and the ClinicalTrials.gov registry were used to retrieve missing information, in the hierarchical order reported above. Four authors (A.L., C.M., G.A.S., V.V.) performed data extraction independently and conflicts were resolved by a fifth investigator (E.M.).

The risk of bias was assessed using the Cochrane recommended ROB2.0 tool to determine the risk of bias in Randomised Controlled Trials and with CINeMA software to assess bias in meta‐analysis. The risk of bias was described and evaluated in five specific domains: randomisation process, deviations from intended interventions, missing outcome data, measurement of the outcome, selection of the reported result; a further domain encompassing all the previous was specified as ‘overall bias’. The results of these domains were graded as ‘low’ risk of bias, ‘high’ risk of bias or ‘uncertain’ risk of bias.

### 2.4 Statistical Analysis

Mantel‐Haenzel Odds Ratio for categorical variables were calculated, using random effect models. We performed an NMA for all the outcomes listed above, to verify differences across individual glucose‐lowering strategies concerning their effects on primary and secondary endpoints. These analyses allow indirect comparisons when direct trials are unavailable, using differences from common comparators and then combining direct and indirect comparisons for a final estimate of effects. The reference category was placebo. The NMA was performed within a pairwise modelling framework using the Meta Insight software. H values were calculated to evaluate consistency between direct and indirect evidence; *p* > 0.05 for inconsistency indicates non‐significant inconsistency of treatment effects. Publication bias was assessed through the visual analysis of funnel plots; when publication bias was not ruled out through funnel plot, the Egger's test was performed, using Comprehensive Meta‐analysis. All other analyses were performed using Review Manager (RevMan), Version 5.3 (Copenhagen: The Nordic Cochrane Centre, The Cochrane Collaboration, 2014).

## 3 Results

### 3.1 Retrieved Trials

The search of Medline, Embase and Cochrane databases allowed the identification of 13 503 items; after excluding 13 164 studies by reading the title and the abstract, 247 further trials were excluded after reviewing the full text (See Figure S1, Table S2). We therefore retrieved 93 trials fulfilling all inclusion criteria (two of which were included in the same paper) [3]. The principal characteristics of trials included in the analysis are reported in Table S3.

The quality of studies was generally satisfactory (Figures S2 and S3), with five studies in which potential issues with randomisation could not be ruled out and seven studies in which deviation from the intended intervention could not be excluded.

### 3.2 Principal Outcome: MACE

#### 3.2.1 Traditional Meta‐Analyses (By Class)

The visual analysis of funnel plots ruled out publication bias for trials performed with all drug classes, except for sulfonylureas (Figure S4). This finding was confirmed by the Egger's test, which showed a significant publication bias only for sulfonylureas (*p* = 0.02, Figure S4). In comparisons versus all comparators, GLP1RA, SGLT2i, metformin and pioglitazone, but not tirzepatide, were associated with a significant reduction of MACE (Figures S5–S9). For GLP1RA, the effect was significant for trials versus placebo, but not versus active comparators (Figure S5). For SGLT2i, the result was largely driven by trials vs. placebo (Figure S6) and for tirzepatide by trials versus GLP1RA (Figure S9). DPP4i and insulin were neutral in respect to cardiovascular risk (Figures S10 and S11), whereas sulfonylureas were associated with a significant increase in MACE (Figure S12).

#### 3.2.2 Network Meta‐Analysis

**FIGURE 1:**
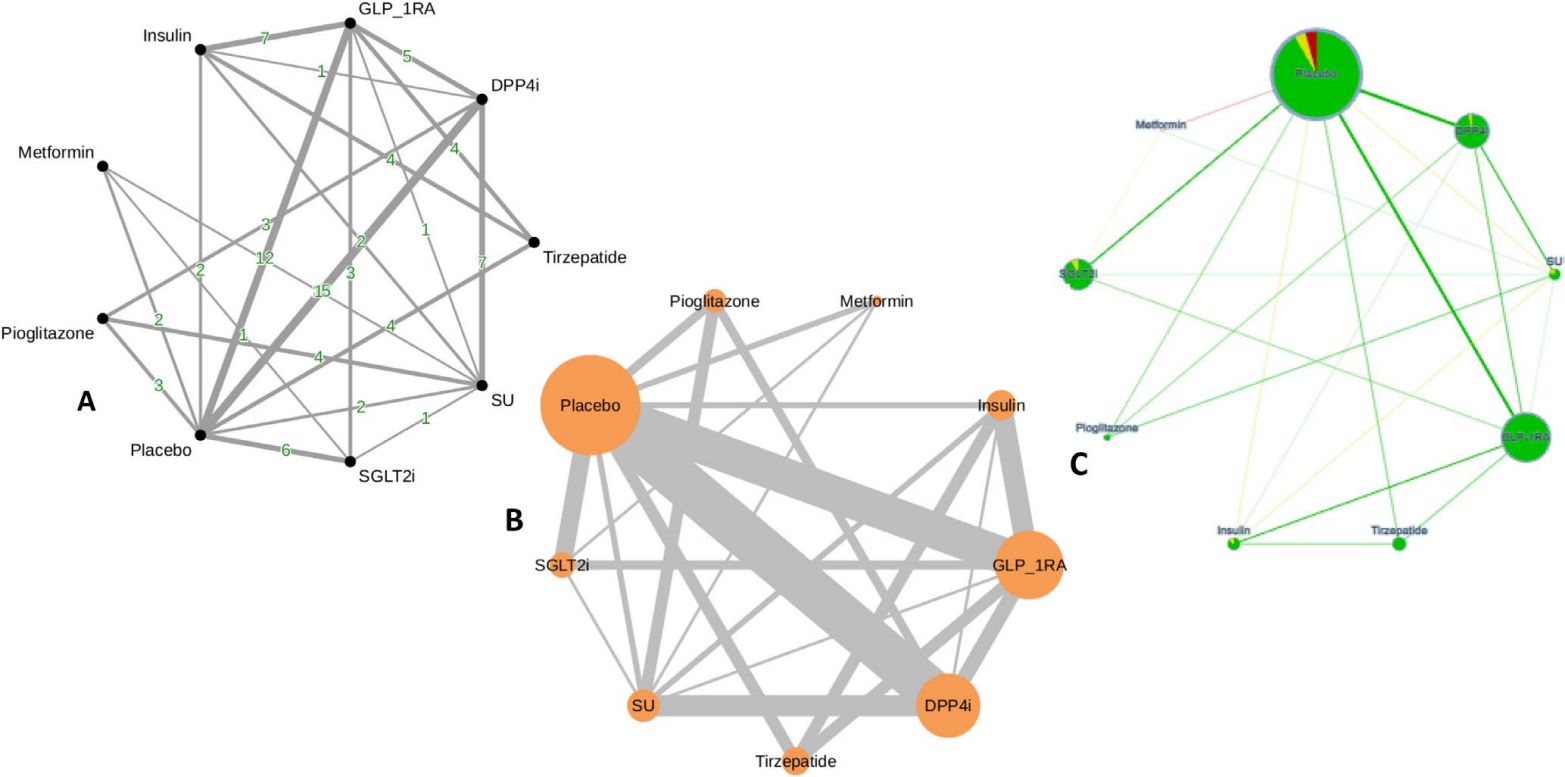
Network plot for Major Adverse Cardiovascular events. (A) The numbers on the nodes are the number of trials for each comparison. (B and C) Node size is proportional to the number of included patients, width of lines between nodes is proportional to the number of included trials. (C) The proportion of green and Yellow in the nodes reflects the within‐trial risk of bias.

**FIGURE 2:**
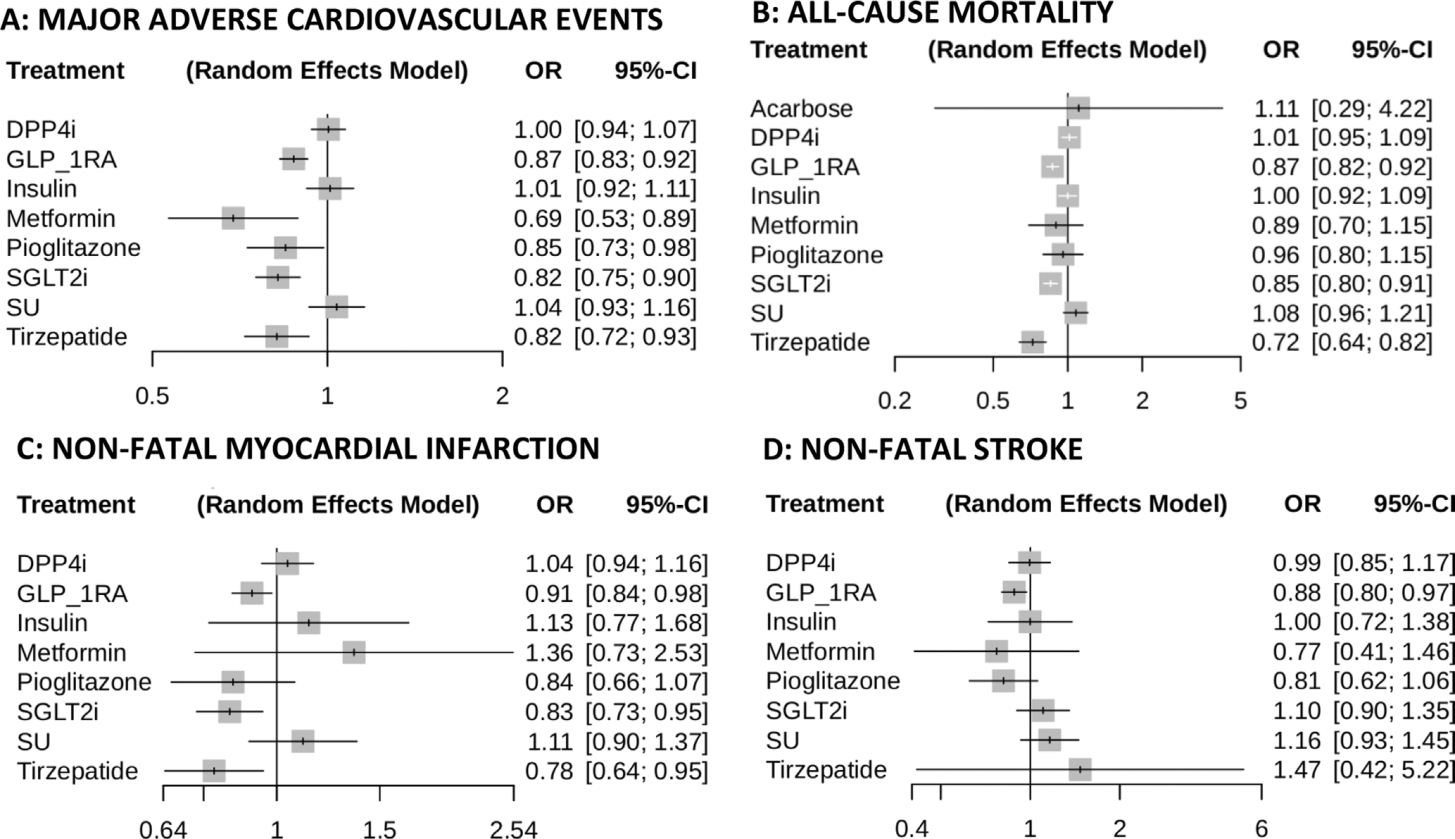
frequentist Meta‐analysis, forest plots: Difference in the incidence of Major Adverse Cardiovascular events (A), All‐cause mortality (B), Non‐Fatal Myocardial infarction (C), Non‐Fatal Stroke (D) between Diabetes drugs classes and placebo; CI, confidence interval; OR, odds ratio.

The analysis included 85 studies (82 two‐arm studies and 3 multi‐arm studies, of which 4 had zero events and 16 had at least one zero‐event arm) assessed 9 interventions (8 drug classes and placebo), overall including 21 174 MACE in 247 193 patients. Out of 36 possible pairwise comparisons, 22 comparisons with direct data were retrieved (Figure 1). GLP1RA were investigated in 32 studies, DPP4i in 28, sulfonylureas in 14, SGLT2i and insulin in 13 studies each, tirzepatide in 12, pioglitazone in 10, whereas only 4 studies used metformin; most of the trials (46) were versus placebo (Figure 1). In the principal (frequentist) analysis, metformin (OR 0.68, 95% CI 0.51–0.92), SGLT2i (OR 0.82, 0.75–0.90), GLP1RA (OR 0.88, 0.83–0.93), tirzepatide (OR 0.83, 0.72–0.96) and pioglitazone (OR 0.85, 0.73–0.98) were associated with a significant reduction of MACE in respect to placebo, whereas no effect was observed for insulin, DPP4i, or sulfonylureas (Figure 2A).

In direct comparisons, metformin was superior to sulfonylureas; SGLT2i and pioglitazone were superior to placebo, whereas GLP1RA was superior to placebo, DPP4i and insulin (Table 1). Conversely, tirzepatide was not statistically different from any comparator, including placebo. In indirect (network) comparisons, metformin, SGLT2i, GLP1RA and tirzepatide were all superior to placebo, DPP4i, insulin and sulfonylureas, whereas pioglitazone was superior to insulin and sulfonylureas only and DPP4i to sulfonylureas only (Table 1). In the sensitivity (Bayesian) analysis, metformin, GLP1RA and SGLT2i and tirzepatide remained associated with a significant reduction of MACE versus placebo, whereas results with pioglitazone were not statistically significant (Figure S13). A significant inconsistency was observed only for the comparison of metformin vs. SGLT2i (with metformin superior in direct comparison but not in indirect comparison and NMA, Figure S14).

**TABLE 1:** Frequentist treatment effect: Summary forest plots direct comparisons (in black) and indirect comparisons (in grey) between diabetes drug classes in the incidence of Major Adverse Cardiovascular Events (dir. = direct).

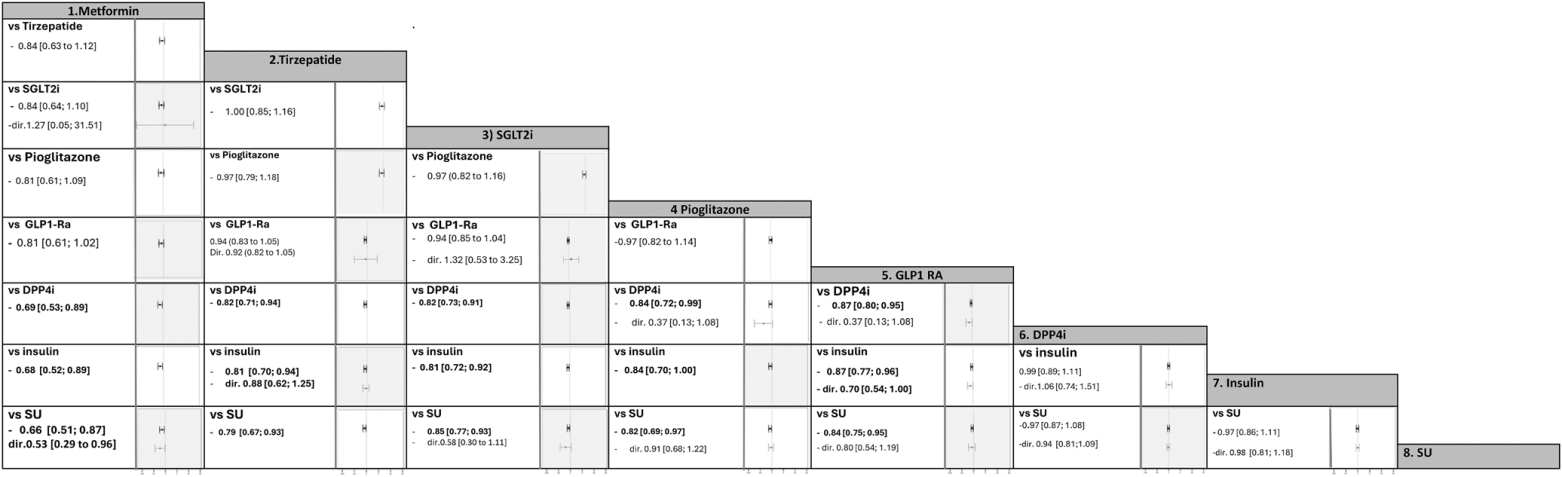

The overall within‐study risk of bias, assessed using the Cochrane risk‐of‐bias tool, was low for most comparisons contributing to the network. Some concerns were identified in specific contrasts, particularly those involving metformin and, to a lesser extent, many comparisons involving insulin, sulfonylureas, or pioglitazone, mainly due to limitations in allocation concealment and outcome reporting (Figure S15). These concerns had a limited impact on the overall network estimates and were incorporated into the CINeMA framework when rating the certainty of evidence (Figure S16).

The confidence of evidence was rated high or moderate for all comparisons including GLP1RA, except those with metformin for which the confidence rating was low; it was also rated high for SGLT2i and tirzepatide, quality was high for all comparisons except pioglitazone, GLP1RA and metformin (moderate), whereas for metformin the certainty of the evidence was mostly rated as low or moderate. Confidence for comparison between insulin and placebo or active comparators was mostly moderate and high for GLP1RA and tirzepatide; moderate to high for pioglitazone; for comparisons between sulfonylureas and placebo, insulin, DPP4i or pioglitazone confidence was moderate, whereas it was low for comparison between sulfonylureas and metformin or insulin and high for comparisons between sulfonylureas and GLP1RA, SGLT2i and tirzepatide (Figure S16).

### 3.3 All‐Cause Mortality

#### 3.3.1 Traditional Meta‐Analyses (By Class)

Funnel plots did not detect a significant publication bias (Figure S17). Tirzepatide, SGLT2i and GLP1RA, but not metformin, were associated with a significant reduction of all‐cause mortality (Figures S18–S20). Results for tirzepatide were largely driven by active‐comparator‐controlled studies, with over 90% of the total result determined by the SURPASS‐CVOT study versus GLP1RA (Figure S20), whereas the result of SGLT2i was largely driven by placebo‐controlled studies (Figure S19). For GLP1RA, the reduction of mortality was statistically significant in placebo‐controlled studies, but not in trials vs. active comparators, where the sample size was smaller; the estimated effect on mortality in active comparator trials was similar to that of placebo‐controlled studies and the difference across subgroups of trials was not statistically significant (Figure S15). DPP4i, pioglitazone, insulin and sulfonylureas did not show significant effects on all‐cause mortality (Figures S21–S24).

#### 3.3.2 Network Meta‐Analysis

The analysis included 91 studies (89 two‐arm studies and two three‐arm studies, of which 13 had zero events and 25 had at least one zero‐event arm), which assessed 10 interventions (nine drug classes and placebo), overall including 18 101 deaths in 256 455 patients. Out of 45 possible pairwise comparisons, 23 comparisons with direct data were retrieved (Figure S25). Tirzepatide (OR 0.72, 0.64–0.82), SGLT2i (OR 0.85, 0.80–0.91) and GLP1RA (OR 0.87, 0.82–0.92) were associated with a significant reduction of all‐cause mortality versus placebo. No effect was observed for other drugs (Figure 2B). In direct comparisons, tirzepatide was superior to GLP1RA and GLP1RA were superior to insulin, DPP4i and sulfonylureas (Table 2). In indirect comparisons, tirzepatide was superior to all other drugs with the only exception of metformin and acarbose; SGLT2i and GLP1RA were superior to insulin, DPP4i and sulfonylureas (Table 2). In the sensitivity analysis (Bayesian meta‐analysis), Tirzepatide, GLP1RA and SGLT2i were associated with a significant reduction of all‐cause mortality versus placebo, whereas results with other drugs were not statistically significant (Figure S26). No inconsistency was found for any comparison (Figure S27).

**TABLE 2:** Frequentist treatment effect: Summary forest plots direct comparisons (in black) and indirect comparisons (in grey) between diabetes drug classes in overall mortality (dir. = direct).

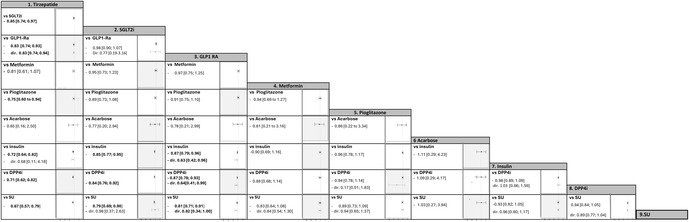

The overall within‐study risk of bias, assessed using the Cochrane risk‐of‐bias tool, was low for most comparisons contributing to the network. Some concerns were identified in several specific contrasts, particularly those involving metformin, insulin, sulfonylureas, or pioglitazone (Figure S28). These concerns had a limited impact on the network estimates. The certainty of evidence for all‐cause mortality, evaluated using the CINeMA framework, is presented in Figure S29. Overall, confidence in the estimates was low for all comparisons involving acarbose, due to imprecision, as only one trial performed with acarbose was available, whereas it was high for most comparisons involving GLP1RA, SGLT2i and tirzepatide versus placebo and most active comparators, with the exception of some lower certainty that we observed in specific cases, mainly driven by imprecision and within‐study bias, especially regarding the UKPDS trials [3, 15]. Evidence for acarbose was consistently rated as low certainty due to imprecision. For GLP1RA, certainty was generally high, with moderate ratings in comparisons versus placebo, metformin, or pioglitazone. For metformin, certainty ranged from low (notably versus tirzepatide or sulfonylureas, mainly due to imprecision and inconsistency) to high (versus DPP4i), with most other comparisons rated as moderate. Certainty of evidence for SGLT2i and tirzepatide was high for most comparisons, except for comparisons involving metformin or pioglitazone, which were rated as moderate. Evidence for insulin and sulfonylureas was generally of moderate certainty, with lower confidence in comparisons involving metformin and higher certainty in comparisons versus GLP1RA, SGLT2i and tirzepatide. Similarly, comparisons involving pioglitazone were mostly supported by moderate certainty, except for higher confidence estimates in selected contrasts. Downgrading of certainty was primarily driven by imprecision and, to a lesser extent, inconsistency, rather than within‐study risk of bias.

### 3.4 Components of MACE

#### 3.4.1 Nonfatal Myocardial Infarction

In the 21 available pairwise comparisons in 61 studies, assessing eight drug classes and placebo, 196 031 patients were enrolled and 7987 non‐fatal myocardial infarctions were reported. Out of 36 possible pairwise comparisons, 21 comparisons with direct data were retrieved. In the principal (frequentist) analysis, SGLT2i, GLP1RA and tirzepatide were associated with a lower incidence of myocardial infarction, whereas no significant effect was observed for pioglitazone, DPP4i, insulin, metformin and sulfonylureas. In indirect comparisons, tirzepatide was associated with a lower incidence of myocardial infarction than DPP4i and sulfonylureas; GLP1RA were associated with a lower incidence of myocardial infarction than DPP4i; SGLT2i were associated with a lower incidence of myocardial infarction than sulfonylureas (Figure S30).

#### 3.4.2 Non‐Fatal Stroke

The NMA included 56 studies, which assessed eight drug classes and placebo, overall including 3320 non‐fatal strokes in 155 019 patients. Out of 36 possible pairwise comparisons, 19 with direct data were retrieved. GLP1RA were associated with a lower incidence of non‐fatal stroke than placebo (Figure 2D), SGLT2i and sulfonylureas and pioglitazone in comparison with sulfonylureas, whereas all the other glucose‐lowering drug classes were not associated with any significant effect on the incidence of non‐fatal stroke (Figure S31).

## 4 Discussion

The present NMA confirms, on a wider evidence base including indirect comparisons, the beneficial effects of SGLT2i and GLP1RA on cardiovascular risk and all‐cause mortality in patients with type 2 diabetes, already reported in traditional meta‐analyses of placebo‐controlled trials [4, 5, 6]. Notably, both classes are superior not only to placebo, but also to several classes of active drugs, such as DPP4i, sulfonylureas and insulin. A significant reduction in the incidence of nonfatal myocardial infarction is observed for both classes, confirming previous meta‐analyses [5, 6], whereas the effect on the risk of nonfatal stroke is significant only for GLP1RA. It can be speculated that the mechanisms responsible for the reduction of cardiovascular risk with either SGLT2i or GLP1RA are partly divergent: although both classes have positive effects on some cardiovascular risk factors, such as blood pressure, blood glucose and body weight [16], GLP1RA could have direct protective effects on vascular walls via the interaction with the endothelial GLP1 receptor, leading to the prevention of atherogenesis [17]. On the other hand, SGLT2i could have a more specific cardiac effect, determined by a reduction of pre‐ and post‐load and consequent decrease in oxygen consumption [18, 19], as well as by enhanced oxygen supply and a shift in substrate metabolism [20].

The assessment of the effects of tirzepatide on cardiovascular risk is methodologically challenging, since the larger available trial assessing cardiovascular outcomes [11] is a non‐inferiority trial versus an active comparator, dulaglutide, with proven cardiovascular benefit [21]. The present NMA shows a significant reduction of cardiovascular risk versus placebo, insulin, DPP4i and sulfonylureas, similar to that observed for SGLT2i and GLP1RA. This result should be interpreted cautiously since it was obtained mainly through indirect comparisons. The reduction of risk versus placebo was significant for myocardial infarction, but not for stroke; however, for the latter, the relative paucity of available data for tirzepatide, generating wide confidence intervals, could have contributed to the result. Notably, the overall reduction of MACE was not greater than with GLP1RA, suggesting that the stimulation of GIP receptor [22] does not play a major role in cardiovascular prevention [23]. Tirzepatide was also associated with a wide reduction in all‐cause mortality versus placebo; with this drug, mortality was significantly lower not only than with DPP4i, insulin and sulfonylureas, but also in comparison with pioglitazone, SGLT2i and GLP1RA. The result is largely driven by the direct comparison with dulaglutide [11]. It can be speculated that the greater reduction of hyperglycemia and/or the more pronounced weight loss in comparison with other drugs, including GLP1RA [24], contributes to an improvement in the prognosis of cardiovascular events, thus reducing cardiovascular and all‐cause mortality.

The positive effect of metformin on cardiovascular risk [4, 25] is confirmed by the present NMA, with significant differences from placebo, DPP4i, sulfonylureas and insulin. On the other hand, data on mortality are not significant. The assessment of the effects of metformin is based on a small number of studies with limited sample size, mostly performed many years ago. In fact, metformin is rarely chosen as a comparator for newer drugs assessing cardiovascular outcomes, maybe because of the high expectations for its effects on cardiovascular risk. The lower quality of parent trials reduces the reliability of the results of metformin on MACE and all‐cause mortality.

Pioglitazone had been previously reported to reduce the incidence of MACE, while increasing the risk of hospitalisations for heart failure [7]. In the present pairwise meta‐analysis, pioglitazone had a significant effect versus all comparators, confirming previous results [8]. In NMA, the beneficial effect of pioglitazone was observed in comparison with placebo, DPP4i, insulin and sulfonylureas, although it was more nuanced, as it was not confirmed by the Bayesian sensitivity analysis. It may be speculated that the antiatherogenic effect of pioglitazone, demonstrated by clinical trials [26] was partly countered by the effects on fluid retention, determining increased myocardial oxygen consumption [27]. Notably, a trial showing a reduction in the risk of stroke determined by treatment with pioglitazone could not be included in the meta‐analysis because it enrolled patients without diabetes [28].

The neutrality of DPP4i with respect to cardiovascular morbidity and all‐cause mortality is confirmed by the present NMA [9], as well as the effects of insulin, which neither increases nor decreases the incidence of MACE and mortality and is also in line with previous reports [29, 30].

The paucity of data does not allow an accurate assessment of the effects of acarbose.

Previous meta‐analyses of clinical trials had reported a significant increase in the incidence of MACE and all‐cause mortality associated with sulfonylureas [10, 25]. The present pairwise meta‐analysis confirms that sulfonylureas are associated with an increased risk of cardiovascular events. This phenomenon can be attributed to the interaction of the drugs of this class with ATP‐dependent potassium channels in cardiomyocytes and cerebral tissues, augmenting the susceptibility to ischemic damage [31, 32] and/or to an increased risk of hypoglycemia [33]. Conversely, in contrast to previous pairwise meta‐analyses [4, 10], in this NMA the observed association between sulfonylureas and the increase in cardiovascular events and all‐cause mortality did not reach statistical significance. It is possible that previous results were partly determined by comparisons with active drugs which reduce cardiovascular events and mortality, such as SGLT2i, GLP1RA, pioglitazone and possibly metformin. Furthermore, our choice to exclude all trials not adjudicating MACE may also have determined a difference in results. A significant publication bias has been detected for sulfonylureas in our pairwise meta‐analysis, warranting caution in the interpretation of results. In addition, the possibility of bias determined by the relatively lower quality of some of the available trials with sulfonylureas should be considered. Furthermore, indirect comparisons in NMA could be affected by residual bias determined by the heterogeneity of trials, potentially explaining differences from pairwise metanalyses.

The results of the present NMA are largely, but not entirely, convergent with those of a recently published analysis, which shares most of the same methods [13]. However, the present analysis includes some recent large‐scale trials, such as the cardiovascular outcome study with tirzepatide [11], which were not yet available when the previous NMA was performed. In addition, this NMA does not include finerenone, which is not indicated for type 2 diabetes, but only for diabetic nephropathy. Furthermore, we excluded short‐term (24–40 weeks) trials and studies without formal adjudication of cardiovascular events, which can induce background noise, possibly attenuating differences in outcomes across treatments.

The strengths of the present analysis include the overall consideration of all drugs indicated for type 2 diabetes, including only trials with adjudication for events for greater certainty of diagnosis. The relatively high number of direct comparisons between active drugs adds robustness to the analysis. Further strengths are the large size of the enrolled samples, the high quality of a large majority of included trials and the low inconsistency across most comparisons. On the other hand, some weaknesses should be recognised. The analysis of drug classes could overlook potential differences across molecules within the same class with respect to cardiovascular risk. Some of the older drugs were assessed mainly based on results of smaller and lower‐quality trials. In addition, not all direct comparisons were available and indirect comparisons estimated by NMA always need to be considered with some caution, as they could be affected by differences in trial design and case mix. In particular, differences in disease duration, glucose control and comorbidities, as well as differences in concurrent treatments in patients enrolled in different trials, could produce distortions which are difficult to eliminate through statistical analysis. Therefore, direct (pairwise) comparisons, where available for appropriate sample size, should be considered more reliable than indirect comparisons. Notably, most of the evidence on the effects of different drugs on cardiovascular events and all‐cause mortality are derived from cardiovascular outcome trials, which enrolled patients with established cardiovascular disease or with multiple risk factors; the applicability of those results to patients at lower cardiovascular risk remains uncertain.

Another methodological issue is the possible impact of differences in glucose control between treatment arms on the incidence of MACE [2]. In fact, HbA1c levels are significantly different between treatment arms in all placebo‐controlled trials with metabolic outcomes and in a large majority cardiovascular outcome trials, with between‐group differences greater than 1% in some instances [11, 34]. Therefore, the positive results with some classes of drugs cannot be automatically interpreted as the demonstration of cardiovascular protection independent of glucose control. On the other hand, the reduction of glucose levels, together with the favourable effects on other risk factors (e.g., blood pressure, body weight, etc.) could be one of the mechanisms underlying the cardiovascular benefits of some classes of drugs. If this is the case, the design of most cardiovascular outcome trials, which established a rescue therapy for patients above reasonable glucose targets, limiting between‐group differences in glucose control, could have produced an actual underestimation of the efficacy of some classes of drugs for cardiovascular prevention in type 2 diabetes.

In conclusion, available data from randomised trials show that SGLT2i, GLP1RA, tirzepatide and (with a lower quality of evidence) metformin and pioglitazone are associated with a reduction of the incidence of cardiovascular events; SGLT2i, GLP1RA and tirzepatide are also associated with a reduced all‐cause mortality.

## Author Contributions

G.A.S. and E.M. were involved in design, data collection, analysis and writing the manuscript. V.V., M.M. and A.L. were involved in data collection, analysis and manuscript revision. A.L. and C.M. were involved in data collection.

## Funding

The authors have nothing to report.

## Conflicts of Interest

G.A.S. has received grants and consultancy fees from Eli Lilly. M.M. has received speaking fees from Astra Zeneca, Bristol Myers Squibb, Boehringer‐Ingelheim, Eli‐Lilly, Merck, Novo Nordisk, Sanofi and Novartis and research grants from Bristol Myers Squibb. E.M. has received consultancy fees from Merck and Novartis, speaking fees from Astra Zeneca, Bristol Myers Squibb, Boehringer‐Ingelheim, Eli‐Lilly, Merck, Novo Nordisk, Sanofi and Novartis and research grants from Merck, Novartis and Takeda. V.V., A.L. and C.M. declares no conflicts of interest.

## Supporting information


**Table S1:** Search strategy.
**Figure S1:** Trial flow summary.
**Figure S2:** Risk of bias graph.
**Figure S3:** Risk of bias summary.
**Table S2:** List of excluded trials, with reasons for exclusion.
**Table S3:** List of included trial. Dur = duration; Age = mean age; DMdur; duration of diabetes; HbA1c = glycated haemoglobin; BMI = Body Mass Index.
**Figure S4:** Funnel plots for MACE.
**Figure S5:** Difference in MACE, GLP1‐RA vs. comparators.
**Figure S6:** Difference in MACE, SGLT2i vs. comparators.
**Figure S7:** Difference in MACE, metformin vs. comparators.
**Figure S8:** Difference in MACE, pioglitazone vs. comparators.
**Figure S9:** Difference in MACE, Tirzepatide vs. comparators.
**Figure S10:** Difference in MACE, DPP4 inhibitors vs. comparators.
**Figure S11:** Difference in MACE, insulin vs. comparators.
**Figure S12:** Difference in MACE, sulfonylureas vs. comparators.
**Figure S13:** Bayesian Meta‐analysis, forest plot: difference in the incidence of MACE between Diabetes drug classes and placebo.
**Figure S15:** MACE within study bias.
**Figure S16:** MACE confidence rating.
**Figure S17:** Overall mortality: funnel plots.
**Figure S18:** Overall mortality: Tirzepatide vs. comparators.
**Figure S19:** Overall mortality: SGLT2i vs. comparators.
**Figure S20:** Overall mortality: GLP1‐RA vs. comparators.
**Figure S21:** Overall mortality: metformin vs. comparators.
**Figure S22:** Overall mortality: DPP4i vs. comparators.
**Figure S23:** Overall mortality: insulin vs. comparators.
**Figure S24:** Overall mortality: SU vs. comparators.
**Figure S25:** Network plot (Panel A: number of trials; panel B: node size is proportional to number of included patients).
**Figure S26:** Bayesian Meta‐analysis, forest plot: difference in overall mortality between Diabetes drug classes and placebo.
**Figure S27:** Nodesplit analysis (inconsistency assessment).
**Figure S28:** Within study bias.
**Figure S29:** Certainty of bias.
**Figure S30:** Non fatal Stroke forest plot.
**Figure S31:** Non Fatal MI.

## Data Availability

Data sharing not applicable to this article as no datasets were generated or analysed during the current study.
