# Supplementary material for: Effect of Medications for Type 2 Diabetes on Cardiovascular Events and Mortality: A Comprehensive Pairwise and Network Meta‐Analysis
Source: Diabetes Obes Metab. 2026 Jul 30;28(10):9403–11. doi: 10.1111/dom.71128 (PMC13538750; doi:10.1111/dom.71128)
Supplement: Supplementary file 1 [file DOM-28-9403-s001.pdf]

EFFECT OF MEDICATIONS FOR TYPE 2 DIABETES ON CARDIOVASCULAR EVENTS AND MORTALITY: A COMPREHENSIVE PAIRWISE AND NETWORK METANALYSIS.

PUBMED
FILTERS: HUMANS, CLINICAL TRIAL, RANDOMIZED CONTROLLED TRIAL
"diabet*"[All Fields] AND ("linagliptin"[Supplementary Concept] OR "linagliptin"[All Fields] OR "linagliptin"[MeSH Terms] OR ("sitagliptin phosphate"[Supplementary Concept] OR "sitagliptin phosphate"[All Fields] OR "sitagliptin"[All Fields] OR "sitagliptin phosphate"[MeSH Terms] OR ("sitagliptin"[All Fields] AND "phosphate"[All Fields]) OR "sitagliptine"[All Fields] OR "sitagliptin s"[All Fields]) OR ("saxagliptin"[Supplementary Concept] OR "saxagliptin"[All Fields]) OR ("alogliptin"[Supplementary Concept] OR "alogliptin"[All Fields]) OR ("vildagliptin"[Supplementary Concept] OR "vildagliptin"[All Fields] OR "vildagliptin"[MeSH Terms]) OR ("metformin"[Supplementary Concept] OR "metformin"[All Fields] OR "metformin"[MeSH Terms] OR "metformine"[All Fields] OR "metformin s"[All Fields] OR "metformins"[All Fields]) OR ("pioglitazone"[Supplementary Concept] OR "pioglitazone"[All Fields] OR "pioglitazone"[MeSH Terms] OR "pioglitazone s"[All Fields] OR "pioglitazones"[All Fields]) OR ("acarbose"[Supplementary Concept] OR "acarbose"[All Fields] OR "acarbose"[MeSH Terms]) OR ("liraglutid"[All Fields] OR "liraglutide"[Supplementary Concept] OR "liraglutide"[All Fields] OR "liraglutide"[MeSH Terms] OR "liraglutide s"[All Fields]) OR ("dulaglutide"[Supplementary Concept] OR "dulaglutide"[All Fields]) OR ("lixisenatide"[Supplementary Concept] OR "lixisenatide"[All Fields]) OR ("semaglutide"[Supplementary Concept] OR "semaglutide"[All Fields]) OR ("exenatide"[Supplementary Concept] OR "exenatide"[All Fields] OR "exenatide"[MeSH Terms] OR "exenatide s"[All Fields]) OR ("tirzepatide"[Supplementary Concept] OR "tirzepatide"[All Fields] OR "tirzepatide"[MeSH Terms]) OR ("glargin"[All Fields] OR "insulin glargine"[Supplementary Concept] OR "insulin glargine"[All Fields] OR "glargine"[All Fields] OR "insulin glargine"[MeSH Terms] OR ("insulin"[All Fields] AND "glargine"[All Fields])) OR ("insulin degludec"[Supplementary Concept] OR "insulin degludec"[All Fields] OR "degludec"[All Fields]) OR ("insulin detemir"[Supplementary Concept] OR "insulin detemir"[All Fields] OR "detemir"[All Fields] OR "insulin detemir"[MeSH Terms] OR ("insulin"[All Fields] AND "detemir"[All Fields])) OR "Icodec"[All Fields] OR ("efsitora"[Supplementary Concept] OR "efsitora"[All Fields]) OR ("glibenclamid"[All Fields] OR "glyburide"[Supplementary Concept] OR "glyburide"[All Fields] OR "glibenclamide"[All Fields] OR "glyburide"[MeSH Terms] OR "glyburide s"[All Fields]) OR ("gliclazide"[Supplementary Concept] OR "gliclazide"[All Fields] OR "gliclazide"[MeSH Terms]) OR ("glimepiride"[Supplementary Concept] OR "glimepiride"[All Fields]) OR ("glipizide"[Supplementary Concept] OR "glipizide"[All Fields] OR "glipizide"[MeSH Terms]) OR ("chlorpropamide"[Supplementary Concept] OR "chlorpropamide"[All Fields] OR "chlorpropamide"[MeSH Terms]) OR ("repaglinide"[Supplementary Concept] OR "repaglinide"[All Fields]) OR ("nateglinide"[Supplementary Concept] OR "nateglinide"[All Fields] OR "nateglinide"[MeSH Terms] OR "nateglinide s"[All Fields]) OR ("dapagliflozin"[Supplementary Concept] OR "dapagliflozin"[All Fields] OR "dapagliflozin s"[All Fields]) OR ("canagliflozin"[Supplementary Concept] OR "canagliflozin"[All Fields] OR "canagliflozin"[MeSH Terms]) OR ("dapagliflozin"[Supplementary Concept] OR "dapagliflozin"[All Fields] OR "dapagliflozin s"[All Fields]) OR ("ertugliflozin"[Supplementary Concept] OR "ertugliflozin"[All Fields]))
linagliptin: "linagliptin"[Supplementary Concept] OR "linagliptin"[All Fields] OR "linagliptin"[MeSH Terms]
sitagliptin: "sitagliptin phosphate"[Supplementary Concept] OR "sitagliptin phosphate"[All Fields] OR "sitagliptin"[All Fields] OR "sitagliptin phosphate"[MeSH Terms] OR ("sitagliptin"[All Fields] AND "phosphate"[All Fields]) OR "sitagliptine"[All Fields] OR "sitagliptin's"[All Fields]
saxagliptin: "saxagliptin"[Supplementary Concept] OR "saxagliptin"[All Fields]
alogliptin: "alogliptin"[Supplementary Concept] OR "alogliptin"[All Fields]
vildagliptin: "vildagliptin"[Supplementary Concept] OR "vildagliptin"[All Fields] OR "vildagliptin"[MeSH Terms]
metformin: "metformin"[Supplementary Concept] OR "metformin"[All Fields] OR "metformin"[MeSH Terms] OR "metformine"[All Fields] OR "metformin's"[All Fields] OR "metformins"[All Fields]
pioglitazone: "pioglitazone"[Supplementary Concept] OR "pioglitazone"[All Fields] OR "pioglitazone"[MeSH Terms] OR "pioglitazone's"[All Fields] OR "pioglitazones"[All Fields]
acarbose: "acarbose"[Supplementary Concept] OR "acarbose"[All Fields] OR "acarbose"[MeSH Terms]
liraglutide: "liraglutid"[All Fields] OR "liraglutide"[Supplementary Concept] OR "liraglutide"[All Fields] OR "liraglutide"[MeSH Terms] OR "liraglutide's"[All Fields]
dulaglutide: "dulaglutide"[Supplementary Concept] OR "dulaglutide"[All Fields]
Lixisenatide: "lixisenatide"[Supplementary Concept] OR "lixisenatide"[All Fields]
Semaglutide: "semaglutide"[Supplementary Concept] OR "semaglutide"[All Fields]

Exenatide: "exenatide"[Supplementary Concept] OR "exenatide"[All Fields] OR "exenatide"[MeSH Terms] OR "exenatide's"[All Fields]<br>Tirzepatide: "tirzepatide"[Supplementary Concept] OR "tirzepatide"[All Fields] OR "tirzepatide"[MeSH Terms]<br>glargine: "glargin"[All Fields] OR "insulin glargine"[Supplementary Concept] OR "insulin glargine"[All Fields] OR "glargine"[All Fields] OR "insulin glargine"[MeSH Terms] OR ("insulin"[All Fields] AND "glargine"[All Fields])<br>degludec: "insulin degludec"[Supplementary Concept] OR "insulin degludec"[All Fields] OR "degludec"[All Fields]<br>detemir: "insulin detemir"[Supplementary Concept] OR "insulin detemir"[All Fields] OR "detemir"[All Fields] OR "insulin detemir"[MeSH Terms] OR ("insulin"[All Fields] AND "detemir"[All Fields])<br>efsitora: "efsitora"[Supplementary Concept] OR "efsitora"[All Fields]<br>glibenclamide: "glibenclamid"[All Fields] OR "glyburide"[Supplementary Concept] OR "glyburide"[All Fields] OR "glibenclamide"[All Fields] OR "glyburide"[MeSH Terms] OR "glyburide's"[All Fields]<br>gliclazide: "gliclazide"[Supplementary Concept] OR "gliclazide"[All Fields] OR "gliclazide"[MeSH Terms]<br>glimepiride: "glimepiride"[Supplementary Concept] OR "glimepiride"[All Fields]<br>glipizide: "glipizide"[Supplementary Concept] OR "glipizide"[All Fields] OR "glipizide"[MeSH Terms]<br>chlorpropamide: "chlorpropamide"[Supplementary Concept] OR "chlorpropamide"[All Fields] OR "chlorpropamide"[MeSH Terms]<br>repaglinide: "repaglinide"[Supplementary Concept] OR "repaglinide"[All Fields]<br>nateglinide: "nateglinide"[Supplementary Concept] OR "nateglinide"[All Fields] OR "nateglinide"[MeSH Terms] OR "nateglinide's"[All Fields]<br>dapagliflozin: "dapagliflozin"[Supplementary Concept] OR "dapagliflozin"[All Fields] OR "dapagliflozin's"[All Fields]<br>canagliflozin: "canagliflozin"[Supplementary Concept] OR "canagliflozin"[All Fields] OR "canagliflozin"[MeSH Terms]<br>dapagliflozin: "dapagliflozin"[Supplementary Concept] OR "dapagliflozin"[All Fields] OR "dapagliflozin's"[All Fields]<br>ertugliflozin: "ertugliflozin"[Supplementary Concept] OR "ertugliflozin"[All Fields]
**Clinicaltrials.gov:** (linagliptin OR sitagliptin OR saxagliptin OR alogliptin OR vildagliptin OR metformin OR pioglitazone OR acarbose OR liraglutide OR dulaglutide OR Lixisenatide OR Semaglutide OR Exenatide OR Tirzepatide OR glargine OR degludec OR detemir OR Icodec OR efsitora OR glibenclamide OR gliclazide OR glimepiride OR glipizide OR chlorpropamide OR repaglinide OR nateglinide OR dapagliflozin OR canagliflozin OR dapagliflozin OR ertugliflozin) Active, not recruiting, Completed, Terminated Studies | Interventional Studies.
**Cochrane database** results restricted to clinical trials (diabet*) AND (linagliptin OR sitagliptin OR saxagliptin OR alogliptin OR vildagliptin OR metformin OR pioglitazone OR acarbose OR liraglutide OR dulaglutide OR Lixisenatide OR Semaglutide OR Exenatide OR Tirzepatide OR glargine OR degludec OR detemir OR Icodec OR efsitora OR glibenclamide OR gliclazide OR glimepiride OR glipizide OR chlorpropamide OR repaglinide OR nateglinide OR dapagliflozin OR canagliflozin OR dapagliflozin OR ertugliflozin) in Title Abstract Keyword - (Word variations have been searched)
**Embase** 'linagliptin'/exp OR linagliptin OR 'sitagliptin'/exp OR sitagliptin OR 'saxagliptin'/exp OR saxagliptin OR 'alogliptin'/exp OR alogliptin OR 'vildagliptin'/exp OR vildagliptin OR 'metformin'/exp OR metformin OR 'pioglitazone'/exp OR pioglitazone OR 'acarbose'/exp OR acarbose OR 'liraglutide'/exp OR liraglutide OR 'dulaglutide'/exp OR dulaglutide OR 'lixisenatide'/exp OR lixisenatide OR 'semaglutide'/exp OR semaglutide OR 'exenatide'/exp OR exenatide OR 'tirzepatide'/exp OR tirzepatide OR 'glargine'/exp OR glargine OR 'degludec'/exp OR degludec OR 'detemir'/exp OR detemir OR icodec OR efsitora OR 'glibenclamide'/exp OR glibenclamide OR 'gliclazide'/exp OR gliclazide OR 'glimepiride'/exp OR glimepiride OR 'glipizide'/exp OR glipizide OR 'chlorpropamide'/exp OR chlorpropamide OR 'repaglinide'/exp OR repaglinide OR 'nateglinide'/exp OR nateglinide OR 'canagliflozin'/exp OR canagliflozin OR 'dapagliflozin'/exp OR dapagliflozin OR 'ertugliflozin'/exp OR ertugliflozinAND ('(randomized controlled trial)'/exp OR '(randomized controlled trial)') AND ('human'/exp OR 'human'**)**

.

Table S1: Search strategy

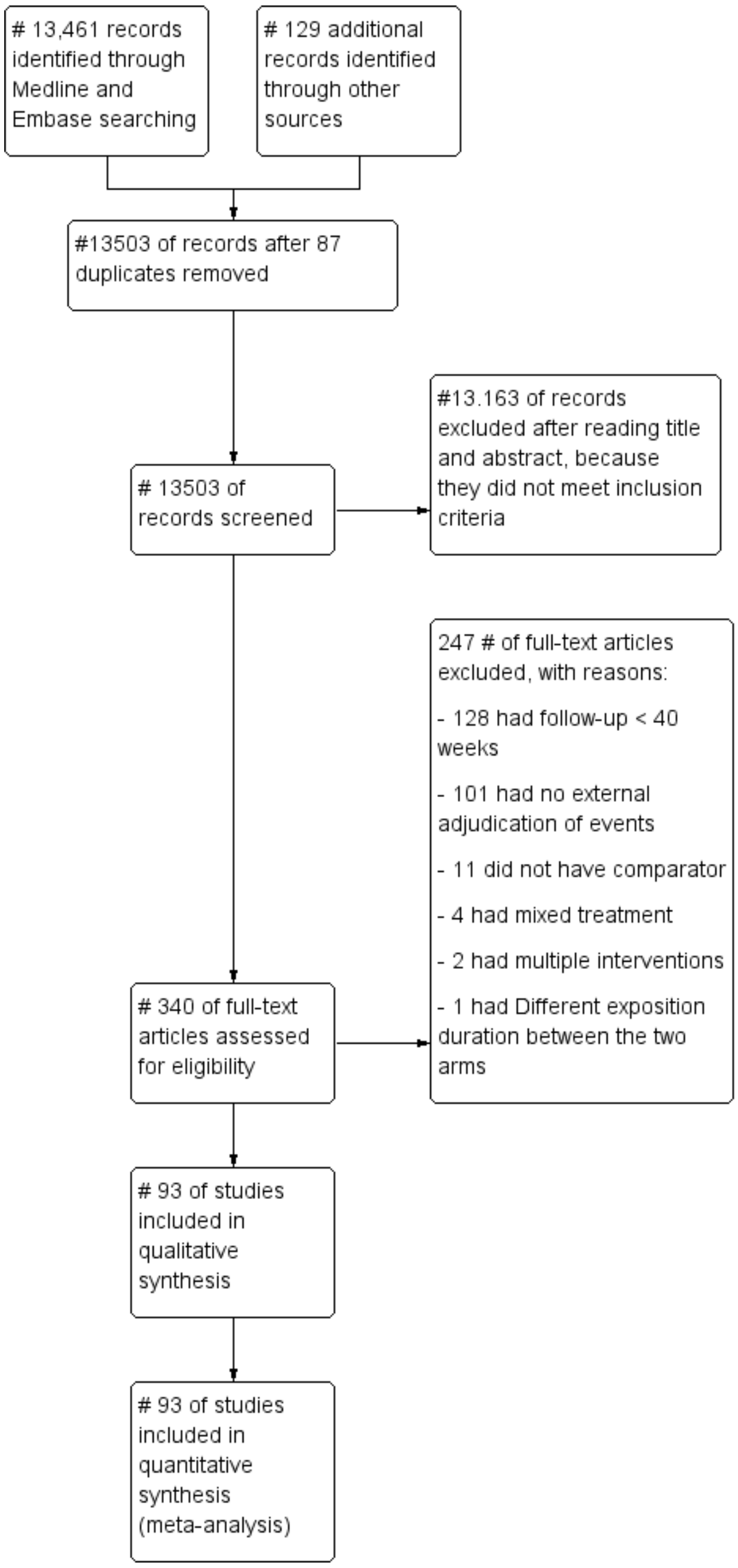

Figure S1:
Trial flow summary.

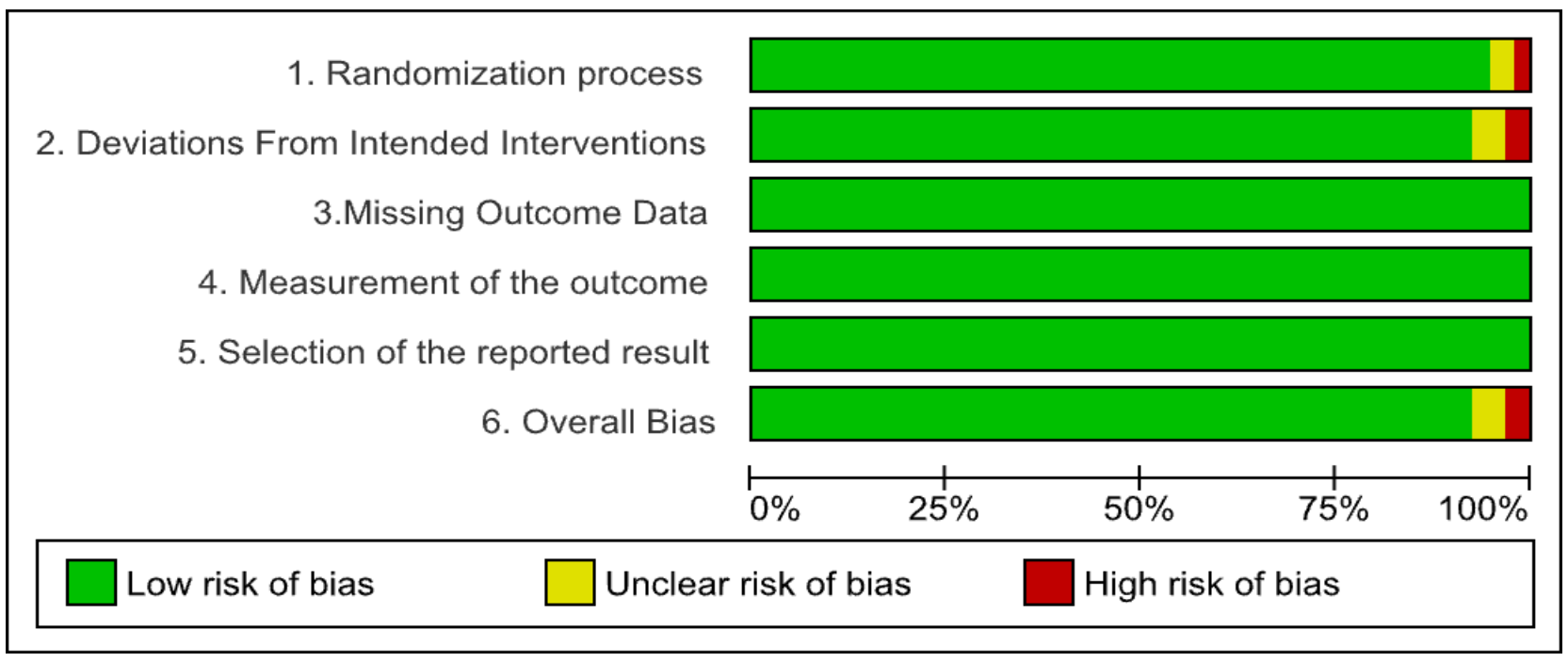

Figure S2:
Risk of bias graph

	1. Randomization process	2. Deviations From Intended Interventions	3.Missing Outcome Data	4. Measurement of the outcome	5. Selection of the reported result	6. Overall Bias
Ahren 2014	+	+	+	+	+	+
Ahren 2017	+	+	+	+	+	+
Arjona-Ferreira 2013	+	+	+	+	+	+
Billings 2025	+	+	+	+	+	+
Blonde 2015	+	+	+	+	+	+
Bolli 2009	+	+	+	+	+	+
Bonaca 2025	+	+	+	+	+	+
Buse 2020	+	+	+	+	+	+
Cannon 2020	+	+	+	+	+	+
Cioffi 2021	+	+	+	+	+	+
Dahl 2022	+	+	+	+	+	+
Davies 2015	+	+	+	+	+	+
Davies 2021	+	+	+	+	+	+
Del Prato 2014	+	+	+	+	+	+
Del Prato 2021	+	+	+	+	+	+
Dormandy 2005	+	+	+	+	+	+
Ferrannini 2013	+	?	+	+	+	?
Foley 2009	+	?	+	+	+	?
Frias 2021	+	+	+	+	+	+
Gallwitz 2012	+	+	+	+	+	+
Gao 2023	+	+	+	+	+	+
Garvey 2023	+	+	+	+	+	+
Gerstein 2012	?	?	+	+	+	?
Gerstein 2019	+	+	+	+	+	+
Giles 2010	+	+	+	+	+	+
Giorgino 2015	+	+	+	+	+	+
Gouch 2015	+	+	+	+	+	+
Green 2015	+	+	+	+	+	+
Green 2024	+	+	+	+	+	+
Guo 2025	+	+	+	+	+	+
Henry 2014	+	+	+	+	+	+
Holman 1999	+	+	+	+	+	+
Holman 2017	+	+	+	+	+	+
Home 2015	+	+	+	+	+	+
Hong 2013	+	+	+	+	+	+
Husain 2019	+	+	+	+	+	+
Inagaky 2025	+	+	+	+	+	+
Inzucchi 2022	+	+	+	+	+	+
Jabbour 2018	+	+	+	+	+	+
Jabbour 2020	+	+	+	+	+	+
Kahn 2006	+	+	+	+	+	+
Kaku 2019	+	+	+	+	+	+
Kaku 2019 bis	+	+	+	+	+	+
Kawamori 2018	+	+	+	+	+	+
Kosiborod 2024	+	+	+	+	+	+
Li 2020	+	+	+	+	+	+
Lingvay 2019	+	+	+	+	+	+
Ludvik 2021	+	+	+	+	+	+
Marso 2016	+	+	+	+	+	+
Marso 2016 bis	+	+	+	+	+	+
Matthei 2016	+	+	+	+	+	+
Matthews 2019	+	+	+	+	+	+
Mazzone 2006	+	+	+	+	+	+
Mc Gill 2012	+	+	+	+	+	+
Mc Guire 2025	+	+	+	+	+	+
Mc Murray 2018	+	+	+	+	+	+
Mita 2016	+	+	+	+	+	+
Mita 2016 bis	+	+	+	+	+	+
Miyagawa 2015	+	+	+	+	+	+
Nauck 2016	+	+	+	+	+	+
Neal 2017	+	+	+	+	+	+
Nicholls 2025	+	+	+	+	+	+
Nissen 2008	+	+	+	+	+	+
Oyama 2016	+	+	+	+	+	+
Perkovic 2019	+	+	+	+	+	+
Perkovic 2024	+	+	+	+	+	+
Perrson 2021	+	+	+	+	+	+
Petrie 2021	+	+	+	+	+	+
Pfeffer 2015	+	+	+	+	+	+
Pratley 2019	+	+	+	+	+	+
Ridderstrale 2018	+	+	+	+	+	+
Rodbard 2019	+	+	+	+	+	+
Rosenstock 2019	+	+	+	+	+	+
Rosenstock 2019 bis	+	+	+	+	+	+
Rosenstock 2019 ter	+	+	+	+	+	+
Rosenstock 2021	+	+	+	+	+	+
Rosenstock 2023 bis	+	+	+	+	+	+
Scirica 2013	+	+	+	+	+	+
Tuttle 2018	+	+	+	+	+	+
UKPDS 33 1998	?	−	+	+	+	−
UKPDS 34-1 1998	−	−	+	+	+	−
UKPDS 34-2 1998	−	−	+	+	+	−
Vaccaro 2017	?	?	+	+	+	?
Wang 2019 a	+	+	+	+	+	+
Weinstock 2015	+	+	+	+	+	+
White 2013	+	+	+	+	+	+
Wiviott 2018	+	+	+	+	+	+
Yabe 2020	+	+	+	+	+	+
Yamada 2020	+	+	+	+	+	+
Yki-jarvinen 2013	+	+	+	+	+	+
Yoshii 2014	+	+	+	+	+	+
Zinman 2015	+	+	+	+	+	+
Zinman 2019	+	+	+	+	+	+

Figure S3: *Risk of bias summary*

#	Trial Excluded	Reason	#	Trial Excluded	Reason
1	Lingvay 2023	follow-up <40 weeks	81	Sivalingam 2023	No adjudication
2	Morschel 2023	follow-up <40 weeks	82	Seijas-Amigo 2025	No adjudication
3	Lingvay 2025	No adjudication	83	Sahay 2023	Multiple interventions
4	Philis-Tsimikas 2023	follow-up <40 weeks	84	Park 2023	No adjudication
5	Lingvay 2025 ter	No adjudication	85	Huang 2025	No adjudication
6	Pieber 2023	No adjudication	86	Bhosle 2025	No adjudication
7	Eto 2024	follow-up <40 weeks	87	Albulushi 2025	No adjudication
8	Watada 2024	No adjudication	88	Eriksson 2026	No adjudication
9	Hovelmann 2024	No adjudication	89	Miyamoto 2024	No adjudication
10	Westergaard 2024	follow-up <40 weeks	90	Wada 2022	No adjudication
11	Bergenstal 2025	follow-up <40 weeks	91	Shields 2023	No adjudication
12	Roell 2025	follow-up <40 weeks	92	Laffel 2023	Multiple interventions
13	Crutchlow 2025	follow-up <40 weeks	93	Yabe 2023	No adjudication
14	Thomas R Pieber 2025	follow-up <40 weeks	94	Petrie 2024	No adjudication
15	Ping Feng 2023	follow-up <40 weeks	95	Karetnikova 2022	No adjudication
16	Snaith 2025	follow-up <40 weeks	96	Werida 2022	follow-up <40 weeks
17	Hankosky 2025 bis	No adjudication	97	Middleton 2022	No adjudication
18	Heise 2022	follow-up <40 weeks	98	Morioka 2024	No adjudication
19	Pirro 2022	follow-up <40 weeks	99	Iijima 2023	follow-up <40 weeks
20	Kadowaki 2022	No comparator	100	Liu 2025	follow-up <40 weeks
21	Furihata 2022	follow-up <40 weeks	101	Wang 2023	follow-up <40 weeks
22	Hannon 2025	No adjudication	102	Jiang 2022	follow-up <40 weeks
23	Mather 2024	follow-up <40 weeks	103	Yang He 2025	No adjudication
24	Martin 2025	follow-up <40 weeks	104	Kimura 2023	follow-up <40 weeks
25	Frias 2021	follow-up <40 weeks	105	Rosenstock 2023	follow-up <40 weeks
26	Rosenstock 2021	follow-up <40 weeks	106	Frias 2023	follow-up <40 weeks
27	Thomas 2020	follow-up <40 weeks	107	Takahashi 2023	No adjudication
28	Frias 2020	follow-up <40 weeks	108	Arslanian 2022	follow-up <40 weeks
29	Hartman 2020	follow-up <40 weeks	109	Takeshita 2022	follow-up <40 weeks
30	Wilson 2020	follow-up <40 weeks	110	Garvey 2024	No adjudication
31	Coskun 2018	follow-up <40 weeks	111	Wang 2022	follow-up <40 weeks
32	Yuki Oe 2025	follow-up <40 weeks	112	Caruso 2024	follow-up <40 weeks
33	Crisafulli 2025	No adjudication	113	Galindo 2023	No adjudication
34	Frias 2023	follow-up <40 weeks	114	Kawaguchi 2024	follow-up <40 weeks
35	Kazda 2023	follow-up <40 weeks	115	Chowdhary 2024	No adjudication
36	Katz 2025	follow-up <40 weeks	116	Lin 2024	follow-up <40 weeks
37	Tanner 2025	No adjudication	117	Takahashi 2024	follow-up <40 weeks
38	Larios 2025	No adjudication	118	Katogiannis 2024	follow-up <40 weeks
39	Yun Wang 2025	No adjudication	119	Dejgaard 2024	No adjudication
40	Blevins 2025	follow-up <40 weeks	120	Nathan 2022	No adjudication
41	Barakat 2025	No adjudication	121	Caruso 2025	No adjudication
42	Buse 2025	No adjudication	122	Iacobellis 2024	follow-up <40 weeks
43	Wang 2024	follow-up <40 weeks	123	Cheng 2022	follow-up <40 weeks
44	Ji 2024	follow-up <40 weeks	124	Nielsen 2021	follow-up <40 weeks
45	Zinman 2019	follow-up <40 weeks	125	Lòpez-Cano 2022	follow-up <40 weeks
46	Capehorn 2020	follow-up <40 weeks	126	Zhu 2026	follow-up <40 weeks
47	Kellerer 2022	No adjudication	127	Dietsche 2024	follow-up <40 weeks
48	Frias 2021	No comparator	128	Ma 2024	follow-up <40 weeks
49	Aroda 2023	No comparator	129	Wexler 2023	No adjudication
50	Gabe 2024	follow-up <40 weeks	130	Glaros 2025	follow-up <40 weeks
51	Takahashi 2023	follow-up <40 weeks	131	Ou 2022	follow-up <40 weeks
52	Lingvay 2025	No adjudication	132	Loganathan 2022	follow-up <40 weeks
53	Aroda 2025	No adjudication	133	Foghsgaard 2022	No adjudication
54	Oluwaseun 2024	follow-up <40 weeks	134	Yu 2022	follow-up <40 weeks
55	Manubolu 2024	No adjudication	135	Punthakee 2024	follow-up <40 weeks
56	Petković-Dabić	follow-up <40 weeks	136	Meng 2023	follow-up <40 weeks
57	Tóth 2025	No adjudication	137	Unger 2021	follow-up <40 weeks
58	Gullaksen 2023	follow-up <40 weeks	138	Harrison 2025	follow-up <40 weeks
59	Kimura 2023	follow-up <40 weeks	139	Jiang 2022	follow-up <40 weeks
60	James 2025	No adjudication	140	Sindhvananda 2023	follow-up <40 weeks
61	Sivalingam 2024	No adjudication	141	Seino 2022	No comparator
62	Dwibedi 2024	follow-up <40 weeks	142	Iijima 2022	No comparator
63	Vernstrøm 2024	follow-up <40 weeks	143	Krishnan 2023	No comparator
64	Ingersen 2023	follow-up <40 weeks	144	Middleton 2021	No adjudication
65	Buse 2025 bis	No comparator	145	Luchsinger 2021	No adjudication
66	Iijima 2023	follow-up <40 weeks	146	Oosterom-Eijmael 2024	follow-up <40 weeks
67	Furusawa 2023	follow-up <40 weeks	147	Zhaou 2022	follow-up <40 weeks

68	Rodriguez 2025	follow-up <40 weeks	148	Seaquist 2024	No adjudication
69	Yang 2024	Mixed treatment	149	Eghbali 2024	follow-up <40 weeks
70	Ji 2021	follow-up <40 weeks	150	Kim 2020	follow-up <40 weeks
71	Gibbons 2021	Mixed treatment	151	Araki 2013	follow-up <40 weeks
72	Dahl 2021	Mixed treatment	152	Laakso 2015	Different exposition periods
73	Sorli 2017	follow-up <40 weeks	153	Liu 2024	No adjudication
74	Aroda 2017	follow-up <40 weeks	154	Haak 2013	No adjudication
75	Rodbard 2018	follow-up <40 weeks	155	Taskinen 2011	follow-up <40 weeks
76	Aroda 2019	follow-up <40 weeks	156	Lewin 2015	No adjudication
77	Mosenzon 2019	follow-up <40 weeks	157	Linong Ji 2015	No adjudication
78	Kaku 2018	No comparator	158	Cooper 2019	follow-up <40 weeks
79	Korakas 2024	No adjudication	159	Araki 2019	No adjudication
80	Gad 2024	No adjudication	160	Yagoglu 2020	No adjudication
161	Katsuno 2021	No adjudication	205	Matthews 2010	No adjudication
162	Nagao 2023	No adjudication	206	Dei Cas 2017	No adjudication
163	Sahay 2023	follow-up <40 weeks	207	Derosa 2012	No adjudication
164	Ji 2024	follow-up <40 weeks	208	Göke 2008	No adjudication
165	Singh 2024	follow-up <40 weeks	209	Kanazawa 2017	No adjudication
166	Wang 2022	follow-up <40 weeks	210	Schweizer 2007	No adjudication
167	Zhou 2022	follow-up <40 weeks	211	Scherbaum 2008	No adjudication
168	Nagao 2024	follow-up <40 weeks	212	Foley 2011	No adjudication
169	Alamdari 2025	follow-up <40 weeks	213	Mathieu 2013	No adjudication
170	Kim 2024	Mixed treatment	214	Foley 2009	No adjudication
171	Takuma 2023	follow-up <40 weeks	215	Kothny 2012	No adjudication
172	Qin 2025	follow-up <40 weeks	216	Mari 2008	No adjudication
173	Barchetta 2025	No adjudication	217	Ahren 2007	No adjudication
174	Shankar 2021	No adjudication	218	Fonseca 2008	No adjudication
175	Gracia-Ramos 2024	follow-up <40 weeks	219	Ahren 2005	No adjudication
176	Wang 2021	follow-up <40 weeks	220	Koshino 2023	follow-up <40 weeks
177	Zhou 2022	follow-up <40 weeks	221	Chen 2023	follow-up <40 weeks
178	Singh 2025	follow-up <40 weeks	222	Wangnoo 2025	follow-up <40 weeks
179	McInnes 2022	follow-up <40 weeks	223	Frias 2021	No adjudication
180	Lee 2022	follow-up <40 weeks	224	Shehadeh 2025	No adjudication
181	Kang 2023	follow-up <40 weeks	225	Scheen 2010	No adjudication
182	Yaribeygi 2025	follow-up <40 weeks	226	Hermans 2012	follow-up <40 weeks
183	Wilson 2022	follow-up <40 weeks	227	Iqbal 2014	No adjudication
184	Cordiner 2024	follow-up <40 weeks	228	C-J Li 2014	follow-up <40 weeks
185	Wewer Albrechtsen 2022	follow-up <40 weeks	229	Chetan Karyekar 2011	follow-up <40 weeks
186	Shields 2022	follow-up <40 weeks	230	Barnett 2013	No adjudication
187	Zhou 2024	follow-up <40 weeks	231	DeFronzo 2009	follow-up <40 weeks
188	Wang 2024	follow-up <40 weeks	232	Yu Pan 2012	follow-up <40 weeks
189	Khan 2023	follow-up <40 weeks	233	Muller-Wieland 2018	No adjudication
190	Gao 2022	follow-up <40 weeks	234	Yang 2011	follow-up <40 weeks
191	Kim 2022	follow-up <40 weeks	235	Hollander 2011	No adjudication
192	Seino 2011	No comparator	236	Handelsman 2019	No adjudication
193	Kaku 2014	No adjudication	237	Tianwei Gu 2019	No adjudication
194	Bosi 2011	No adjudication	238	Rosenstock 2015	follow-up <40 weeks
195	Seino 2011 bis	No comparator	239	Frias 2020	No adjudication
196	Fujii 2013	No adjudication	240	Pfutzner 2011	No adjudication
197	Kusunoki 2015	No adjudication	241	S Matthaei 2016	No adjudication
198	Matthews 2010	No adjudication	242	Derosa 2010	No adjudication
199	Dei Cas 2017	No adjudication	243	Ristic 2006	follow-up <40 weeks
200	Derosa 2012	No adjudication	244	Charbonnel 2005	No adjudication
201	Göke 2008	No adjudication	245	Charbonnel 2005 bis	No adjudication
202	Kanazawa 2017	No adjudication	246	Tan 2005	No adjudication
203	Schweizer 2007	No adjudication	247	Tessier 2009	No adjudication
204	Scherbaum 2008	Scherbaum 2008			

Table S2: list of excluded trials, with reasons for exclusion.

Paper	*Study*	Intervention	Comparator(s)	Dur	Age	DMdur	HbA1c	BMI
Ahren 2014 [1]	*HARMONY 3*	Sitagliptin	Glimepiride, Placebo	104	55	6	8.1	32.7
Ahren 2017 [2]	*SUSTAIN-2*	Semaglutide	Sitagliptin	56	55	6.5	8.1	32.5
Arjona-Ferreira 2013 [3]	*MK0431-073*	Sitagliptin	Glipizide	54	59	17	7.8	26.8
Billings 2025 [4]	*SURPASS-SWITCH*	Tirzepatide	Dulaglutide	40	57.6	11.2	7.8	34.3
Blonde 2015 [5]	*AWARD-4*	Dulaglutide	Glargine	52	59.5	12.3	8.5	32.5
Bolli 2009 [6]	*CLAF237A2354*	Vildagliptin	Pioglitazone	52	57	6	8.4	32.1
Bonaca 2025 [7]	*STRIDE*	Semaglutide	Placebo	52	68	12.2	7.1	28.6
Buse 2020 [8]	*PIONEER-7*	Sitagliptin	Semaglutide	52	57	9	7.9	31
Cannon 2020 [9]	*VERTIS-CV*	Ertugliflozin	Placebo	182	64	13	8.2	31.9
Cioffi 2021 [10]	*DYDA 2*	Linagliptin	Placebo	48	69	7	6.4	30
Dahl 2022 [11]	*SURPASS-5*	Tirzepatide	Placebo	40	60.7	13.3	8.3	33.4
Davies 2015 [12]	*SCALE*	Liraglutide	Placebo	56	55	7	7.9	37.4
Davies 2021 [13]	*STEP 2*	Semaglutide	Placebo	68	55	8	8.1	35.7
Del Prato 2014 [14]		Alogliptin	Glipizide	104	55.4	5.5	7.6	31.3
Del Prato 2021 [15]	*SURPASS-4*	Tirzepatide	Glargine	104	63.6	10.5	8.5	32.6
Dormandy 2005 [16]		Pioglitazone	Placebo	150	62	8	7.8	30.8
Ferrannini 2013 [17]		Empagliflozin	Metformin, Sitagliptin	78	58	NR	7.9	30.1
Foley 2009 [18]	*CLAF237A2310*	Vildagliptin	Gliclazide	320	54	2	8.6	30.7
Frias 2021 [19]	*SURPASS-2*	Tirzepatide	Semaglutide	40	56.6	8.6	8.3	34.2
Gallwitz 2012 [20]	*EUREXA*	Exenatide	Glimepiride	208	56	6	7.4	32.4
Gao 2023 [21]	*SURPASS-COMBO*	Tirzepatide	Glargine	40	54.1	7.7	8.7	27.9
Garvey 2023 [22]	*SURMOUNT-2*	Tirzepatide	Placebo	72	54.2	8.5	8	36.1
Gerstein 2012 [23]	*ORIGIN*	Glargine	Placebo	322	64	6.4	5	29.8
Gerstein 2019 [24]	*REWIND*	Dulaglutide	Placebo	281	66	10	7.3	32.3
Giles 2010 [25]		Pioglitazone	Glibenclamide	52	64	NR	8.6	NR
Giorgino 2015 [26]	*AWARD-2*	Dulaglutide	Glargine	78	57	9	8.1	31.3
Gough 2015 [27]	*DUAL-I*	Liraglutide	Degludec	52	55	7	8.3	31.2
Green 2015 [28]	*TECOS*	Sitagliptin	Placebo	156	65.5	11.6	7.2	30.2
Green 2024 [29]	*GRADE*	Liraglutide, Sitagliptin	Glimepiride, Glargine	260	57.2	4	7	34.3
Guo 2025 [30]	*SURPASS-CN-INS*	Tirzepatide	Placebo	40	56.7	11.6	8.7	27.1
Henry 2014 [31]		Sitagliptin	Pioglitazone, Placebo	54	57	NR	NR	NR
Holman 1999[32]	*UKPDS 44*	Acarbose	Placebo	156	59	7.6	7.9	NR
Holman 2017 [33]	*EXSCEL*	Exenatide LAR	Placebo	166	62	12	8	31.7
Home 2015 [34]		Pioglitazone	Placebo	156	56	9	8.3	32.2
Hong 2013 [35]	*SPREAD-DIMCAD*	Metformin	Glipizide	104	63	6	7.6	25.1
Husain 2019 [36]	*PIONEER 6*	Semaglutide	Placebo	68	66	15	8.2	32.0
Inagaki 2025[37]	*SURPASS J-MONO*	Tirzepatide	Dulaglutide	52	56.6	4.8	8.2	28.1
Inzucchi 2022[38]	*DELIVER*	Dapagliflozin	Placebo	120	71	9.2	7.4	30.9
Jabbour 2020 [39]	*DURATION-8*	Exenatide LAR	Placebo, DAPA	104	54	7.4	9.3	32.7
Kahn 2006 [40]	*ADOPT*	Metformin	Glibenclamide	188	57	NR	NR	32.1
Kaku 2019 [41]	*Dual japanese*	Liraglutide	Degludec, Placebo	52	57.2	9.7	8.4	26.5
Kawamori 2018 [42]	*Empalina Japanese*	Empagliflozin	Placebo	52	59.9	8.9	8.3	26.2
Kosiborod 2024 [43]	*STEP-HFpEF DM*	Semaglutide	Placebo	52	69.5	8	6.8	36.9
Li 2020 [44]		Sitagliptin	None	76	60	7	8	25
Lingvay 2019 [45]	*SUSTAIN-8*	Semaglutide	Canagliflozin	52	56	7	8.3	32.3
Ludvik 2021 [46]	*SURPASS-3*	Tirzepatide	Degludec	52	57.4	8.4	8.2	33.5
Marso 2016 [47]	*LEADER*	Liraglutide	Placebo	198	64	13	8.7	32.5
Marso 2016 bis [48]	*SUSTAIN-6*	Semaglutide	Placebo	68	66	15	8.2	32
Marso 2017 [49]	*DEVOTE*	Degludec	Glargine	104	65	16.4	8.4	33.6
Matthaei 2015 [50]		Saxagliptin	Placebo	52	54.6	7.7	7.9	31.4
Matthews 2019 [51]		Vildagliptin	Placebo	259	54	3	6.7	31.1
Mazzone 2006 [52]		Pioglitazone	Glimepiride	72	60	8	7.4	32
McGill 2012 [53]		Linagliptin	Placebo	52	64.4	NR	8.2	32
McGuire 2025[54]	*SOUL*	Semaglutide	Placebo	245	66.1	14.7	8	31.1
McMurray 2018 [55]	*VIVIDD STUDY*	Vildagliptin	Placebo	52	63	9.3	7.8	29.4
Mita 2016 [56]	*SPEAD-A trial*	Alogliptin	None	104	64.6	8.6	7.3	24.8
Mita 2016 bis [57]	*SPIKE*	Sitagliptin	None	104	63.7	17.3	8.1	25
Miyagawa 2015 [58]		Dulaglutide	Placebo	52	58	6.5	8.1	25

Nauck 2016 [59]		Linagliptin	Pioglitazone	NR	56.5	NR	8.1	32.8
Neal 2017 [60]		Canagliflozin	Placebo	52	62	13	8.2	32.1
Nicholls 2025[61]	*SURPASS-CVOT*	Tirzepatide	Dulaglutide	208	64	14.8	8.4	32.6
Nissen 2008 [62]	*PERISCOPE*	Pioglitazone	Glimepiride	78	60	6	7.4	32
Oyama 2016 [63]	*PROLOGUE*	Sitagliptin	Placebo	96	69.35	NR	7.0	25.1
Perkovic 2019 [64]	*CREDENCE*	Canagliflozin	Placebo	52	62	13	8.2	32.1
Perkovic 2024 [65]	*FLOW*	semaglutide	Placebo	177	66.6	NR	7.8	32
Persson 2021 [66]	*DAPA-CKD*	Dapagliflozin	Placebo	125	64.4	13.8	7.8	30.3
Petrie 2021 [67]	*DAPA-HF*	Dapagliflozin	Placebo	385	66	NR	7.4	29.3
Pfeffer 2015 [68]	*ELIXA*	Lixisenatide	Placebo	300	60	9	7.6	30.1
Pratley 2019 [69]	*PIONEER-4*	Liraglutide	Placebo	52	56	8	8	33
Ridderstrale 2018 [70]		Glimepiride	Empagliflozin	104	57	NR	7.8	30.3
Rodbard 2019 [71]	*PIONEER 2*	semaglutide	Empagliflozin	52	58	7.4	8.1	32.8
Rosenstock 2019 [72]		Linagliptin	Placebo	114	66	14.7	7.9	31.3
Rosenstock 2019a [73]	*PIONEER 3*	Semaglutide	Sitagliptin	78	58	8.5	8.3	32.5
Rosenstock 2019 b [74]		Linagliptin	Glimepiride	307	64	6.2	7.2	30.1
Rosenstock 2021[75]	*SURPASS-1*	Tirzepatide	Placebo	40	54.1	4.7	7.9	31.9
Rosenstock2023 [76]	*SURPASS-6*	Tirzepatide	Lispro	52	58.8	13.8	8.8	33.2
Scirica 2013 [77]	*SAVOR-TIMI*	Saxagliptin	Placebo	109	65	12	8	31.1
Tuttle 2018 [78]	*AWARD-7*	Dulaglutide	Glargine	52	64.5	18	8.6	32.3
UKPDS 33 1998 [79]	*UKPDS 33*	insulin	Sulfonylurea, placebo	546	53	0	7	28
UKPDS 34 1998 [80]	*UKPDS 34*	Metformin	Sulfonylurea, placebo	343	53	7.1	7.3	31.4
Vaccaro 2017 [81]	*TOSCA.IT*	Pioglitazone	Sulfonylurea	248	62	8	7.7	30.3
Wang 2019 a [82]		Dulaglutide	Glargine	52	62	7	8.6	25
Weinstock 2015 [83]	*AWARD-5; GBCF*	Dulaglutide	Sitagliptin	104	54	7	8.1	31
White 2013 [84]	*EXAMINE*	Alogliptin	Placebo	94	61	7	8	28.7
Wiviott 2018 [85]	*DECLARE–TIMI 58*	Dapaglifozin	Placebo	218	64	10	8.3	32
Yabe 2020 [86]	*PIONEER 10*	Semaglutide	Dulaglutide	52	58	9.4	8.3	26.2
Yamada 2020 [87]	*PIONEER 9*	Semaglutide	Placebo	52	59.5	7.8	8.2	25.7
Yki-jarvinen 2013 [88]		Linagliptin	Placebo	52	60	NR	8.3	31
Yoshii 2014 [89]		Pioglitazone	Placebo	52	69	11	7.4	24.2
Zinman 2015 [90]	*EMPA-REG OUTCOME*	Empaglifozin	Placebo	161	63	NR	8.1	30.6
Zinman 2019[91]	PIONEER-8	Semaglutide	Placebo	52	61	15	8.2	31

Table S3: list of included trial. Dur= duration; Age= mean age; DMdur; duration of diabetes; HbA1c=glycated hemoglobin; BMI= Body Mass Index.

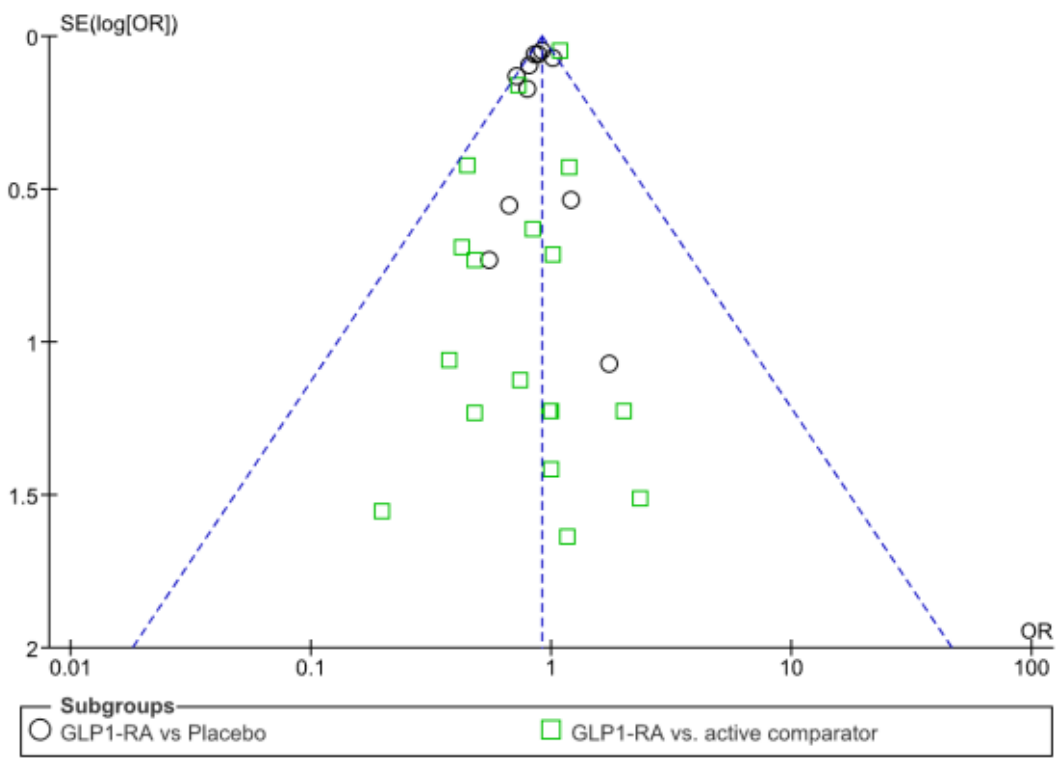

A: GLP1 RA funnel plot, MACE
Egger's test:
Intercept Int -012 (-1.90+1.28)

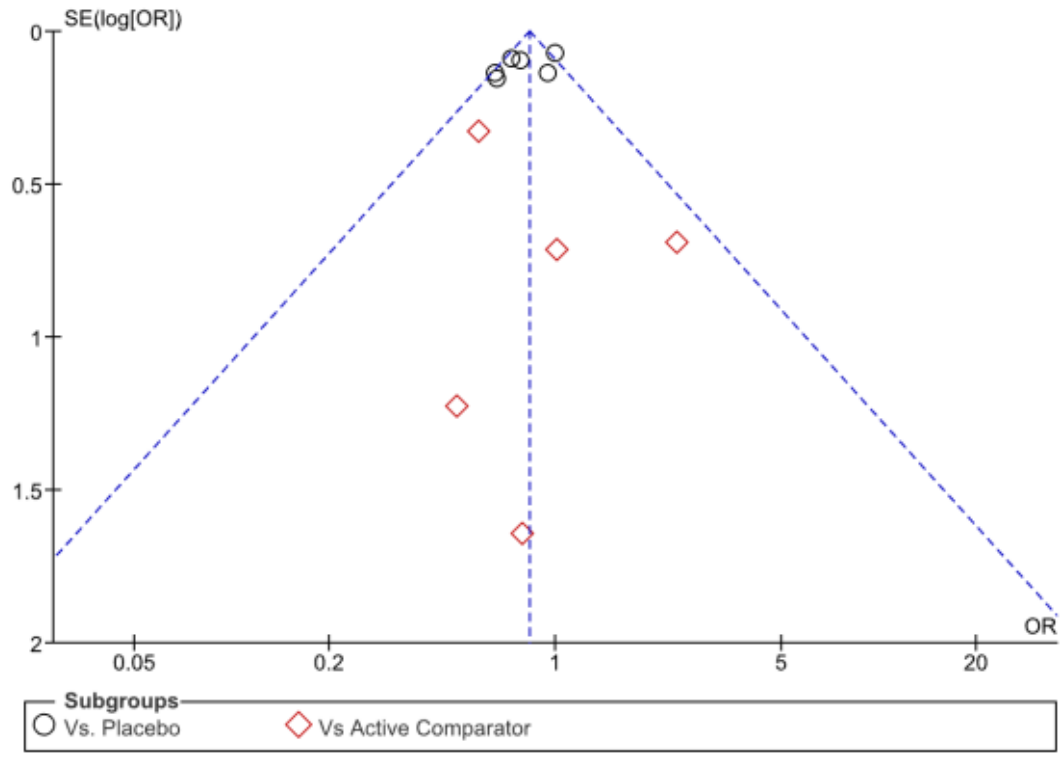

B: SGLT2i funnel plot, MACE
Egger's test:
Intercept 0.15 (-0.50+0.81), p=0.61

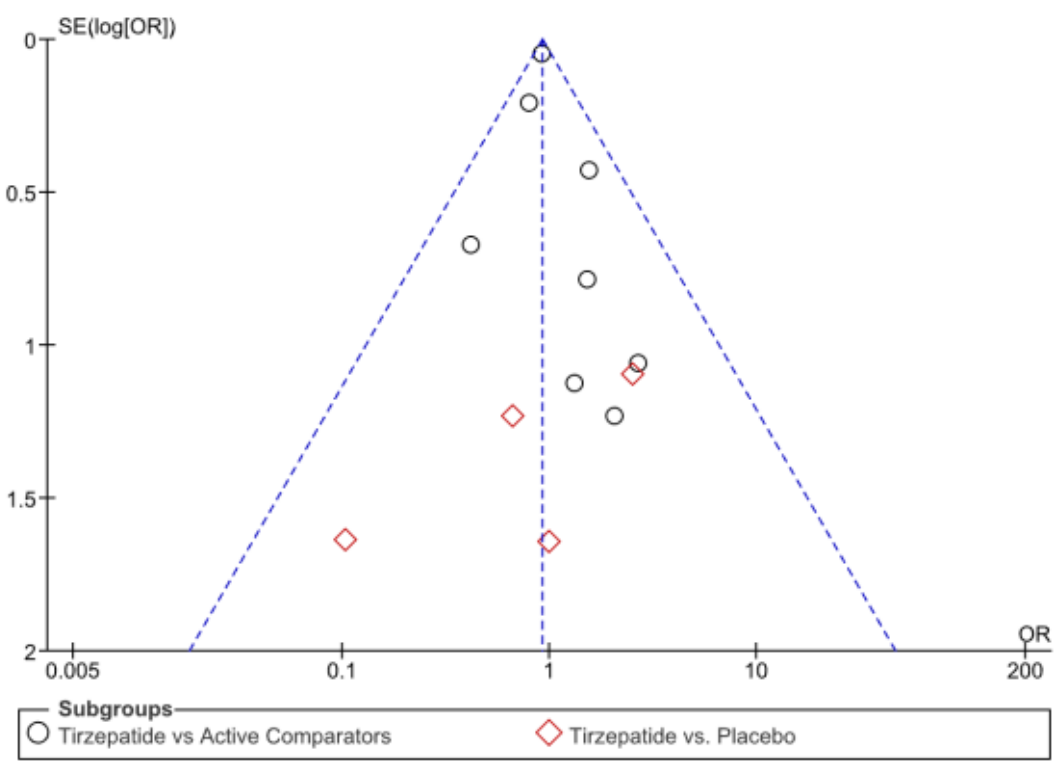

C: Tirzepatide funnel plot, MACE
Egger's test:
Intercept 0.13 (-0,30+0.92), p=0.22

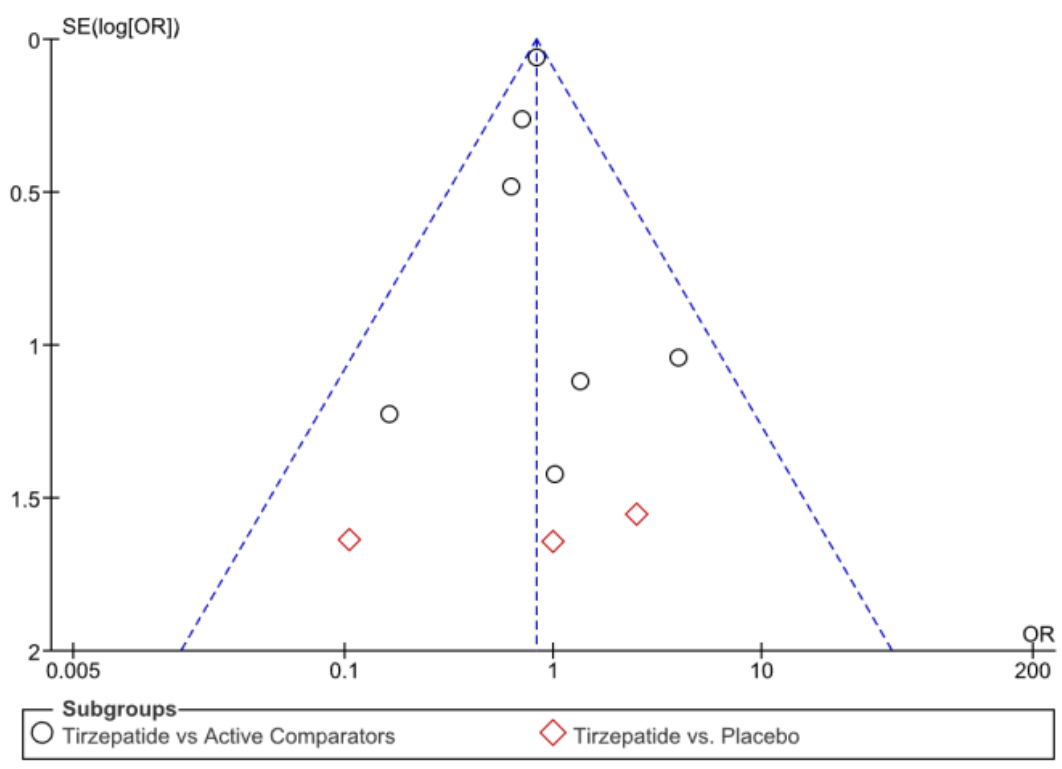

D: DPP4i funnel plot, MACE
Egger's test:
Intercept -0.12 (-0.61+0.37)

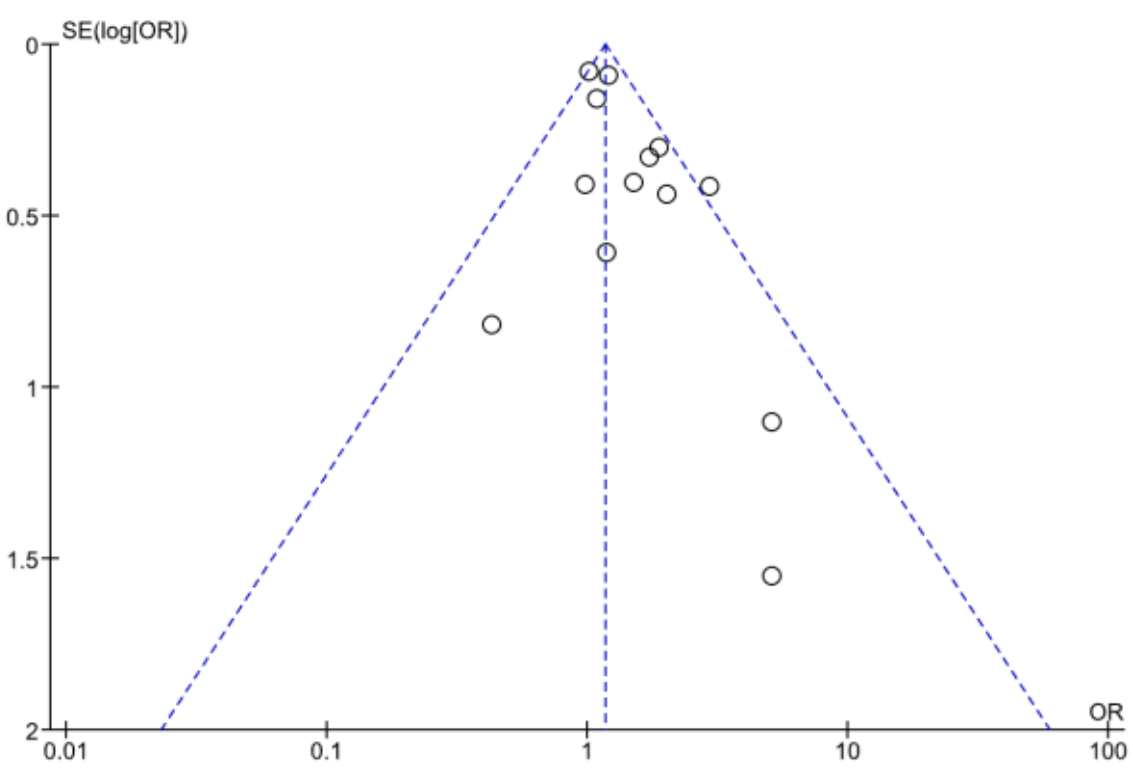

E: Sulfonylureas funnel plot, MACE
Egger's test:
Intercept 1.13 (0.26+2.021) p=0.02

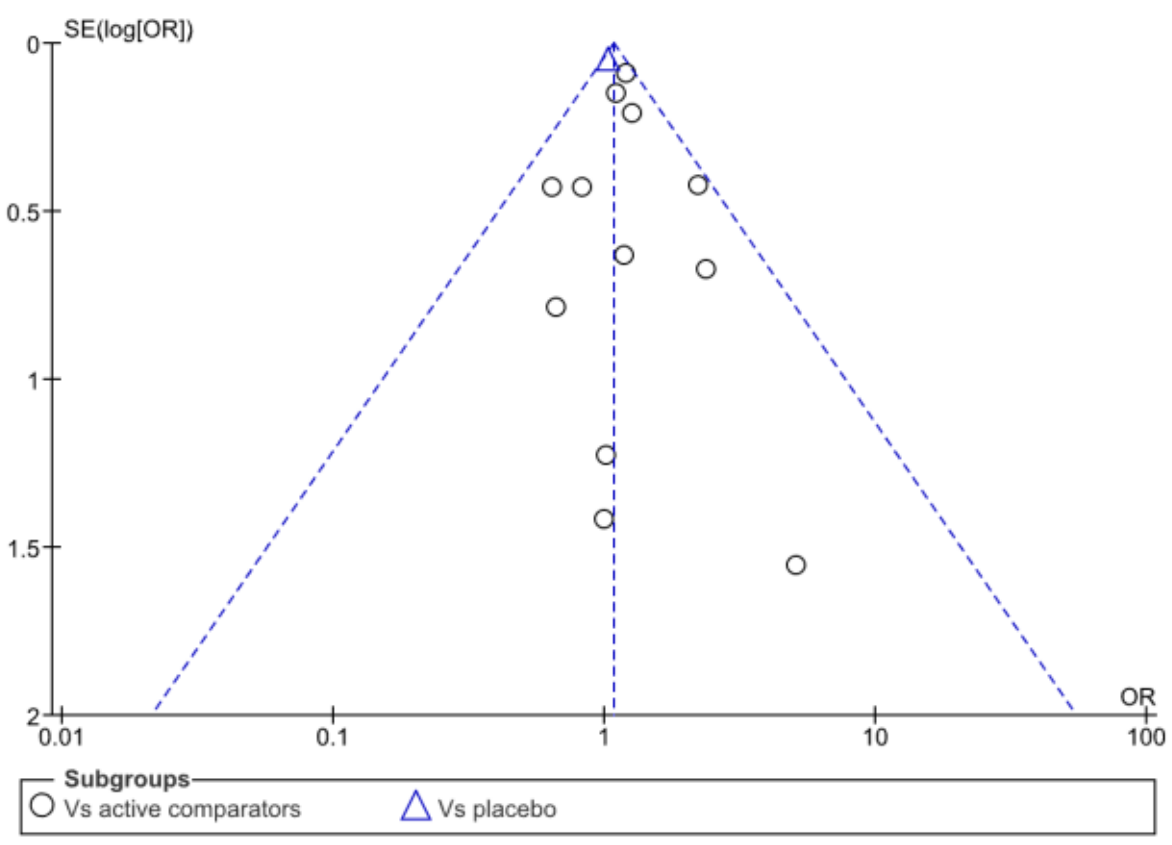

F: insulin funnel plot, MACE
Egger's test:
Intercept 0.31 (-0.43+1.05) p=0.37

Figure S4 : *Funnel plots for MACE*

Study or Subgroup	GLP1-RA Events	GLP1-RA Total	Comparator Events	Comparator Total	Weight	Odds Ratio M-H, Random, 95% CI
**3.1.1 GLP1-RA vs Placebo**						
Davies 2015	5	634	3	212	0.1%	0.55 [0.13, 2.34]
Davies 2021	12	805	5	402	0.3%	1.20 [0.42, 3.43]
Gerstein 2019	594	4949	663	4952	13.4%	0.88 [0.78, 0.99]
Holman 2017	839	7356	905	7396	16.3%	0.92 [0.84, 1.02]
Husain 2019	61	1591	76	1592	2.3%	0.80 [0.56, 1.12]
Marso 2016	608	4668	694	4672	13.5%	0.86 [0.76, 0.97]
Marso 2016 bis	108	1648	146	1649	4.0%	0.72 [0.56, 0.94]
Mc Guire 2025	579	4825	668	4825	13.3%	0.85 [0.75, 0.96]
Perkovic 2024	212	1767	254	1766	6.4%	0.81 [0.67, 0.99]
Pfeffer 2015	397	3034	389	3034	9.7%	1.02 [0.88, 1.19]
Pratley 2019	7	569	1	142	0.1%	1.76 [0.21, 14.39]
Zinman 2019	10	547	5	184	0.2%	0.67 [0.22, 1.98]
**Subtotal (95% CI)**		**32393**		**30826**	**79.6%**	**0.88 [0.84, 0.93]**
Total events	3432		3809			
Heterogeneity: Tau² = 0.00; Chi² = 9.95, df = 11 (P = 0.53); I² = 0%						
Test for overall effect: Z = 4.94 (P < 0.00001)						
**3.1.3 GLP1-RA vs. active comparator**						
Ahren 2017	2	818	1	407	0.1%	1.00 [0.09, 11.01]
Billings 2025	1	143	2	139	0.1%	0.48 [0.04, 5.38]
Blonde 2015	11	588	12	296	0.4%	0.45 [0.20, 1.04]
Buse 2020	1	253	0	98	0.0%	1.17 [0.05, 28.97]
Frias 2021	1	469	8	1410	0.1%	0.37 [0.05, 3.00]
Giorgino 2015	7	545	4	262	0.2%	0.84 [0.24, 2.89]
Gouch 2015	1	414	1	413	0.0%	1.00 [0.06, 16.00]
Green 2024	48	1262	193	3785	2.6%	0.74 [0.53, 1.02]
Inagaky 2025	1	159	4	477	0.1%	0.75 [0.08, 6.75]
Jabbour 2020	4	227	4	230	0.2%	1.01 [0.25, 4.10]
Kaku 2019	0	273	2	271	0.0%	0.20 [0.01, 4.12]
Lingvay 2019	2	394	1	394	0.1%	2.01 [0.18, 22.20]
Nicholls 2025	862	6579	801	6586	15.8%	1.09 [0.98, 1.21]
Rodbard 2019	3	411	7	410	0.2%	0.42 [0.11, 1.65]
Rosenstock 2019	3	1397	0	467	0.0%	2.35 [0.12, 45.52]
Tuttle 2018	19	384	8	192	0.4%	1.20 [0.51, 2.79]
Wang 2019 a	2	505	1	250	0.1%	0.99 [0.09, 10.97]
Weinstock 2015	5	606	3	177	0.1%	0.48 [0.11, 2.04]
**Subtotal (95% CI)**		**15427**		**16264**	**20.4%**	**1.03 [0.93, 1.13]**
Total events	973		1052			
Heterogeneity: Tau² = 0.00; Chi² = 15.13, df = 17 (P = 0.59); I² = 0%						
Test for overall effect: Z = 0.54 (P = 0.59)						
**Total (95% CI)**		**47820**		**47090**	**100.0%**	**0.90 [0.85, 0.95]**
Total events	4405		4861			
Heterogeneity: Tau² = 0.00; Chi² = 32.69, df = 29 (P = 0.29); I² = 11%						
Test for overall effect: Z = 3.72 (P = 0.0002)						
Test for subgroup differences: Chi² = 7.61, df = 1 (P = 0.006), I² = 86.9%						

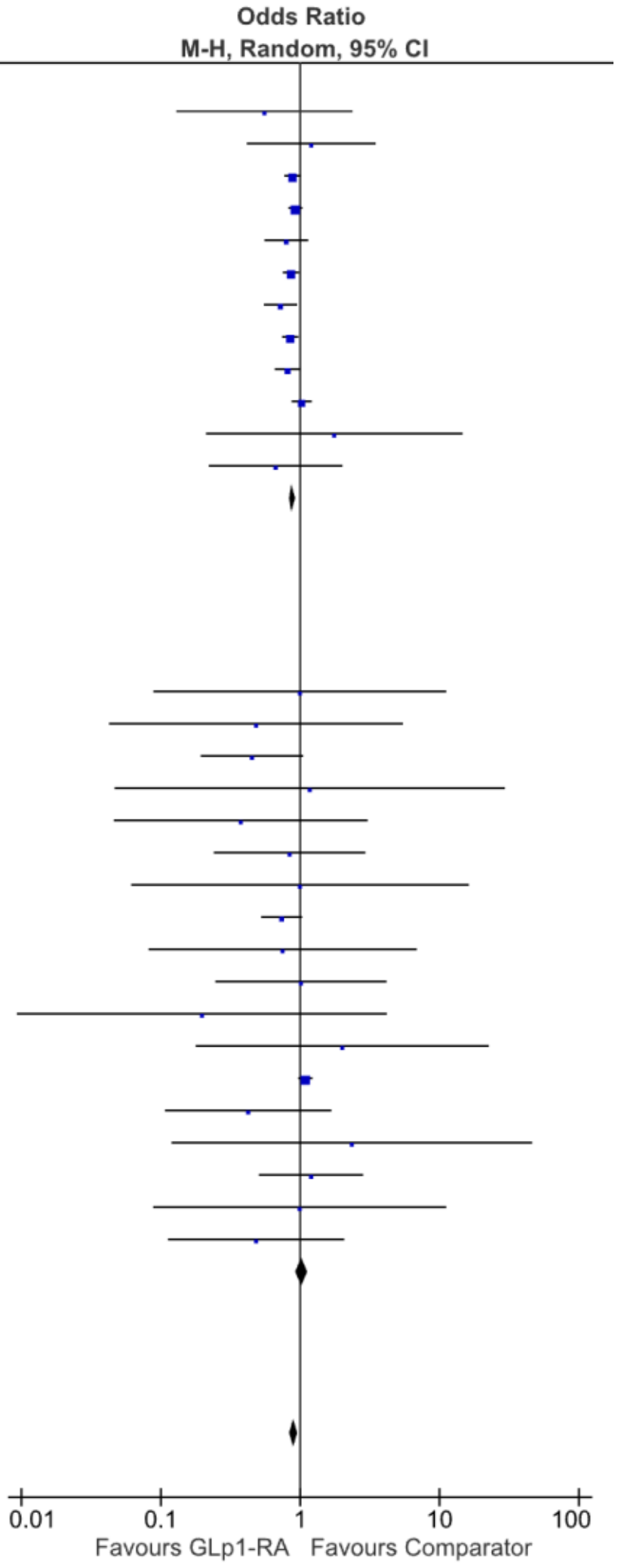

Fig. S5 difference in MACE, GLP1-RA vs comparators

Study or Subgroup	SGLt2i Events	SGLt2i Total	Control Events	Control Total	Weight	Odds Ratio M-H, Random, 95% CI
**4.2.1 Vs. Placebo**						
Cannon 2020	653	5499	327	2747	20.8%	1.00 [0.87, 1.15]
Kawamori 2018	0	0	0	0		Not estimable
Neal 2017	75	5795	84	4347	11.1%	0.67 [0.49, 0.91]
Perkovic 2019	217	2202	269	2199	17.6%	0.78 [0.65, 0.95]
Perrson 2021	113	1453	118	1450	13.1%	0.95 [0.73, 1.25]
Wiviott 2018	212	8582	286	8578	18.2%	0.73 [0.61, 0.88]
Zinman 2015	126	4687	95	2333	13.0%	0.65 [0.50, 0.85]
**Subtotal (95% CI)**		**28218**		**21654**	**93.8%**	**0.80 [0.69, 0.93]**
Total events	1396		1179			
Heterogeneity: Tau² = 0.02; Chi² = 14.58, df = 5 (P = 0.01); I² = 66%						
Test for overall effect: Z = 2.92 (P = 0.003)						
**4.2.2 Vs Active Comparator**						
Ferrannini 2013	1	215	0	56	0.2%	0.79 [0.03, 19.66]
Jabbour 2020	4	227	4	230	0.9%	1.01 [0.25, 4.10]
Lingvay 2019	1	394	2	394	0.3%	0.50 [0.05, 5.52]
Ridderstrale 2018	15	780	25	765	3.8%	0.58 [0.30, 1.11]
Rodbard 2019	7	410	3	411	1.0%	2.36 [0.61, 9.20]
**Subtotal (95% CI)**		**2026**		**1856**	**6.2%**	**0.77 [0.46, 1.30]**
Total events	28		34			
Heterogeneity: Tau² = 0.00; Chi² = 3.62, df = 4 (P = 0.46); I² = 0%						
Test for overall effect: Z = 0.98 (P = 0.32)						
**Total (95% CI)**		**30244**		**23510**	**100.0%**	**0.80 [0.70, 0.92]**
Total events	1424		1213			
Heterogeneity: Tau² = 0.02; Chi² = 18.28, df = 10 (P = 0.05); I² = 45%						
Test for overall effect: Z = 3.19 (P = 0.001)						
Test for subgroup differences: Chi² = 0.02, df = 1 (P = 0.89), I² = 0%						

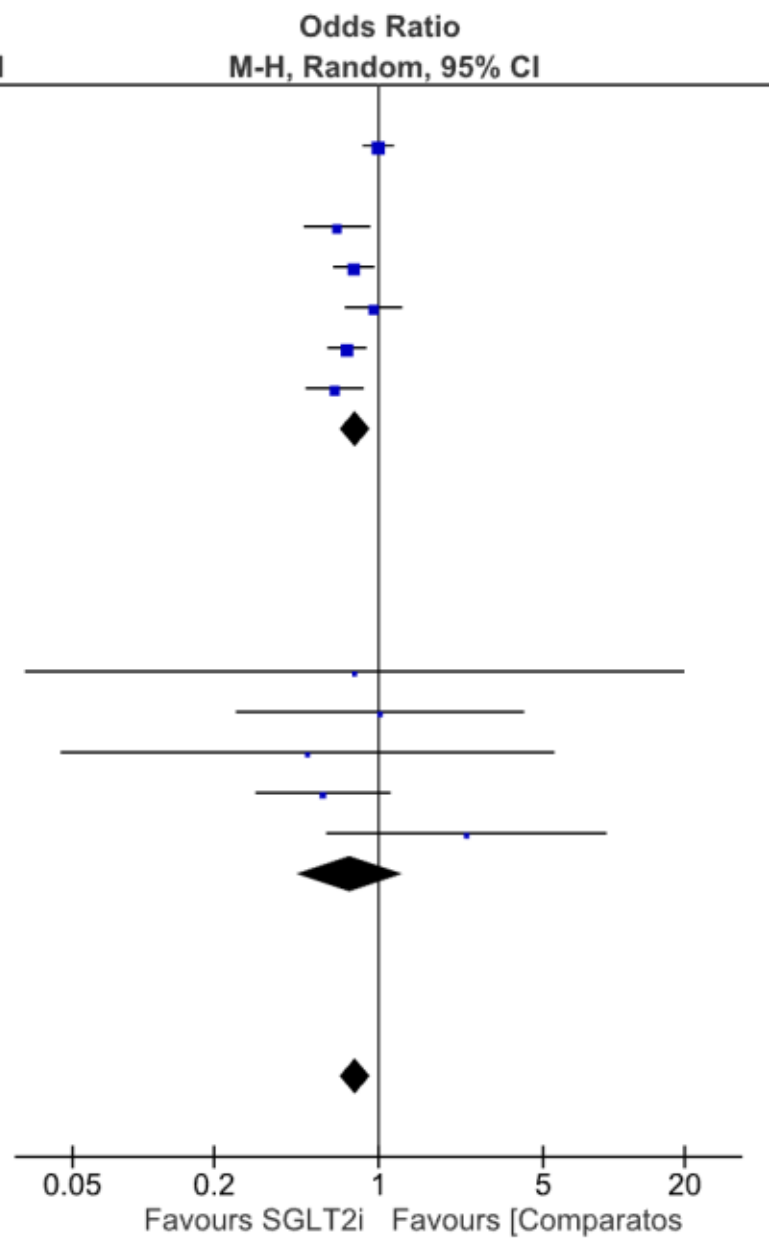

Fig. S6 difference in MACE, SGLT2i vs comparators

Study or Subgroup	Metformin Events	Metformin Total	Control Events	Control Total	Weight	Odds Ratio M-H, Random, 95% CI
Ferrannini 2013	0	56	1	215	0.8%	1.27 [0.05, 31.48]
Hong 2013	22	156	35	148	18.8%	0.53 [0.29, 0.96]
UKPDS 34-1 1998	51	342	295	1362	44.5%	0.63 [0.46, 0.88]
UKPDS 34-2 1998	55	268	107	269	35.9%	0.39 [0.27, 0.57]
**Total (95% CI)**		**822**		**1994**	**100.0%**	**0.52 [0.39, 0.69]**
Total events	128		438			

Heterogeneity: Tau² = 0.02; Chi² = 3.85, df = 3 (P = 0.28); I² = 22%
Test for overall effect: Z = 4.59 (P < 0.00001)

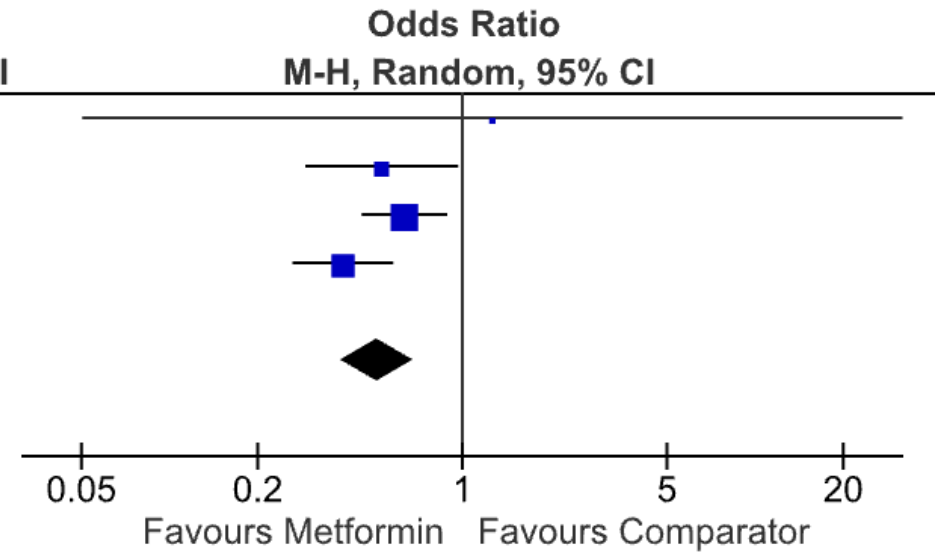

Fig.S7 difference in MACE, metformin vs comparators

Study or Subgroup	Pioglitazone Events	Pioglitazone Total	Control Events	Control Total	Weight	Odds Ratio M-H, Fixed, 95% CI
**8.2.1 Pioglitazone vs Placebo**						
Dormandy 2005	301	2605	358	2633	72.4%	0.83 [0.70, 0.98]
Home 2015	6	277	3	115	1.0%	0.83 [0.20, 3.36]
Yoshii 2014	9	234	10	247	2.2%	0.95 [0.38, 2.38]
**Subtotal (95% CI)**		**3116**		**2995**	**75.5%**	**0.83 [0.71, 0.98]**
Total events	316		371			
Heterogeneity: Chi² = 0.08, df = 2 (P = 0.96); I² = 0%						
Test for overall effect: Z = 2.23 (P = 0.03)						
**8.2.2 Pioglitazone vs DPP4i**						
Bolli 2009	2	281	3	295	0.7%	0.70 [0.12, 4.21]
Henry 2014	2	693	3	231	1.0%	0.22 [0.04, 1.32]
Nauck 2016	2	135	2	409	0.2%	3.06 [0.43, 21.94]
**Subtotal (95% CI)**		**1109**		**935**	**1.9%**	**0.72 [0.25, 2.04]**
Total events	6		8			
Heterogeneity: Chi² = 3.75, df = 2 (P = 0.15); I² = 47%						
Test for overall effect: Z = 0.62 (P = 0.53)						
**8.2.3 Pioglitazone vs SU**						
Giles 2010	13	149	13	151	2.7%	1.01 [0.45, 2.27]
Mazzone 2006	0	230	2	228	0.6%	0.20 [0.01, 4.12]
Nissen 2008	5	270	6	273	1.3%	0.84 [0.25, 2.78]
Vaccaro 2017	74	1493	83	1535	17.9%	0.91 [0.66, 1.26]
**Subtotal (95% CI)**		**2142**		**2187**	**22.5%**	**0.90 [0.68, 1.20]**
Total events	92		104			
Heterogeneity: Chi² = 1.06, df = 3 (P = 0.79); I² = 0%						
Test for overall effect: Z = 0.70 (P = 0.48)						
**Total (95% CI)**		**6367**		**6117**	**100.0%**	**0.85 [0.74, 0.97]**
Total events	414		483			

Heterogeneity: Chi² = 5.25, df = 9 (P = 0.81); I² = 0%
Test for overall effect: Z = 2.35 (P = 0.02)
Test for subgroup differences: Chi² = 0.32, df = 2 (P = 0.85), I² = 0%

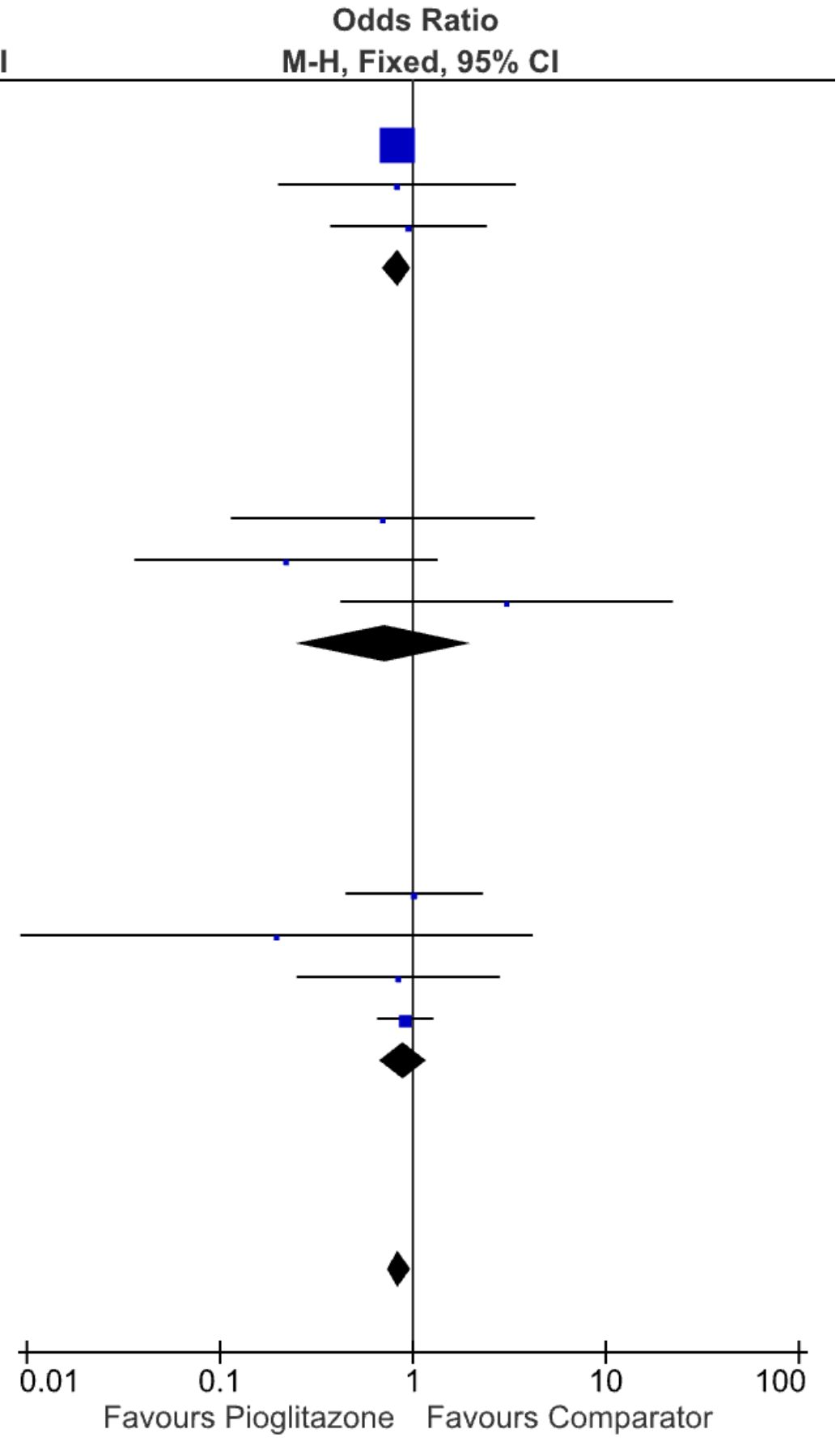

Fig. S8 difference in MACE, pioglitazone vs comparators

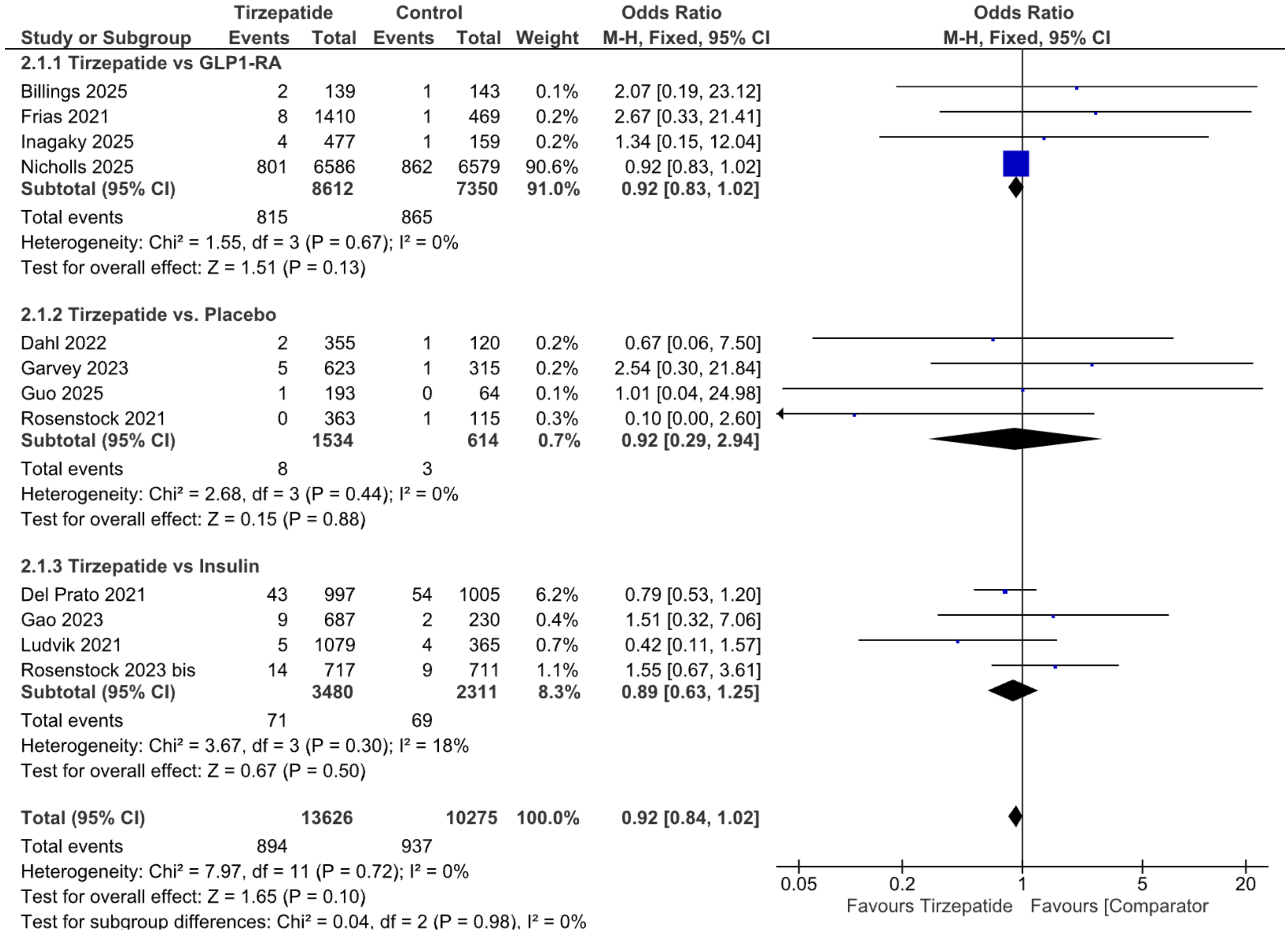

Fig. S9 difference in MACE, Tirzepatide vs comparators

Study or Subgroup	DPP4i Events	DPP4i Total	Control Events	Control Total	Weight	Odds Ratio M-H, Fixed, 95% CI
Ahren 2014	2	302	6	408	0.2%	0.45 [0.09, 2.23]
Ahren 2017	1	407	2	818	0.1%	1.00 [0.09, 11.12]
Arjona-Ferreira 2013	1	211	5	212	0.2%	0.20 [0.02, 1.70]
Bolli 2009	3	295	2	281	0.1%	1.43 [0.24, 8.64]
Buse 2020	0	98	1	253	0.0%	0.85 [0.03, 21.15]
Cioffi 2021	0	93	1	91	0.1%	0.32 [0.01, 8.02]
Del Prato 2014	14	1665	11	874	0.6%	0.67 [0.30, 1.47]
Foley 2009	8	546	16	546	0.7%	0.49 [0.21, 1.16]
Gallwitz 2012	11	776	23	775	0.9%	0.47 [0.23, 0.97]
Green 2015	745	7332	746	7339	27.7%	1.00 [0.90, 1.11]
Green 2024	69	1268	172	3790	3.4%	1.21 [0.91, 1.61]
Henry 2014	3	231	2	693	0.0%	4.55 [0.75, 27.38]
Kaku 2019 bis	0	213	1	214	0.1%	0.33 [0.01, 8.23]
Li 2020	1	75	0	74	0.0%	3.00 [0.12, 74.84]
Matthei 2016	0	153	1	162	0.1%	0.35 [0.01, 8.67]
Matthews 2019	21	998	29	1003	1.2%	0.72 [0.41, 1.27]
Mc Murray 2018	13	128	7	126	0.3%	1.92 [0.74, 4.99]
Mita 2016	0	172	2	169	0.1%	0.19 [0.01, 4.08]
Mita 2016 bis	4	142	2	140	0.1%	2.00 [0.36, 11.10]
Nauck 2016	2	135	2	409	0.0%	3.06 [0.43, 21.94]
Oyama 2016	4	222	3	220	0.1%	1.33 [0.29, 6.00]
Rosenstock 2019	0	467	3	1397	0.1%	0.43 [0.02, 8.26]
Rosenstock 2019 bis	434	3494	420	3485	15.2%	1.04 [0.90, 1.19]
Rosenstock 2019 ter	356	3023	362	3010	13.2%	0.98 [0.84, 1.14]
Scirica 2013	613	8280	609	8212	23.4%	1.00 [0.89, 1.12]
Weinstock 2015	3	177	5	606	0.1%	2.07 [0.49, 8.76]
White 2013	315	2701	316	2679	11.6%	0.99 [0.84, 1.17]
Yki-jarvinen 2013	18	631	11	630	0.4%	1.65 [0.77, 3.53]
**Total (95% CI)**		**34235**		**38616**	**100.0%**	**1.00 [0.94, 1.06]**
Total events	2641		2760			

Heterogeneity: Chi² = 26.83, df = 27 (P = 0.47); I² = 0%
Test for overall effect: Z = 0.04 (P = 0.97)

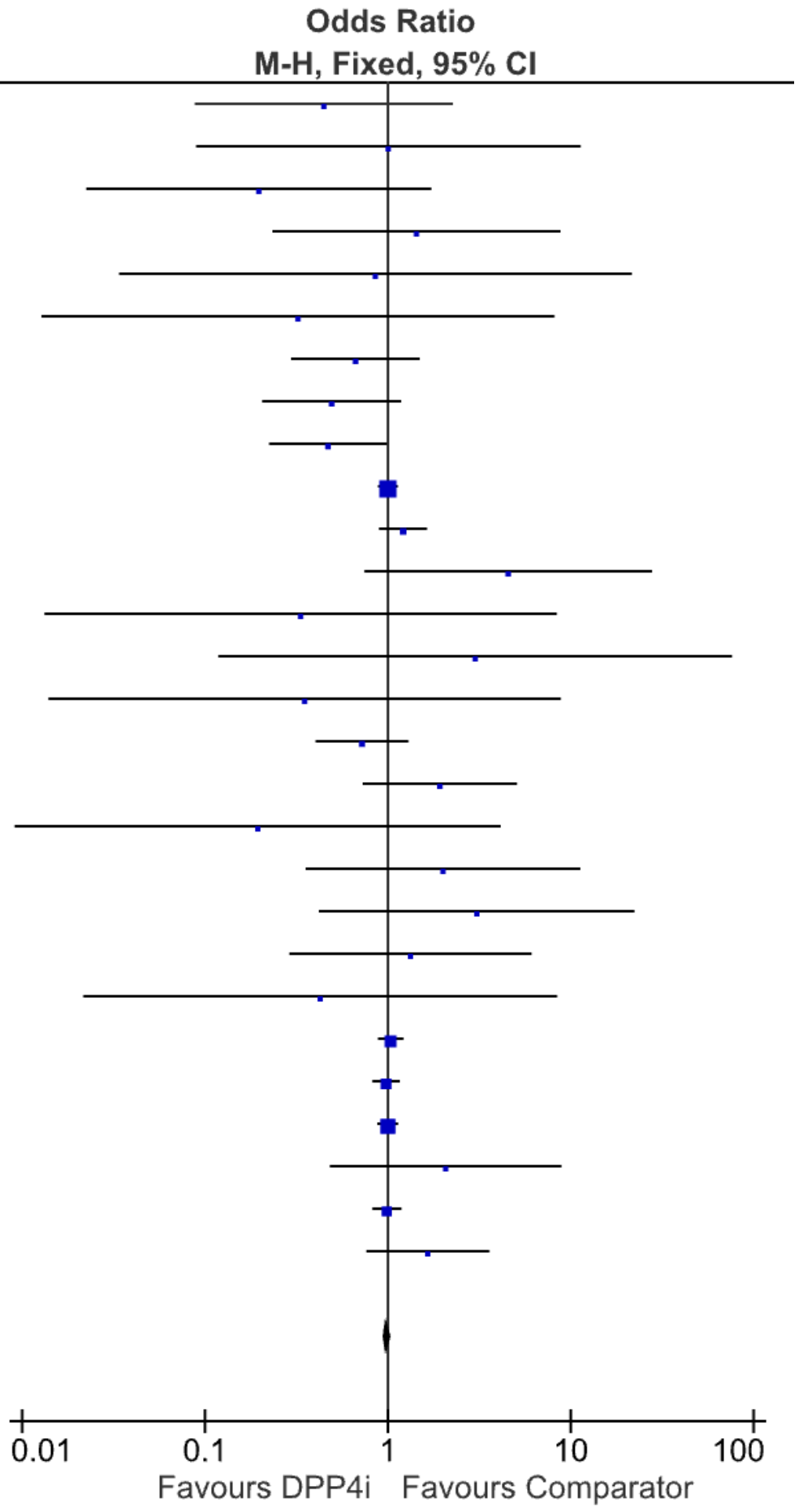

Fig. S10 difference in MACE, DPP4 inhibitors vs comparators

Study or Subgroup	Insulin Events	Insulin Total	Control Events	Control Total	Weight	Odds Ratio M-H, Fixed, 95% CI
Blonde 2015	12	296	11	588	0.6%	2.22 [0.97, 5.08]
Del Prato 2021	54	1005	43	997	3.2%	1.26 [0.84, 1.90]
Gao 2023	2	230	9	687	0.4%	0.66 [0.14, 3.08]
Gerstein 2012	1041	6264	1013	6273	67.0%	1.03 [0.94, 1.14]
Giorgino 2015	4	262	7	545	0.4%	1.19 [0.35, 4.11]
Gouch 2015	1	413	1	414	0.1%	1.00 [0.06, 16.08]
Green 2024	65	1263	176	3784	6.6%	1.11 [0.83, 1.49]
Kaku 2019	2	271	0	273	0.0%	5.07 [0.24, 106.19]
Ludvik 2021	4	365	5	1079	0.2%	2.38 [0.64, 8.91]
Rosenstock 2023 bis	9	711	14	717	1.1%	0.64 [0.28, 1.50]
Tuttle 2018	8	192	19	384	1.0%	0.84 [0.36, 1.94]
UKPDS 33 1998	211	911	531	2130	19.4%	0.91 [0.76, 1.09]
Wang 2019 a	1	250	2	505	0.1%	1.01 [0.09, 11.19]
**Total (95% CI)**		**12433**		**18376**	**100.0%**	**1.03 [0.95, 1.11]**
Total events	1414		1831			

Heterogeneity: Chi² = 10.71, df = 12 (P = 0.55); I² = 0%
Test for overall effect: Z = 0.66 (P = 0.51)

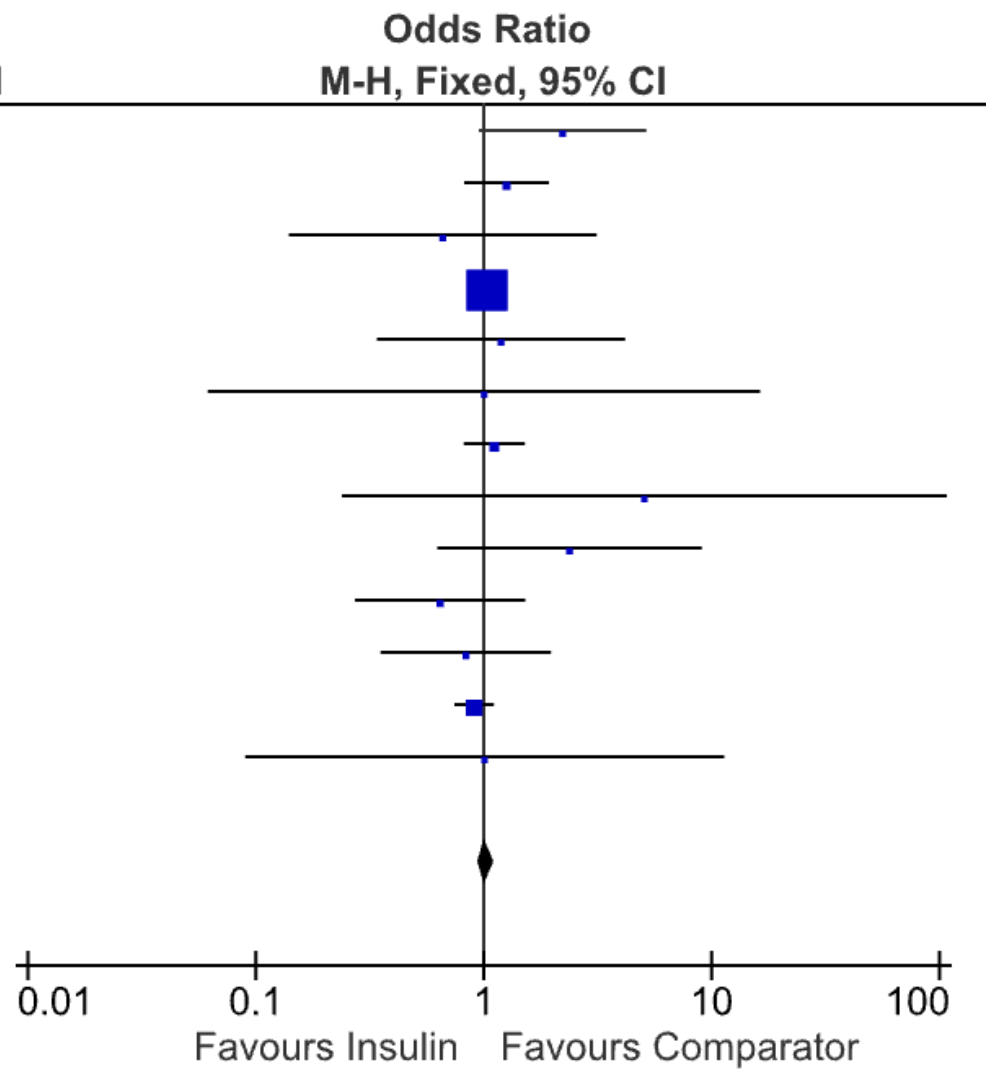

Fig. S11 difference in MACE, insulin vs comparators

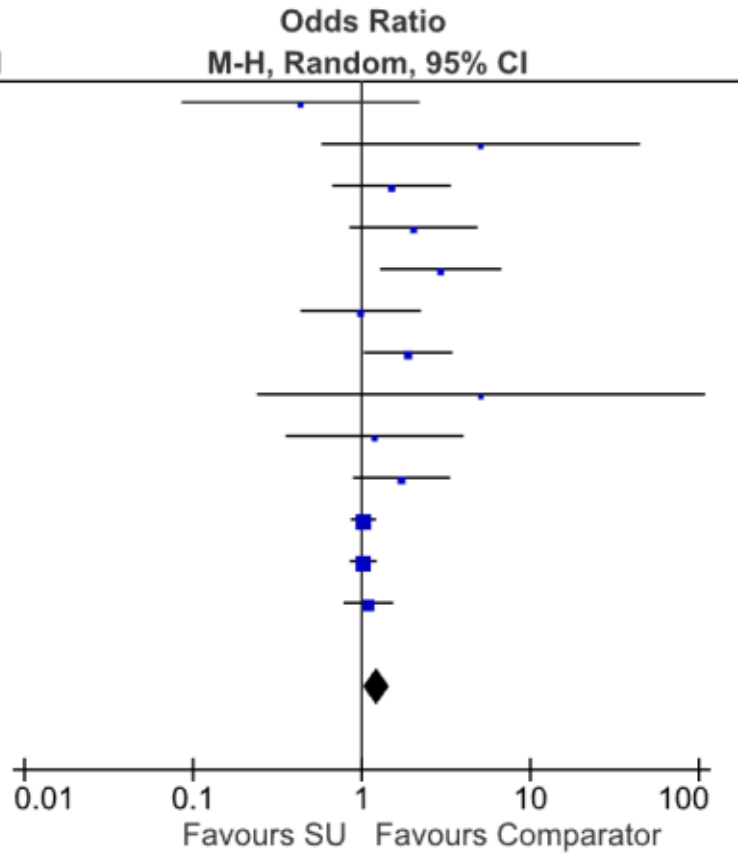

Study or Subgroup	SU Events	SU Total	Control Events	Control Total	Weight	Odds Ratio M-H, Random, 95% CI
Ahren 2014	2	307	6	403	1.2%	0.43 [0.09, 2.16]
Arjona-Ferreira 2013	5	212	1	211	0.7%	5.07 [0.59, 43.79]
Del Prato 2014	11	874	14	1665	4.4%	1.50 [0.68, 3.33]
Foley 2009	16	546	8	546	3.9%	2.03 [0.86, 4.78]
Gallwitz 2012	23	775	8	776	4.3%	2.94 [1.31, 6.61]
Giles 2010	13	151	13	149	4.3%	0.99 [0.44, 2.20]
Hong 2013	35	148	22	156	7.2%	1.89 [1.05, 3.40]
Mazzone 2006	2	228	0	230	0.3%	5.09 [0.24, 106.57]
Nissen 2008	6	273	5	270	2.1%	1.19 [0.36, 3.95]
Ridderstrale 2018	25	765	15	780	6.2%	1.72 [0.90, 3.29]
Rosenstock 2019 ter	362	3010	356	3023	25.2%	1.02 [0.88, 1.20]
UKPDS 33 1998	299	1234	453	1897	24.5%	1.02 [0.86, 1.21]
Vaccaro 2017	83	1535	74	1493	15.6%	1.10 [0.79, 1.51]
**Total (95% CI)**		**10058**		**11599**	**100.0%**	**1.23 [1.03, 1.47]**
Total events	882		975			

Heterogeneity: Tau² = 0.03; Chi² = 19.15, df = 12 (P = 0.09); I² = 37%
Test for overall effect: Z = 2.24 (P = 0.03)

Fig. S12 difference in MACE, sulfonylureas vs comparators

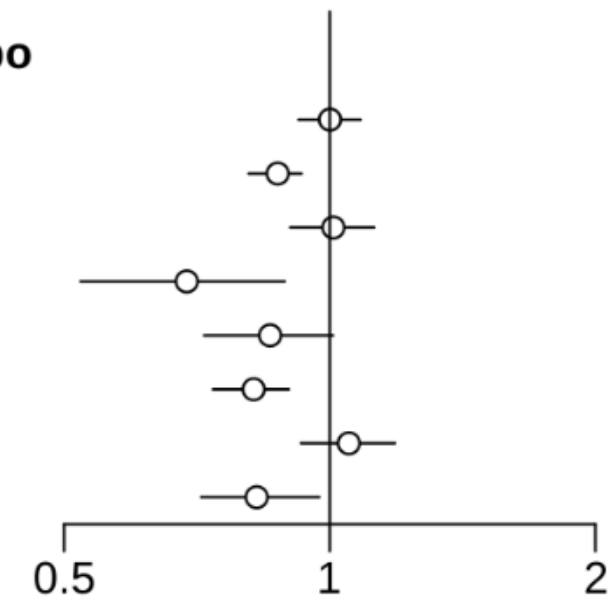

Compared with Placebo	Odds Ratio (95% CrI)
DPP4i	1.00 (0.922, 1.08)
GLP_1RA	0.873 (0.810, 0.929)
Insulin	1.01 (0.902, 1.12)
Metformin	0.689 (0.522, 0.889)
Pioglitazone	0.856 (0.720, 1.01)
SGLT2i	0.820 (0.737, 0.899)
SU	1.05 (0.927, 1.19)
Tirzepatide	0.826 (0.715, 0.973)

Fig. S13, Bayesian Meta-analysis, forest plot: difference in the incidence of MACE between Diabetes drug classes and placebo

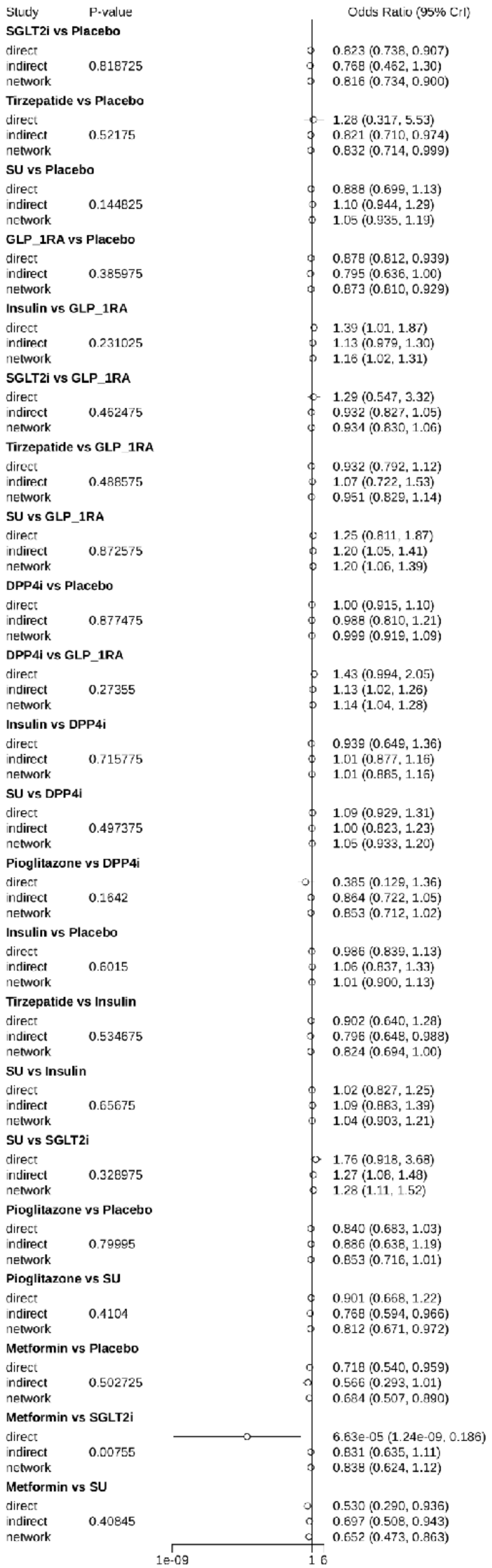

Fig. S14 Nodesplit analysis (inconsistency test with notesplitting model for all studies)

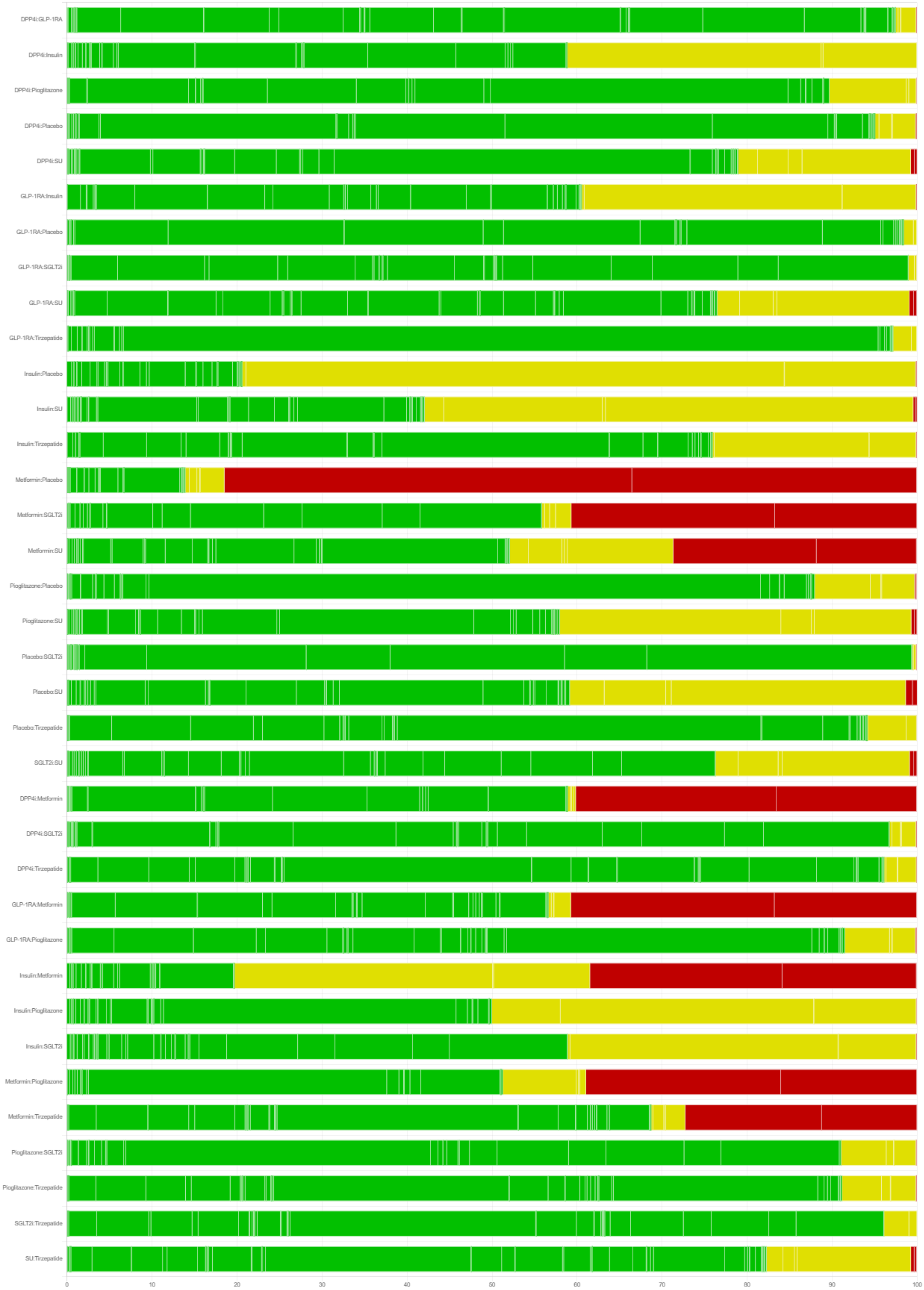

Fig. S15 MACE within study bias

Comparison	N	Within-study bias	Reporting bias	Indirectness	Imprecision	Heterogeneity	Incoherence	Confidence rating	Reason(s) for downgrading
DPP4i vs GLP-1RA	5	No concerns	Low risk	No concerns	No concerns	No concerns	No concerns	High	
DPP4i vs Insulin	1	No concerns	Low risk	No concerns	No concerns	No concerns	No concerns	High	
DPP4i vs Pioglitazone	3	No concerns	Low risk	No concerns	No concerns	Some concerns	No concerns	Moderate	Heterogeneity
DPP4i vs Placebo	15	No concerns	Low risk	No concerns	No concerns	No concerns	No concerns	High	
DPP4i vs SU	7	No concerns	Low risk	No concerns	No concerns	No concerns	No concerns	High	
GLP-1RA vs Insulin	7	No concerns	Low risk	No concerns	No concerns	No concerns	No concerns	High	
GLP-1RA vs Placebo	14	No concerns	Low risk	No concerns	No concerns	No concerns	No concerns	High	
GLP-1RA vs SGLT2i	3	No concerns	Low risk	No concerns	No concerns	Some concerns	No concerns	Moderate	Heterogeneity
GLP-1RA vs SU	1	No concerns	Low risk	No concerns	No concerns	No concerns	No concerns	High	
GLP-1RA vs Tirzepatide	4	No concerns	Low risk	No concerns	No concerns	Some concerns	No concerns	Moderate	Heterogeneity
Insulin vs Placebo	2	Some concerns	Low risk	No concerns	No concerns	No concerns	No concerns	Moderate	Within-study bias
Insulin vs SU	2	Some concerns	Some concerns	No concerns	No concerns	No concerns	No concerns	Low	Within-study bias Reporting bias
Insulin vs Tirzepatide	4	No concerns	Low risk	No concerns	No concerns	No concerns	No concerns	High	
Metformin vs Placebo	2	Major concerns	Some concerns	No concerns	No concerns	No concerns	No concerns	Low	Reporting bias
Metformin vs SGLT2i	1	Some concerns	Low risk	No concerns	Some concerns	No concerns	No concerns	Moderate	Within-study bias Imprecision
Metformin vs SU	1	Some concerns	Some concerns	No concerns	No concerns	No concerns	No concerns	Low	Within-study bias Reporting bias
Pioglitazone vs Placebo	3	No concerns	Low risk	No concerns	No concerns	No concerns	No concerns	High	
Pioglitazone vs SU	4	No concerns	Low risk	No concerns	No concerns	No concerns	No concerns	High	
SGLT2i vs Placebo	8	No concerns	Low risk	No concerns	No concerns	No concerns	No concerns	High	
SU vs Placebo	2	No concerns	Low risk	No concerns	No concerns	No concerns	Major concerns	Low	Incoherence
Tirzepatide vs Placebo	4	No concerns	Low risk	No concerns	No concerns	No concerns	No concerns	High	
SGLT2i vs SU	1	No concerns	Low risk	No concerns	No concerns	No concerns	No concerns	High	
DPP4i vs Metformin		Some concerns	Low risk	No concerns	No concerns	No concerns	No concerns	Moderate	Within-study bias
DPP4i vs SGLT2i		No concerns	Low risk	No concerns	No concerns	No concerns	No concerns	High	
DPP4i vs Tirzepatide		No concerns	Low risk	No concerns	No concerns	No concerns	No concerns	High	
GLP-1RA vs Metformin		Some concerns	Low risk	No concerns	Some concerns	No concerns	No concerns	Low	Within-study bias Imprecision
GLP-1RA vs Pioglitazone		No concerns	Low risk	No concerns	Some concerns	No concerns	No concerns	Moderate	Imprecision
Insulin vs Metformin		Some concerns	Low risk	No concerns	No concerns	No concerns	No concerns	Moderate	Within-study bias
Insulin vs Pioglitazone		No concerns	Low risk	No concerns	No concerns	Some concerns	No concerns	Moderate	Heterogeneity
Insulin vs SGLT2i		No concerns	Low risk	No concerns	No concerns	No concerns	No concerns	High	
Metformin vs Pioglitazone		Some concerns	Low risk	No concerns	Some concerns	No concerns	No concerns	Low	Within-study bias Imprecision
Metformin vs Tirzepatide		Some concerns	Low risk	No concerns	Some concerns	No concerns	No concerns	Low	Within-study bias Imprecision
Pioglitazone vs SGLT2i		No concerns	Low risk	No concerns	Some concerns	No concerns	No concerns	Moderate	Imprecision
Pioglitazone vs Tirzepatide		No concerns	Low risk	No concerns	Some concerns	No concerns	No concerns	Moderate	Imprecision
SGLT2i vs Tirzepatide		No concerns	Low risk	No concerns	No concerns	No concerns	No concerns	High	
SU vs Tirzepatide		No concerns	Low risk	No concerns	No concerns	No concerns	No concerns	High	

Fig. S16 MACE confidence rating

SECONDARY OUTCOME: ALL-CAUSE MORTALITY Traditional meta-analyses (by class)

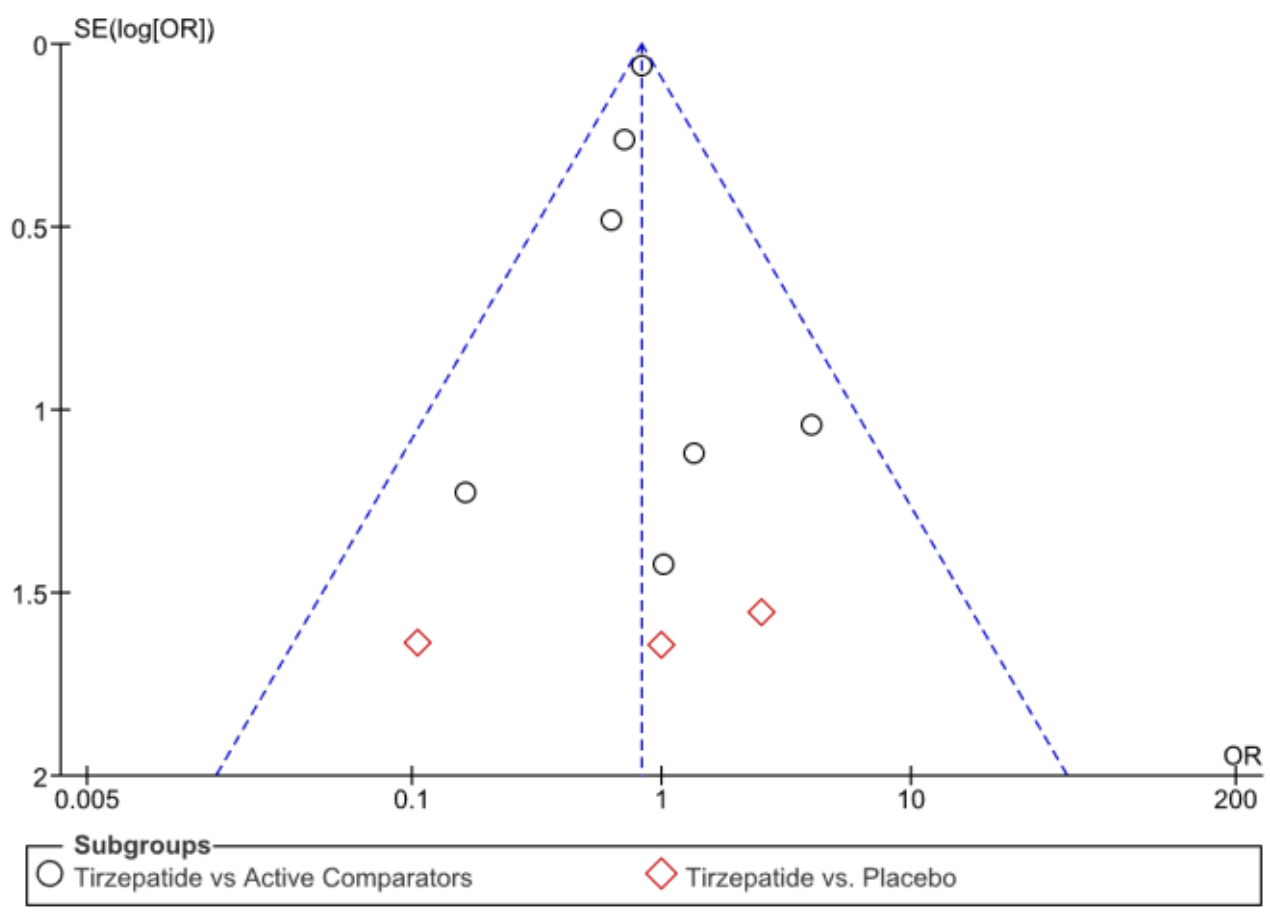

A: GLP1 RA funnel plot, mortality

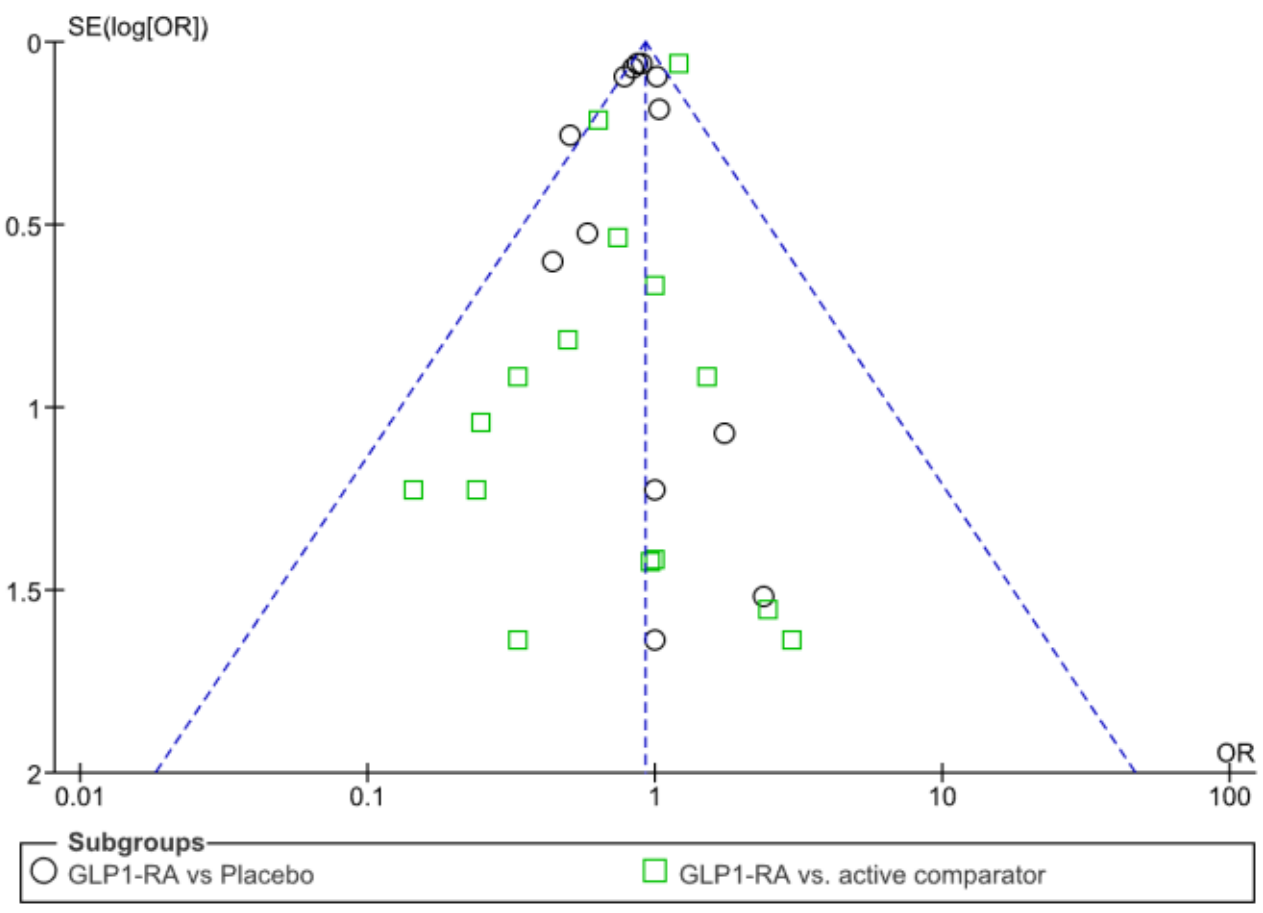

B: SGLT2i funnel plot, mortality

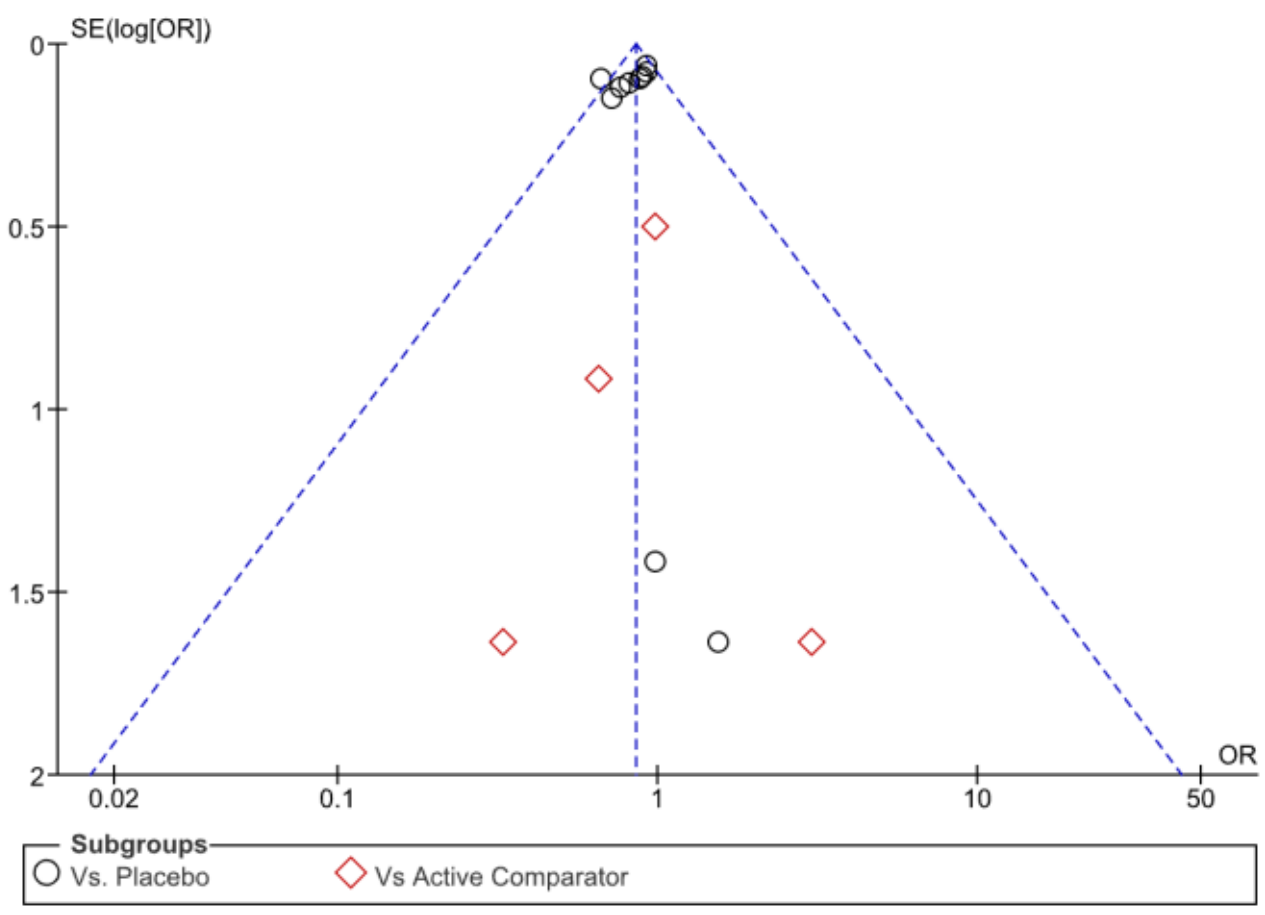

C: Tirzepatide funnel plot, mortality

-

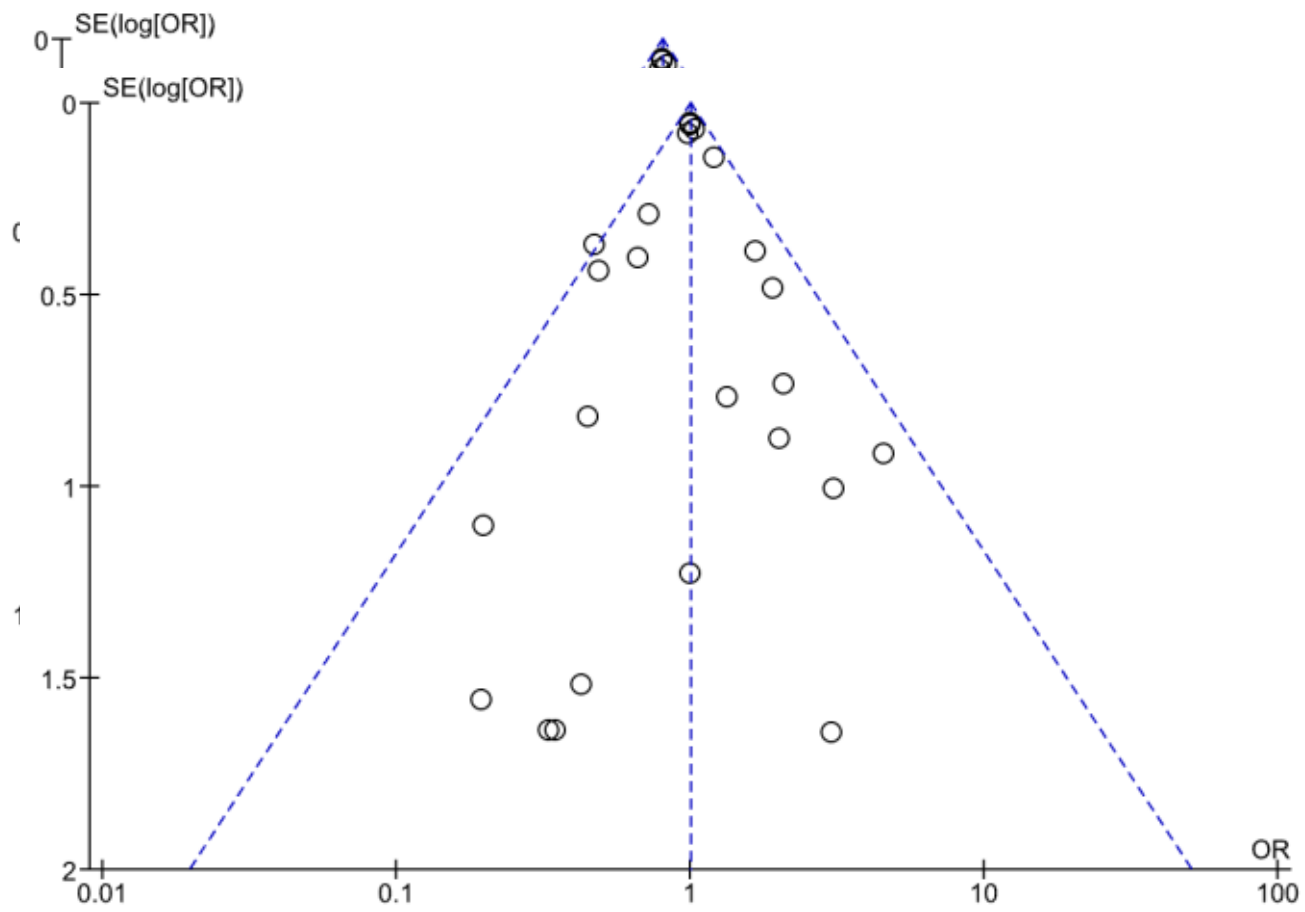

D: DPP4i funnel plot, mortality

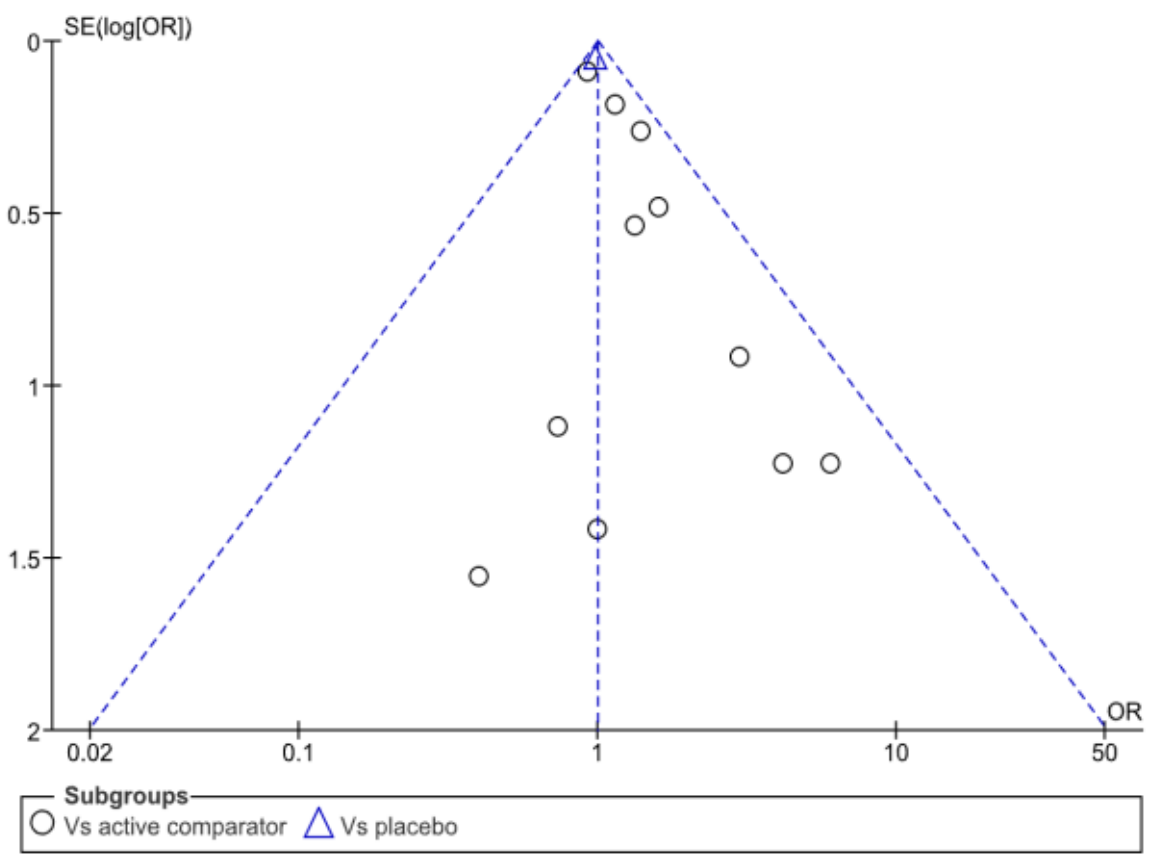

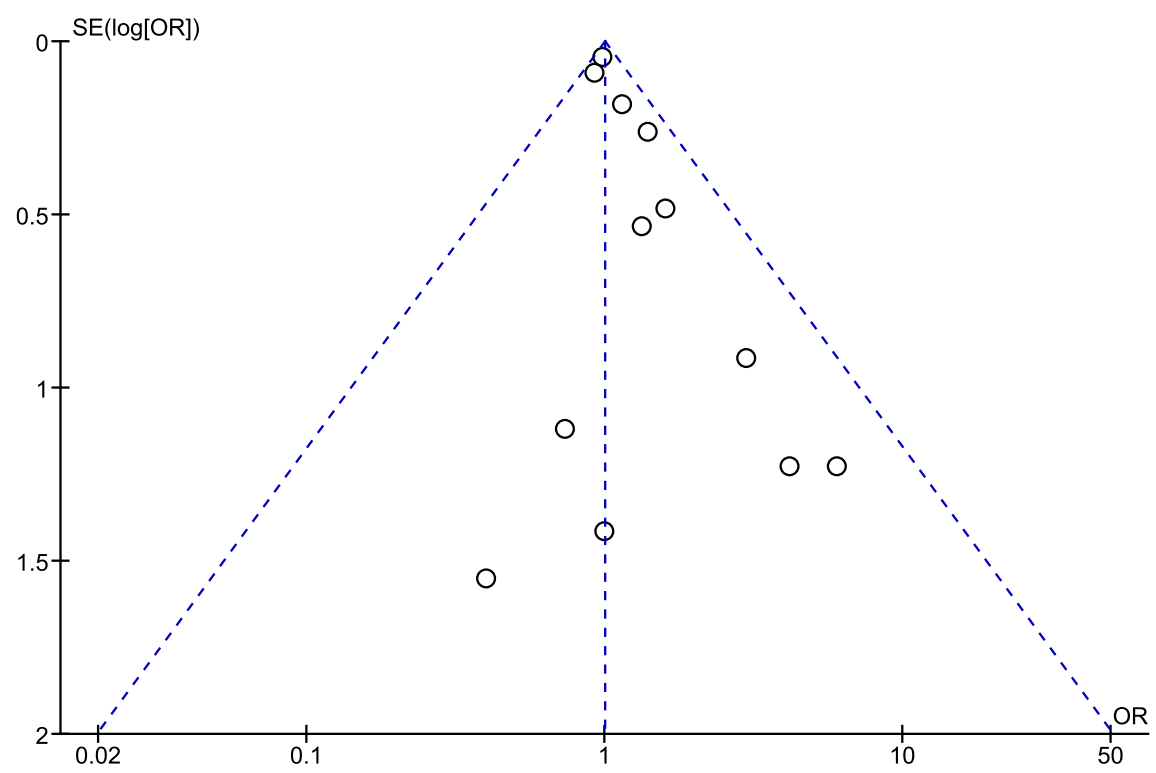

E: Sulfonylureas funnel plot, mortality

F: insulin funnel plot, mortality

Fig. S17 Overall mortality: funnel plots

Study or Subgroup	Tirzepatide Events	Tirzepatide Total	Control Events	Control Total	Weight	Odds Ratio M-H, Fixed, 95% CI
**2.2.1 Tirzepatide vs Active Comparators**						
Billings 2025	1	139	1	143	0.1%	1.03 [0.06, 16.61]
Del Prato 2021	25	997	35	1005	5.1%	0.71 [0.42, 1.20]
Frias 2021	12	1410	1	469	0.2%	4.02 [0.52, 30.98]
Gao 2023	1	687	2	230	0.4%	0.17 [0.01, 1.84]
Ludvik 2021	4	1079	1	365	0.2%	1.35 [0.15, 12.16]
Nicholls 2025	566	6586	669	6579	91.7%	0.83 [0.74, 0.93]
Rosenstock 2023 bis	7	717	11	711	1.6%	0.63 [0.24, 1.63]
**Subtotal (95% CI)**		**11615**		**9502**	**99.4%**	**0.83 [0.74, 0.93]**
Total events	616		720			
Heterogeneity: Chi² = 4.87, df = 6 (P = 0.56); I² = 0%						
Test for overall effect: Z = 3.30 (P = 0.0010)						
**2.2.2 Tirzepatide vs. Placebo**						
Garvey 2023	2	623	0	315	0.1%	2.54 [0.12, 53.03]
Guo 2025	1	193	0	64	0.1%	1.01 [0.04, 24.98]
Rosenstock 2021	0	363	1	115	0.3%	0.10 [0.00, 2.60]
**Subtotal (95% CI)**		**1179**		**494**	**0.6%**	**0.72 [0.16, 3.32]**
Total events	3		1			
Heterogeneity: Chi² = 2.09, df = 2 (P = 0.35); I² = 4%						
Test for overall effect: Z = 0.41 (P = 0.68)						
**Total (95% CI)**		**12794**		**9996**	**100.0%**	**0.83 [0.74, 0.92]**
Total events	619		721			
Heterogeneity: Chi² = 6.99, df = 9 (P = 0.64); I² = 0%						
Test for overall effect: Z = 3.32 (P = 0.0009)						
Test for subgroup differences: Chi² = 0.03, df = 1 (P = 0.87), I² = 0%						

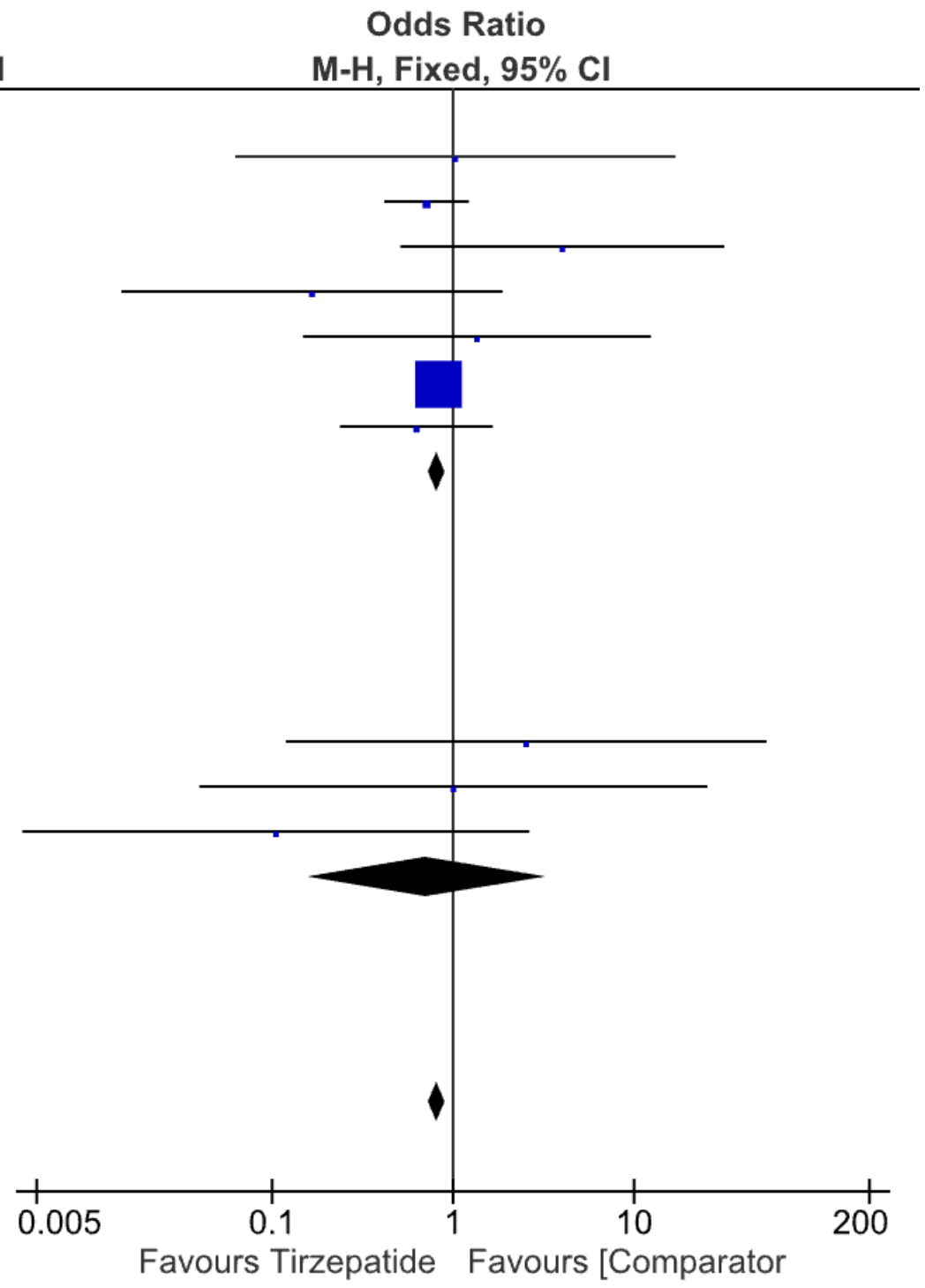

Fig. S18 Overall mortality: Tirzepatide vs comparators

Study or Subgroup	SGLt2i Events	SGLt2i Total	Control Events	Control Total	Weight	Odds Ratio M-H, Fixed, 95% CI
**4.3.1 Vs. Placebo**						
Cannon 2020	473	5499	254	2747	15.4%	0.92 [0.79, 1.08]
Inzucchi 2022	265	1575	291	1575	12.1%	0.89 [0.74, 1.07]
Jabbour 2018	1	233	1	231	0.0%	0.99 [0.06, 15.94]
Kawamori 2018	1	182	0	93	0.0%	1.55 [0.06, 38.31]
Neal 2017	228	5795	193	4347	10.6%	0.88 [0.72, 1.07]
Perkovic 2019	168	2202	201	2199	9.3%	0.82 [0.66, 1.02]
Perrson 2021	84	1453	113	1450	5.3%	0.73 [0.54, 0.97]
Petrie 2021	143	1075	178	1064	7.7%	0.76 [0.60, 0.97]
Wiviott 2018	529	8582	570	8578	26.7%	0.92 [0.82, 1.04]
Zinman 2015	269	4687	194	2333	12.2%	0.67 [0.55, 0.81]
**Subtotal (95% CI)**		**31283**		**24617**	**99.4%**	**0.85 [0.80, 0.91]**
Total events	2161		1995			
Heterogeneity: Chi² = 11.14, df = 9 (P = 0.27); I² = 19%						
Test for overall effect: Z = 4.87 (P < 0.00001)						
**4.3.2 Vs Active Comparator**						
Jabbour 2020	2	230	3	227	0.1%	0.65 [0.11, 3.96]
Lingvay 2019	0	394	1	394	0.1%	0.33 [0.01, 8.19]
Ridderstrale 2018	8	780	8	765	0.4%	0.98 [0.37, 2.63]
Rodbard 2019	1	410	0	411	0.0%	3.01 [0.12, 74.22]
**Subtotal (95% CI)**		**1814**		**1797**	**0.6%**	**0.91 [0.41, 2.00]**
Total events	11		12			
Heterogeneity: Chi² = 1.07, df = 3 (P = 0.79); I² = 0%						
Test for overall effect: Z = 0.24 (P = 0.81)						
**Total (95% CI)**		**33097**		**26414**	**100.0%**	**0.85 [0.80, 0.91]**
Total events	2172		2007			
Heterogeneity: Chi² = 12.23, df = 13 (P = 0.51); I² = 0%						
Test for overall effect: Z = 4.88 (P < 0.00001)						
Test for subgroup differences: Chi² = 0.03, df = 1 (P = 0.87), I² = 0%						

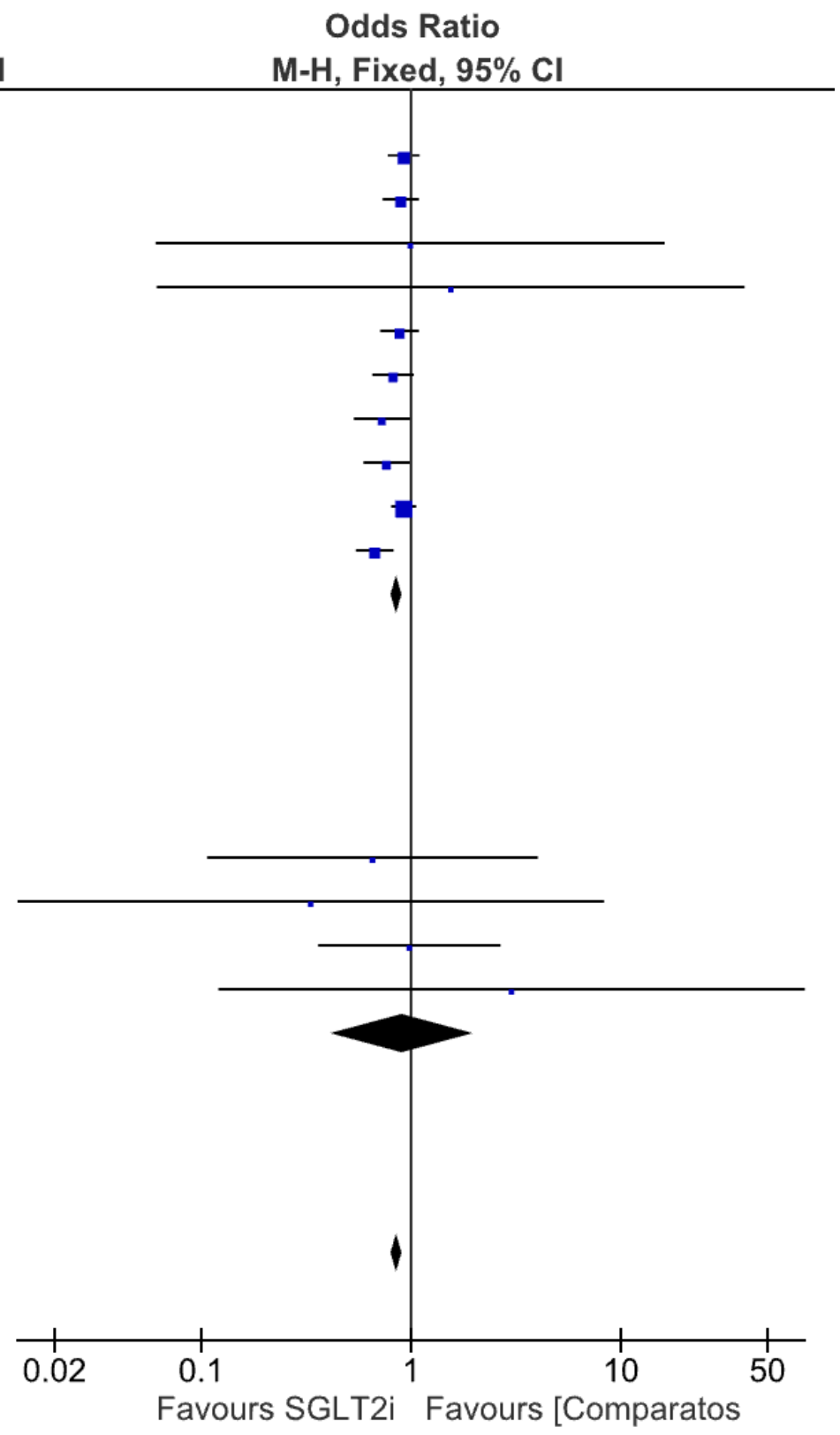

Fig. S19 Overall mortality: SGLT2i vs comparators

Study or Subgroup	GLP1-RA Events	GLP1-RA Total	Comparator Events	Comparator Total	Weight	Odds Ratio M-H, Random, 95% CI
**3.2.1 GLP1-RA vs Placebo**						
Bonaca 2025	4	396	9	396	0.6%	0.44 [0.13, 1.44]
Davies 2015	1	634	0	212	0.1%	1.01 [0.04, 24.80]
Davies 2021	2	805	1	402	0.2%	1.00 [0.09, 11.05]
Gerstein 2019	536	4949	592	4952	12.8%	0.89 [0.79, 1.01]
Holman 2017	507	7356	584	7396	12.8%	0.86 [0.76, 0.98]
Husain 2019	23	1591	45	1592	2.9%	0.50 [0.30, 0.84]
Kosiborod 2024	6	310	10	306	0.8%	0.58 [0.21, 1.63]
Marso 2016	381	4668	447	4672	11.9%	0.84 [0.73, 0.97]
Marso 2016 bis	62	1648	60	1649	4.9%	1.04 [0.72, 1.49]
Mc Guire 2025	528	4825	577	4825	12.7%	0.90 [0.80, 1.03]
Perkovic 2024	227	1767	279	1766	9.9%	0.79 [0.65, 0.95]
Pfeffer 2015	223	3034	221	3034	9.8%	1.01 [0.83, 1.23]
Pratley 2019	7	569	1	142	0.2%	1.76 [0.21, 14.39]
Zinman 2019	3	547	0	184	0.1%	2.37 [0.12, 46.14]
**Subtotal (95% CI)**		**33099**		**31528**	**79.8%**	**0.88 [0.83, 0.93]**
Total events	2510		2826			
Heterogeneity: Tau² = 0.00; Chi² = 12.24, df = 13 (P = 0.51); I² = 0%						
Test for overall effect: Z = 4.61 (P < 0.00001)						
**3.2.3 GLP1-RA vs. active comparator**						
Ahren 2017	3	818	3	407	0.3%	0.50 [0.10, 2.47]
Billings 2025	1	143	1	139	0.1%	0.97 [0.06, 15.69]
Blonde 2015	2	588	3	296	0.3%	0.33 [0.06, 2.01]
Frias 2021	1	469	12	1410	0.2%	0.25 [0.03, 1.92]
Giorgino 2015	1	545	2	262	0.2%	0.24 [0.02, 2.65]
Gouch 2015	1	414	1	413	0.1%	1.00 [0.06, 16.00]
Green 2024	27	1262	126	3785	3.9%	0.63 [0.42, 0.97]
Jabbour 2020	3	227	2	230	0.3%	1.53 [0.25, 9.22]
Lingvay 2019	1	394	0	394	0.1%	3.01 [0.12, 74.05]
Nicholls 2025	669	6579	566	6586	13.1%	1.20 [1.07, 1.35]
Rodbard 2019	0	411	1	410	0.1%	0.33 [0.01, 8.17]
Rosenstock 2019	9	1397	3	467	0.5%	1.00 [0.27, 3.72]
Tuttle 2018	9	384	6	192	0.8%	0.74 [0.26, 2.12]
Wang 2019 a	2	505	0	250	0.1%	2.49 [0.12, 52.01]
Weinstock 2015	1	606	2	177	0.2%	0.14 [0.01, 1.60]
**Subtotal (95% CI)**		**14742**		**15418**	**20.2%**	**0.80 [0.57, 1.14]**
Total events	730		728			
Heterogeneity: Tau² = 0.08; Chi² = 19.59, df = 14 (P = 0.14); I² = 29%						
Test for overall effect: Z = 1.21 (P = 0.22)						
**Total (95% CI)**		**47841**		**46946**	**100.0%**	**0.88 [0.80, 0.97]**
Total events	3240		3554			
Heterogeneity: Tau² = 0.01; Chi² = 46.98, df = 28 (P = 0.01); I² = 40%						
Test for overall effect: Z = 2.52 (P = 0.01)						
Test for subgroup differences: Chi² = 0.22, df = 1 (P = 0.64), I² = 0%						

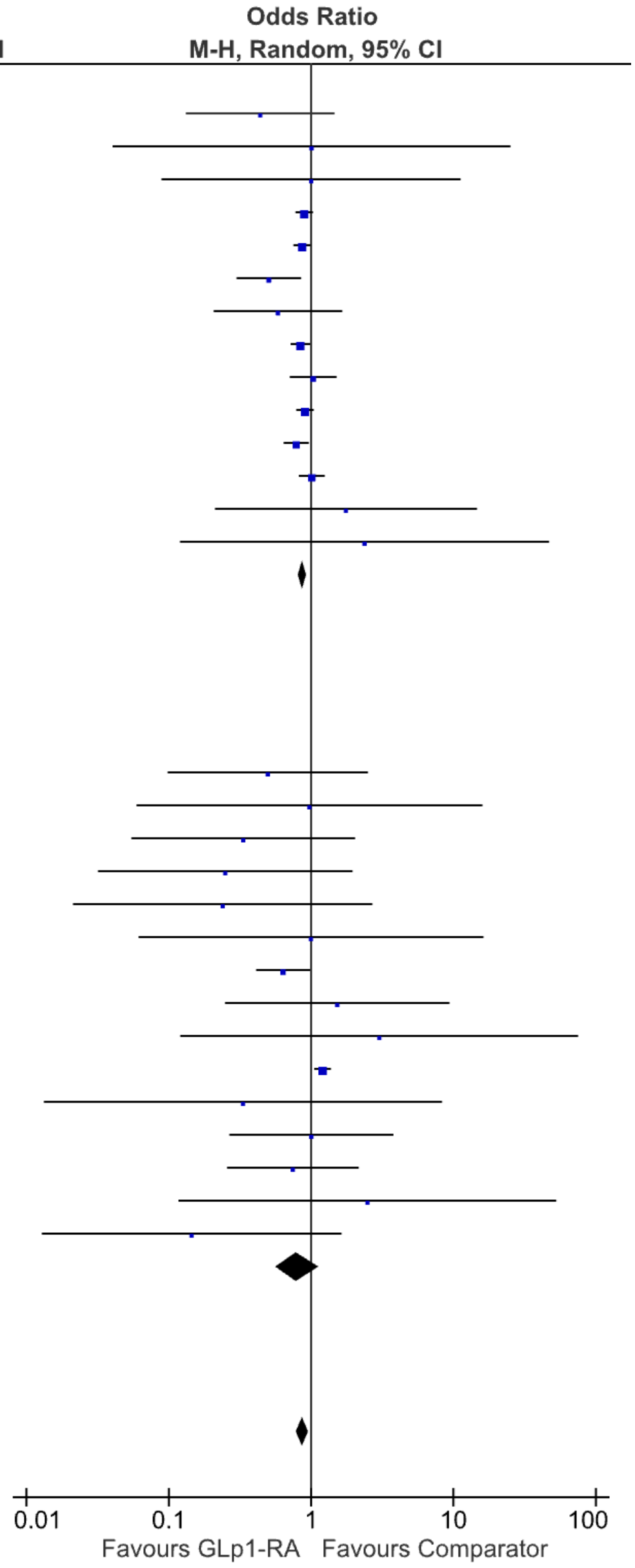

Fig. S20 Overall mortality: GLP1-RA vs comparators

Study or Subgroup	Metformin Events	Metformin Total	Control Events	Control Total	Weight	Odds Ratio M-H, Random, 95% CI
Hong 2013	7	156	14	148	13.4%	0.45 [0.18, 1.15]
Kahn 2006	31	1445	31	1441	25.2%	1.00 [0.60, 1.65]
UKPDS 34-1 1998	50	342	279	1362	31.8%	0.66 [0.48, 0.92]
UKPDS 34-2 1998	55	268	107	269	29.6%	0.39 [0.27, 0.57]
**Total (95% CI)**		**2211**		**3220**	**100.0%**	**0.60 [0.39, 0.91]**
Total events	143		431			
Heterogeneity: Tau² = 0.12; Chi² = 9.42, df = 3 (P = 0.02); I² = 68%						
Test for overall effect: Z = 2.39 (P = 0.02)						

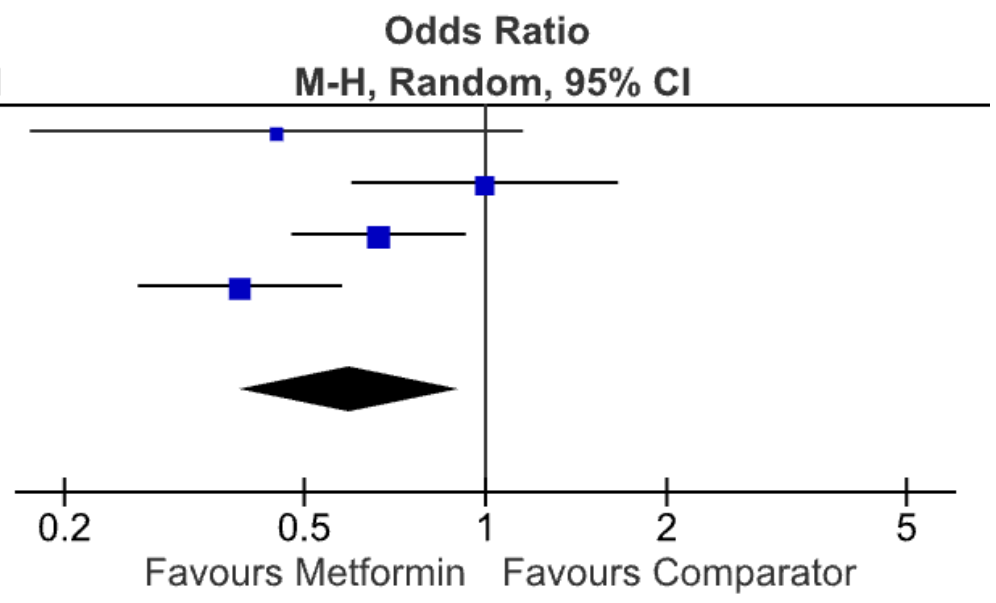

Fig.S 21 Overall mortality: metformin vs comparators

Study or Subgroup	DPP4i Events	DPP4i Total	Control Events	Control Total	Weight	Odds Ratio M-H, Fixed, 95% CI
Ahren 2014	2	302	6	408	0.2%	0.45 [0.09, 2.23]
Ahren 2017	1	407	2	818	0.1%	1.00 [0.09, 11.12]
Arjona-Ferreira 2013	1	211	5	212	0.2%	0.20 [0.02, 1.70]
Bolli 2009	3	295	2	281	0.1%	1.43 [0.24, 8.64]
Buse 2020	0	98	1	253	0.0%	0.85 [0.03, 21.15]
Cioffi 2021	0	93	1	91	0.1%	0.32 [0.01, 8.02]
Del Prato 2014	14	1665	11	874	0.6%	0.67 [0.30, 1.47]
Foley 2009	8	546	16	546	0.7%	0.49 [0.21, 1.16]
Gallwitz 2012	11	776	23	775	0.9%	0.47 [0.23, 0.97]
Green 2015	745	7332	746	7339	27.7%	1.00 [0.90, 1.11]
Green 2024	69	1268	172	3790	3.4%	1.21 [0.91, 1.61]
Henry 2014	3	231	2	693	0.0%	4.55 [0.75, 27.38]
Kaku 2019 bis	0	213	1	214	0.1%	0.33 [0.01, 8.23]
Li 2020	1	75	0	74	0.0%	3.00 [0.12, 74.84]
Matthei 2016	0	153	1	162	0.1%	0.35 [0.01, 8.67]
Matthews 2019	21	998	29	1003	1.2%	0.72 [0.41, 1.27]
Mc Murray 2018	13	128	7	126	0.3%	1.92 [0.74, 4.99]
Mita 2016	0	172	2	169	0.1%	0.19 [0.01, 4.08]
Mita 2016 bis	4	142	2	140	0.1%	2.00 [0.36, 11.10]
Nauck 2016	2	135	2	409	0.0%	3.06 [0.43, 21.94]
Oyama 2016	4	222	3	220	0.1%	1.33 [0.29, 6.00]
Rosenstock 2019	0	467	3	1397	0.1%	0.43 [0.02, 8.26]
Rosenstock 2019 bis	434	3494	420	3485	15.2%	1.04 [0.90, 1.19]
Rosenstock 2019 ter	356	3023	362	3010	13.2%	0.98 [0.84, 1.14]
Scirica 2013	613	8280	609	8212	23.4%	1.00 [0.89, 1.12]
Weinstock 2015	3	177	5	606	0.1%	2.07 [0.49, 8.76]
White 2013	315	2701	316	2679	11.6%	0.99 [0.84, 1.17]
Yki-jarvinen 2013	18	631	11	630	0.4%	1.65 [0.77, 3.53]
**Total (95% CI)**		**34235**		**38616**	**100.0%**	**1.00 [0.94, 1.06]**
Total events	2641		2760			

Heterogeneity: Chi² = 26.83, df = 27 (P = 0.47); I² = 0%
Test for overall effect: Z = 0.04 (P = 0.97)

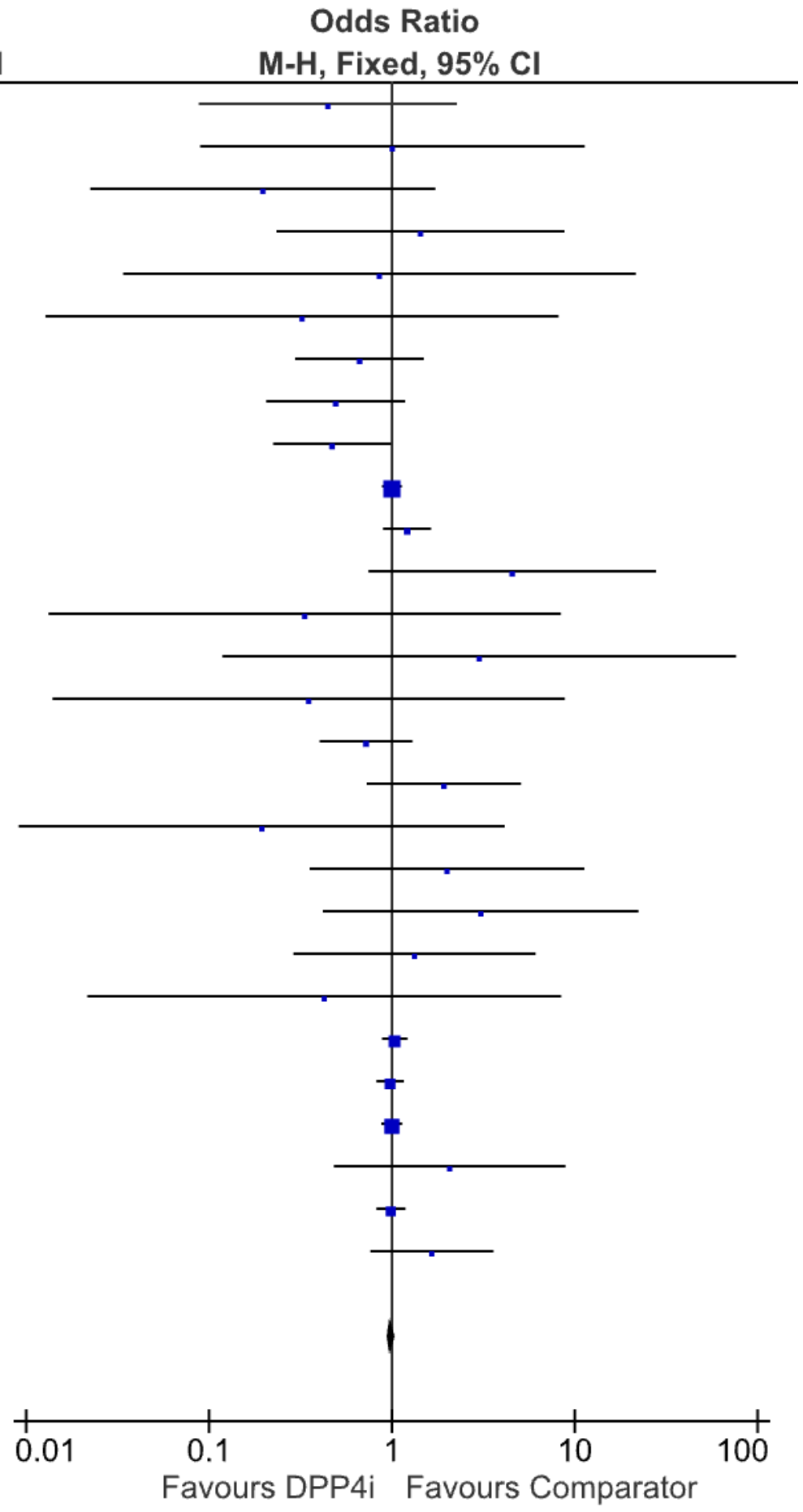

Fig. S22 Overall mortality: DPP4i vs comparators

Study or Subgroup	Insulin Events	Insulin Total	Control Events	Control Total	Weight	Odds Ratio M-H, Fixed, 95% CI
**5.3.1 Vs active comparator**						
Blonde 2015	3	296	2	588	0.1%	3.00 [0.50, 18.05]
Del Prato 2021	35	1005	25	997	2.6%	1.40 [0.83, 2.36]
Gao 2023	2	230	1	687	0.1%	6.02 [0.54, 66.67]
Giorgino 2015	2	262	1	545	0.1%	4.18 [0.38, 46.36]
Gouch 2015	1	413	1	414	0.1%	1.00 [0.06, 16.08]
Green 2024	42	1263	111	3784	5.9%	1.14 [0.79, 1.63]
Ludvik 2021	1	365	4	1079	0.2%	0.74 [0.08, 6.63]
Rosenstock 2023 bis	11	711	7	717	0.7%	1.59 [0.61, 4.14]
Tuttle 2018	6	192	9	384	0.6%	1.34 [0.47, 3.83]
UKPDS 33 1998	0	0	0	0		Not estimable
Wang 2019 a	0	250	2	505	0.2%	0.40 [0.02, 8.41]
**Subtotal (95% CI)**		**4987**		**9700**	**10.7%**	**1.30 [1.00, 1.68]**
Total events	103		163			
Heterogeneity: Chi² = 4.94, df = 9 (P = 0.84); I² = 0%						
Test for overall effect: Z = 1.96 (P = 0.05)						
**5.3.4 Vs placebo**						
Gerstein 2012	951	6264	965	6273	89.3%	0.98 [0.89, 1.09]
**Subtotal (95% CI)**		**6264**		**6273**	**89.3%**	**0.98 [0.89, 1.09]**
Total events	951		965			
Heterogeneity: Not applicable						
Test for overall effect: Z = 0.31 (P = 0.75)						
**Total (95% CI)**		**11251**		**15973**	**100.0%**	**1.02 [0.93, 1.11]**
Total events	1054		1128			
Heterogeneity: Chi² = 8.65, df = 10 (P = 0.57); I² = 0%						
Test for overall effect: Z = 0.38 (P = 0.70)						
Test for subgroup differences: Chi² = 3.80, df = 1 (P = 0.05), I² = 73.7%						

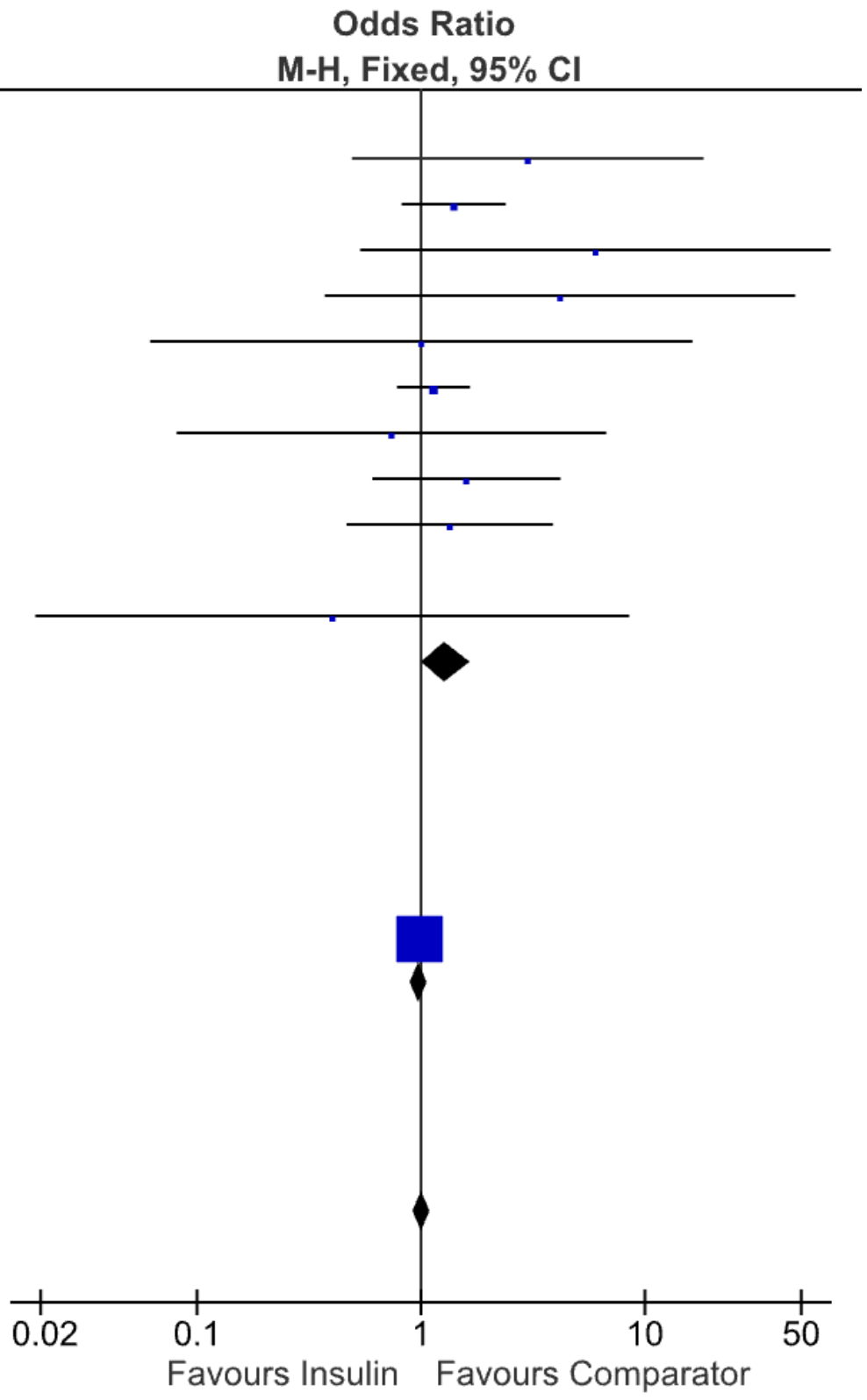

Fig. 23 Overall mortality: insulin vs comparators

Study or Subgroup	SU Events	SU Total	Control Events	Control Total	Weight	Odds Ratio M-H, Fixed, 95% CI
Ahren 2014	0	307	6	403	0.8%	0.10 [0.01, 1.77]
Arjona-Ferreira 2013	6	212	4	211	0.6%	1.51 [0.42, 5.42]
Del Prato 2014	5	874	6	1665	0.6%	1.59 [0.48, 5.23]
Foley 2009	9	546	6	546	0.9%	1.51 [0.53, 4.27]
Gallwitz 2012	4	775	4	776	0.6%	1.00 [0.25, 4.02]
Giles 2010	2	151	2	149	0.3%	0.99 [0.14, 7.10]
Green 2024	43	1254	110	3793	7.7%	1.19 [0.83, 1.70]
Hong 2013	14	148	7	156	0.9%	2.22 [0.87, 5.67]
Kahn 2006	31	1441	31	1445	4.4%	1.00 [0.61, 1.66]
Mazzone 2006	1	228	0	230	0.1%	3.04 [0.12, 75.01]
Nissen 2008	2	273	3	270	0.4%	0.66 [0.11, 3.96]
Ridderstrale 2018	8	765	8	780	1.2%	1.02 [0.38, 2.73]
Rosenstock 2019 ter	336	3010	308	3023	40.1%	1.11 [0.94, 1.30]
UKPDS 33 1998	257	1234	374	1897	34.3%	1.07 [0.90, 1.28]
Vaccaro 2017	55	1535	50	1493	7.2%	1.07 [0.73, 1.58]
**Total (95% CI)**		**12753**		**16837**	**100.0%**	**1.10 [0.99, 1.22]**
Total events	773		919			
Heterogeneity: Chi² = 6.97, df = 14 (P = 0.94); I² = 0%						
Test for overall effect: Z = 1.84 (P = 0.07)						

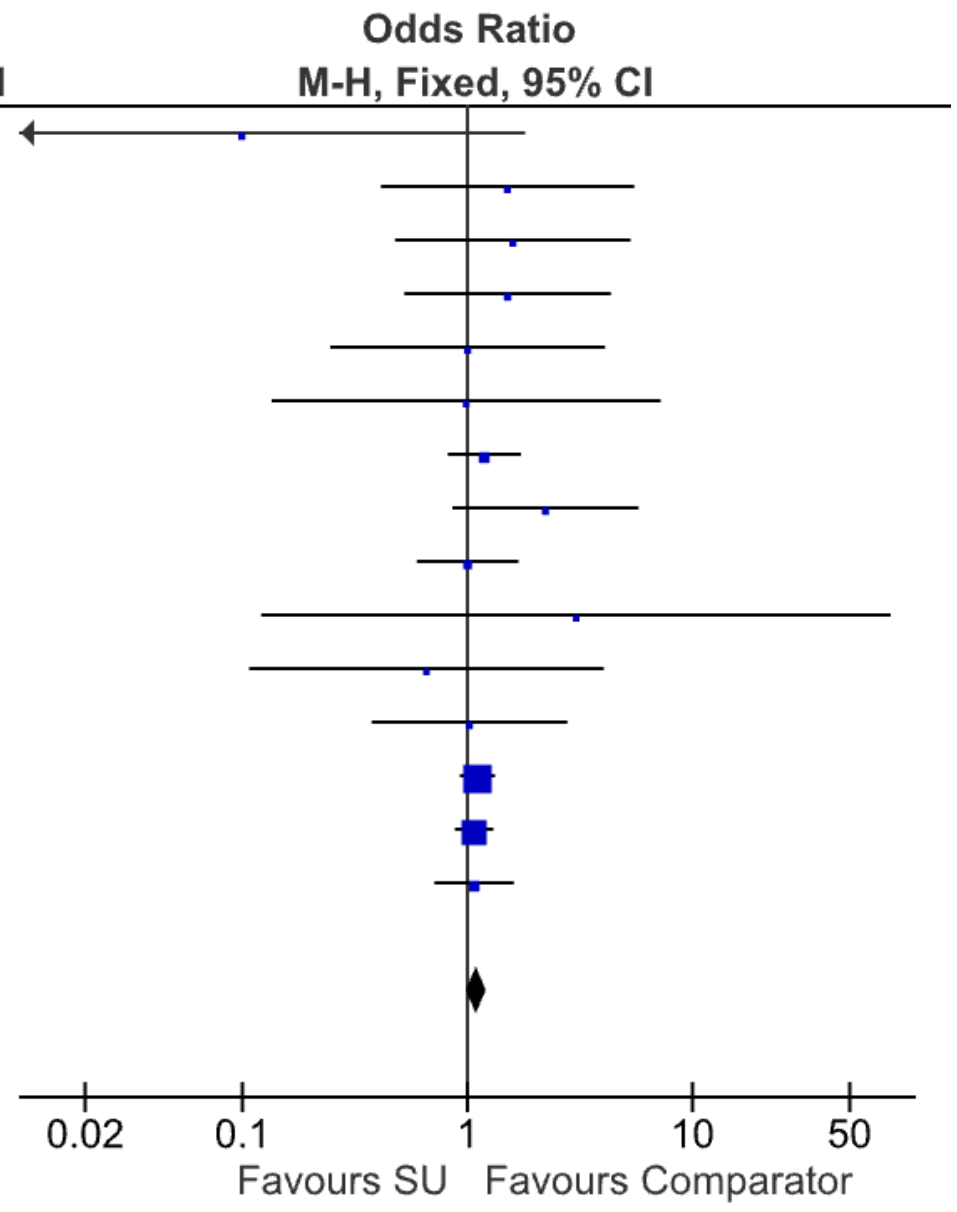

Fig. S24 Overall mortality: SU vs comparators

Figure S25: Network plot (Panel A: number of trials; panel B: node size is proportional to number of included patients**)**

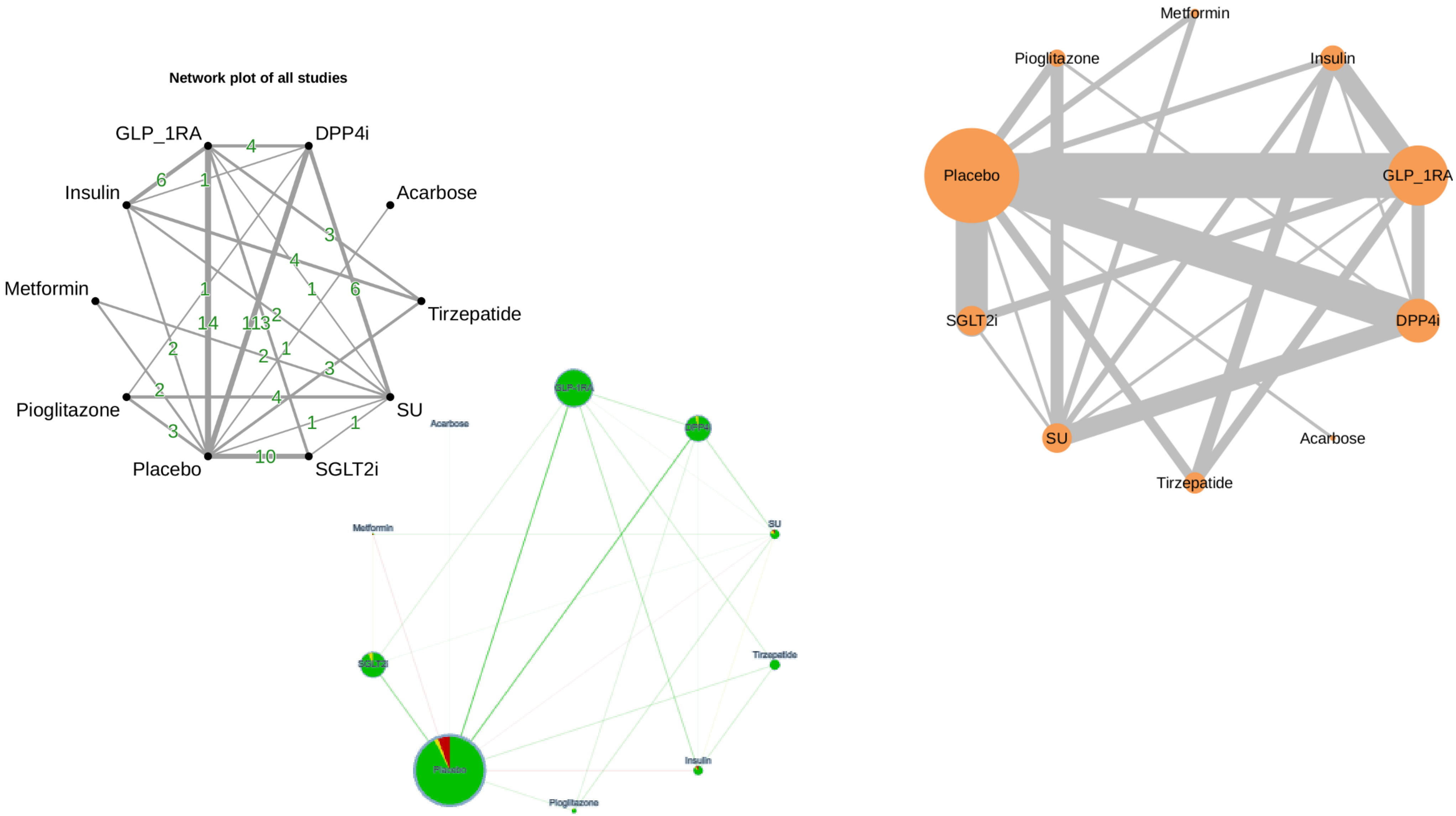

Fig. S26, Bayesian Meta-analysis, forest plot: difference in overall mortality between Diabetes drug classes and placebo.

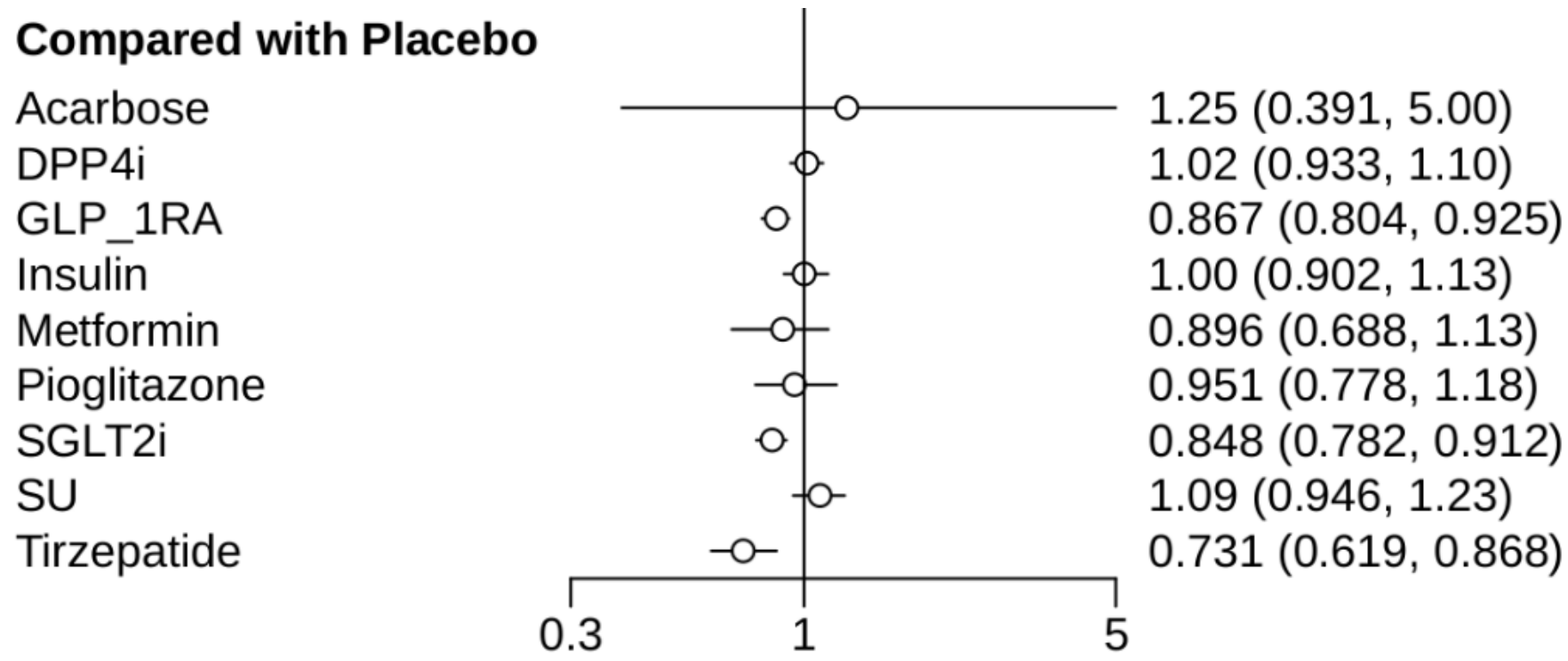

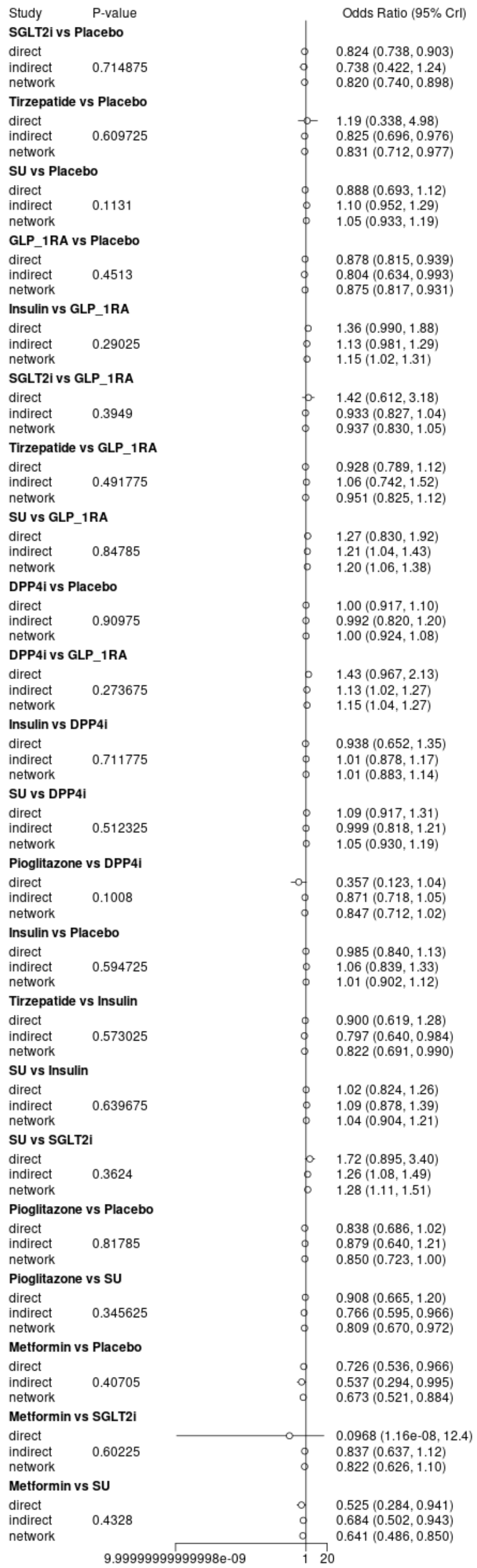

Fig. S27 Nodesplit analysis (inconsistency assessment).

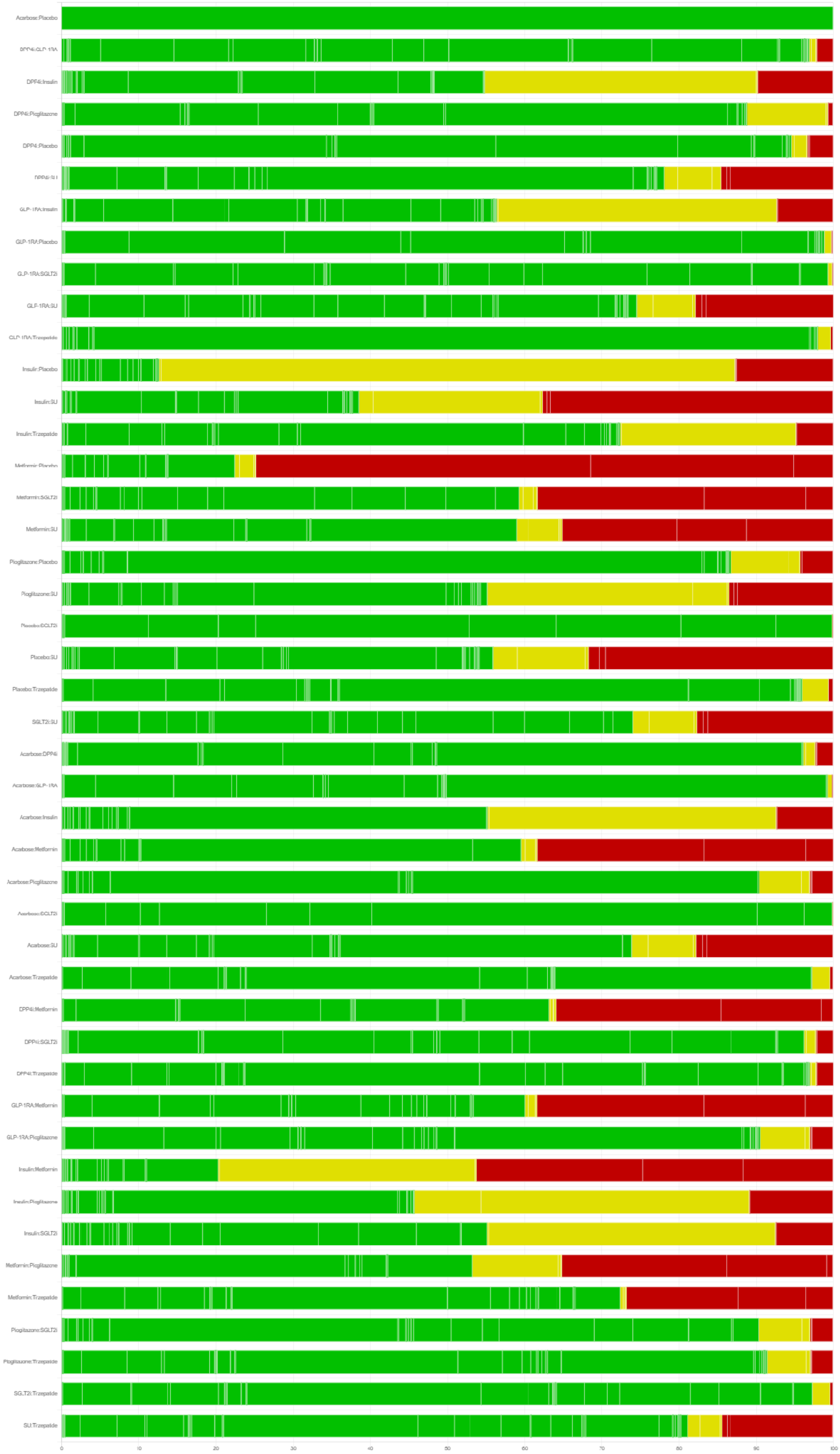

Figure S28 within study bias

Comparison	N	Within-study bias	Reporting bias	Indirectness	Imprecision	Heterogeneity	Incoherence	Confidence rating	Reason(s) for downgrading
**Acarbose vs Placebo**	**1**	No concerns	Low risk	No concerns	Major concerns	No concerns	No concerns	**Low**	Imprecision
**DPP4i vs GLP-1RA**	**5**	No concerns	Low risk	No concerns	No concerns	No concerns	No concerns	**High**	
**DPP4i vs Insulin**	**1**	Some concerns	Low risk	No concerns	No concerns	No concerns	No concerns	**Moderate**	Within-study bias
**DPP4i vs Pioglitazone**	**3**	No concerns	Low risk	No concerns	Some concerns	No concerns	No concerns	**Moderate**	Imprecision
**DPP4i vs Placebo**	**15**	No concerns	Low risk	No concerns	No concerns	No concerns	No concerns	**High**	
**DPP4i vs SU**	**6**	No concerns	Low risk	No concerns	No concerns	No concerns	No concerns	**High**	
**GLP-1RA vs Insulin**	**7**	Some concerns	Low risk	No concerns	No concerns	No concerns	No concerns	**Moderate**	Within-study bias
**GLP-1RA vs Placebo**	**16**	No concerns	Low risk	No concerns	No concerns	No concerns	No concerns	**High**	
**GLP-1RA vs SGLT2i**	**3**	No concerns	Low risk	No concerns	No concerns	No concerns	No concerns	**High**	
**GLP-1RA vs SU**	**1**	No concerns	Low risk	No concerns	No concerns	No concerns	No concerns	**High**	
**GLP-1RA vs Tirzepatide**	**4**	No concerns	Low risk	No concerns	No concerns	No concerns	No concerns	**High**	
**Insulin vs Placebo**	**2**	Some concerns	Some concerns	No concerns	No concerns	No concerns	No concerns	**Moderate**	within-study &reporting bias
**Insulin vs SU**	**2**	Some concerns	Low risk	No concerns	Some concerns	No concerns	No concerns	**Low**	within-study bias imprecision
**Insulin vs Tirzepatide**	**4**	No concerns	Low risk	No concerns	No concerns	No concerns	No concerns	**High**	
**Metformin vs Placebo**	**2**	Major concerns	Some concerns	No concerns	Some concerns	No concerns	No concerns	**Low**	Imprecision within-study bias reporting bias
**Metformin vs SGLT2i**	**1**	Some concerns	Low risk	No concerns	Major concerns	No concerns	No concerns	**Low**	within-study bias imprecision
**Metformin vs SU**	**2**	Some concerns	Some concerns	No concerns	Some concerns	No concerns	No concerns	**Low**	within-study bias reporting bias Imprecision
**Pioglitazone vs Placebo**	**3**	No concerns	Low risk	No concerns	Some concerns	No concerns	No concerns	**High**	within-study bias reporting bias Imprecision
**Pioglitazone vs SU**	**4**	Some concerns	Low risk	No concerns	Some concerns	No concerns	No concerns	**Moderate**	within-study bias imprecision
**SGLT2i vs Placebo**	**10**	No concerns	Low risk	No concerns	No concerns	No concerns	No concerns	**High**	
**SU vs Placebo**	**1**	Some concerns	Some concerns	No concerns	Some concerns	No concerns	No concerns	**Low**	within-study bias reporting bias Imprecision
**Tirzepatide vs Placebo**	**4**	No concerns	Low risk	No concerns	No concerns	No concerns	No concerns	**High**	
**SGLT2i vs SU**	**1**	No concerns	Low risk	No concerns	No concerns	No concerns	No concerns	**High**	
**Acarbose vs DPP4i**		No concerns	Low risk	No concerns	Major concerns	No concerns	No concerns	**Low**	Imprecision
**Acarbose vs GLP-1RA**		No concerns	Low risk	No concerns	Major concerns	No concerns	No concerns	**Low**	Imprecision
**Acarbose vs Insulin**		Some concerns	Low risk	No concerns	Major concerns	No concerns	No concerns	**Low**	within-study bias imprecision
**Acarbose vs Metformin**		Some concerns	Low risk	No concerns	Major concerns	No concerns	No concerns	**Low**	within-study bias imprecision
**Acarbose vs Pioglitazone**		No concerns	Low risk	No concerns	Major concerns	No concerns	No concerns	**Low**	Imprecision
**Acarbose vs SGLT2i**		No concerns	Low risk	No concerns	Major concerns	No concerns	No concerns	**Low**	Imprecision
**Acarbose vs SU**		No concerns	Low risk	No concerns	Major concerns	No concerns	No concerns	**Low**	Imprecision
**Acarbose vs Tirzepatide**		No concerns	Low risk	No concerns	Major concerns	No concerns	No concerns	**Low**	Imprecision
**DPP4i vs Metformin**		Some concerns	Low risk	No concerns	Some concerns	No concerns	No concerns	**Low**	within-study bias imprecision
**DPP4i vs SGLT2i**		No concerns	Low risk	No concerns	No concerns	No concerns	No concerns	**High**	
**DPP4i vs Tirzepatide**		No concerns	Low risk	No concerns	No concerns	No concerns	No concerns	**High**	
**GLP-1RA vs Metformin**		Some concerns	Low risk	No concerns	Major concerns	No concerns	No concerns	**Low**	Within-study bias
**GLP-1RA vs Pioglitazone**		No concerns	Low risk	No concerns	Some concerns	No concerns	No concerns	**Moderate**	Imprecision
**Insulin vs Metformin**		Some concerns	Low risk	No concerns	Some concerns	No concerns	No concerns	**Low**	within-study bias imprecision
**Insulin vs Pioglitazone**		Some concerns	Low risk	No concerns	Some concerns	No concerns	No concerns	**Low**	within-study bias imprecision
**Insulin vs SGLT2i**		Some concerns	Low risk	No concerns	No concerns	No concerns	No concerns	**High**	Within-study bias
**Metformin vs Pioglitazone**		Some concerns	Low risk	No concerns	Major concerns	No concerns	No concerns	**Low**	within-study bias imprecision
**Metformin vs Tirzepatide**		Some concerns	Low risk	No concerns	Some concerns	No concerns	No concerns	**Moderate**	within-study bias imprecision
**Pioglitazone vs SGLT2i**		No concerns	Low risk	No concerns	Some concerns	No concerns	No concerns	**Moderate**	Imprecision
**Pioglitazone vs Tirzepatide**		No concerns	Low risk	No concerns	No concerns	No concerns	No concerns	**High**	
**SGLT2i vs Tirzepatide**		No concerns	Low risk	No concerns	No concerns	No concerns	No concerns	**High**	
**SU vs Tirzepatide**		No concerns	Low risk	No concerns	No concerns	No concerns	No concerns	**High**	

Figure S29 Certainty of bias

Figure S30 Non fatal Stroke forest plot

Non fatal stroke: Indirect comparisons:

**Pioglitazone**								
1.05 [0.53; 2.10]	**Metformin**							
0.92 [0.70; 1.22]	0.87 [0.46; 1.66]	**GLP1 RA**						
0.82 [0.61; 1.10]	0.77 [0.40; 1.49]	0.89 [0.74; 1.06]	**DPP4i**					
0.81 [0.54; 1.22]	0.77 [0.38; 1.58]	0.88 [0.64; 1.23]	1.00 [0.72; 1.39]	**Insulin**				
0.81 [0.62; 1.06]	0.77 [0.41; 1.46]	0.88 [0.80; 0.97]	0.99 [0.85; 1.17]	1.00 [0.72; 1.38]	**Placebo**			
0.74 [0.53; 1.03]	0.70 [0.36; 1.36]	0.80 [0.64; 1.00]	0.90 [0.70; 1.17]	0.90 [0.62; 1.33]	0.91 [0.74; 1.11]	**SGLT2i**		
0.55 [0.15; 2.01]	0.52[0.13; 2.16]	0.60 [0.17; 2.13]	0.68 [0.19; 2.41]	0.68 [0.19; 2.40]	0.68 [0.19; 2.41]	0.75 [0.21; 2.70]	**Tirzepatide**	
0.70 [0.51; 0.96]	0.66[0.34; 1.30]	0.76 [0.60; 0.96]	0.86 [0.69; 1.06]	0.86 [0.62; 1.19]	0.86 [0.69; 1.08]	0.95 [0.70; 1.29]	1.27 [0.35; 4.53]	**SU**

Direct comparisons:

**Pioglitazone**			0.47 [0.06; 3.94]		0.81 [0.60; 1.08]		.	0.76 [0.41; 1.44]
	**Metformin**				0.77 [0.41; 1.46]			
		**GLP1 RA**	1.03 [0.57; 1.88]	0.77 [0.45; 1.32]	0.88 [0.80; 0.97]	3.05 [0.12; 75.34]	0.57 [0.06; 5.47]	1.18 [0.61; 2.31]
			**DPP4i**	0.78 [0.42; 1.45]	1.02 [0.86; 1.22]	.	.	0.85 [0.66; 1.09
				**Insulin**	0.77 [0.47; 1.25]		0.95 [0.17; 5.47]	0.86 [0.59; 1.23]
					**Placebo**	0.90 [0.73; 1.10]	0.28 [0.01; 5.46]	0.87 [0.57; 1.31]
						**SGLT2i**		
							**Tirzepatide**	
								**SU**

Non fatal MI: indirect comparisons

Figure S31 Non Fatal MI

**Tirzepatide**								
0.94 [0.74; 1.20]	**SGLT2i**							
0.94 [0.68; 1.30]	1.00 [0.75; 1.32]	**Pioglitazone**						
0.86 [0.71; 1.04]	0.92 [0.78; 1.07]	0.92 [0.71; 1.19]	**GLP1 RA**					
0.76 [0.58; 0.99]	0.81 [0.65; 1.00]	0.81 [0.61; 1.07]	0.88 [0.73; 1.07]	0.97 [0.82; 1.16]	**SU**			
0.73 [0.49; 1.10]	0.78 [0.53; 1.13]	0.78 [0.51; 1.19]	0.85 [0.59; 1.22]	0.94 [0.66; 1.33]	0.96 [0.67; 1.39]	**Metformin**		
0.76 [0.60; 0.95]	0.80 [0.68; 0.95]	0.80 [0.62; 1.05]	0.88 [0.77; 1.00]	0.97 [0.87; 1.07]	1.00 [0.84; 1.18]	1.03 [0.72; 1.48]	DPP4i	
0.68 [0.50; 0.93]	0.72 [0.55; 0.95]	0.72 [0.51; 1.02]	0.79 [0.61; 1.02]	0.87 [0.68; 1.11]	0.89 [0.70; 1.15]	0.93 [0.61; 1.41]	0.90 [0.70; 1.15]	**Insulin**

Non fatal MI: direct comparisons

**Tirzepatide**			0.87 [0.72; 1.04]	0.60 [0.10; 3.68]		0.52 [0.13; 2.03]		
	**SGLT2i**		0.67 [0.11; 4.02]	0.84 [0.74; 0.97]	0.30 [0.10; 0.92]			
		**Pioglitazone**		0.83 [0.63; 1.09]	0.82 [0.47; 1.43]			
			**GLP_1RA**	0.92 [0.84; 1.00]	0.69 [0.39; 1.23]	.	0.58 [0.35; 0.96]	0.67 [0.40; 1.12]
				**Placebo**	1.11 [0.80; 1.54]	1.02 [0.67; 1.57]	0.98 [0.88; 1.10]	0.92 [0.65; 1.29]
					**SU**	0.82 [0.45; 1.48]	1.01 [0.81; 1.26]	0.87 [0.66; 1.15]
						**Metformin**	0.99 [0.59; 1.67]	
							**DPP4i**	1.17 [0.70; 1.94]
								**Insulin**

INCLUDED STUDIES

1. Ahrén B, Johnson SL, Stewart M, et al (2014) HARMONY 3: 104-Week Randomized, Double-Blind, Placebo- and Active-Controlled Trial Assessing the Efficacy and Safety of Albiglutide Compared With Placebo, Sitagliptin, and Glimepiride in Patients With Type 2 Diabetes Taking Metformin. Diabetes Care 37(8):2141–2148. https://doi.org/10.2337/dc14-0024

2. Ahrén B, Masmiquel L, Kumar H, et al (2017) Efficacy and safety of once-weekly semaglutide versus once-daily sitagliptin as an add-on to metformin, thiazolidinediones, or both, in patients with type 2 diabetes (SUSTAIN 2): a 56-week, double-blind, phase 3a, randomised trial. Lancet Diabetes Endocrinol 5(5):341–354. https://doi.org/10.1016/S2213-8587(17)30092-X

3. Arjona Ferreira JC, Corry D, Mogensen CE, et al (2013) Efficacy and Safety of Sitagliptin in Patients With Type 2 Diabetes and ESRD Receiving Dialysis: A 54-Week Randomized Trial. Am J Kidney Dis 61(4):579–587. https://doi.org/10.1053/j.ajkd.2012.11.043

4. Billings LK, Winne L, Sharma P, Gomez-Valderas E, Chivukula KK, Kwan AYM (2025) Comparison of Dose Escalation Versus Switching to Tirzepatide Among People With Type 2 Diabetes Inadequately Controlled on Lower Doses of Dulaglutide: A Randomized Clinical Trial. Ann Intern Med 178(5):609–619. https://doi.org/10.7326/ANNALS-24-03849

5. Blonde L, Jendle J, Gross J, et al (2015) Once-weekly dulaglutide versus bedtime insulin glargine, both in combination with prandial insulin lispro, in patients with type 2 diabetes (AWARD-4): a randomised, open-label, phase 3, non-inferiority study. The Lancet 385(9982):2057–2066. https://doi.org/10.1016/S0140-6736(15)60936-9

6. Bolli G, Dotta F, Colin L, Minic B, Goodman M (2009) Comparison of vildagliptin and pioglitazone in patients with type 2 diabetes inadequately controlled with metformin. Diabetes Obes Metab 11(6):589–595. https://doi.org/10.1111/j.1463-1326.2008.01023.x

7. Bonaca MP, Catarig A-M, Houlind K, et al (2025) Semaglutide and walking capacity in people with symptomatic peripheral artery disease and type 2 diabetes (STRIDE): a phase 3b, double-blind, randomised, placebo-controlled trial. The Lancet 405(10489):1580–1593. https://doi.org/10.1016/S0140-6736(25)00509-4

8. Buse JB, Bode BW, Mertens A, et al (2020) Long-term efficacy and safety of oral semaglutide and the effect of switching from sitagliptin to oral semaglutide in patients with type 2 diabetes: a 52-week, randomized, open-label extension of the PIONEER 7 trial. BMJ Open Diabetes Res Care 8(2):e001649. https://doi.org/10.1136/bmjdrc-2020-001649

9. Cannon CP, Pratley R, Dagogo-Jack S, et al (2020) Cardiovascular Outcomes with Ertugliflozin in Type 2 Diabetes. N Engl J Med 383(15):1425–1435. https://doi.org/10.1056/NEJMoa2004967

10. Cioffi G, Giorda CB, Lucci D, et al (2021) Effects of linagliptin on left ventricular DYsfunction in patients with type 2 DiAbetes and concentric left ventricular geometry: results of the DYDA 2 trial. Eur J Prev Cardiol 28(1):8–17. https://doi.org/10.1177/2047487320939217

11. Dahl D, Onishi Y, Norwood P, et al (2022) Effect of Subcutaneous Tirzepatide vs Placebo Added to Titrated Insulin Glargine on Glycemic Control in Patients With Type 2 Diabetes: The SURPASS-5 Randomized Clinical Trial. JAMA 327(6):534. https://doi.org/10.1001/jama.2022.0078

12. Davies MJ, Bergenstal R, Bode B, et al (2015) Efficacy of Liraglutide for Weight Loss Among Patients With Type 2 Diabetes: The SCALE Diabetes Randomized Clinical Trial. JAMA 314(7):687. https://doi.org/10.1001/jama.2015.9676

13. Davies M, Færch L, Jeppesen OK, et al (2021) Semaglutide 2·4 mg once a week in adults with overweight or obesity, and type 2 diabetes (STEP 2): a randomised, double-blind, double-dummy, placebo-controlled, phase 3 trial. The Lancet 397(10278):971–984. https://doi.org/10.1016/S0140-6736(21)00213-0

14. Del Prato S, Camisasca R, Wilson C, Fleck P (2014) Durability of the efficacy and safety of alogliptin compared with glipizide in type 2 diabetes mellitus: a 2-year study. Diabetes Obes Metab 16(12):1239–1246. https://doi.org/10.1111/dom.12377

15. Del Prato S, Kahn SE, Pavo I, et al (2021) Tirzepatide versus insulin glargine in type 2 diabetes and increased cardiovascular risk (SURPASS-4): a randomised, open-label, parallel-group, multicentre, phase 3 trial. The Lancet 398(10313):1811–1824. https://doi.org/10.1016/S0140-6736(21)02188-7

16. Dormandy JA, Charbonnel B, Eckland DJ, et al (2005) Secondary prevention of macrovascular events in patients with type 2 diabetes in the PROactive Study (PROspective pioglitAzone Clinical Trial In macroVascular Events): a randomised controlled trial. The Lancet 366(9493):1279–1289. https://doi.org/10.1016/S0140-6736(05)67528-9

17. Ferrannini E, Berk A, Hantel S, et al (2013) Long-Term Safety and Efficacy of Empagliflozin, Sitagliptin, and Metformin. Diabetes Care 36(12):4015–4021. https://doi.org/10.2337/dc13-0663

18. Foley JE, Sreenan S (2009) Efficacy and Safety Comparison Between the DPP-4 Inhibitor Vildagliptin and the Sulfonylurea Gliclazide After Two Years of Monotherapy in Drug-naïve Patients with Type 2 Diabetes. Horm Metab Res 41(12):905–909. https://doi.org/10.1055/s-0029-1234042

19. Frías JP, Davies MJ, Rosenstock J, et al (2021) Tirzepatide versus Semaglutide Once Weekly in Patients with Type 2 Diabetes. N Engl J Med 385(6):503–515. https://doi.org/10.1056/NEJMoa2107519

20. Gallwitz B, Guzman J, Dotta F, et al (2012) Exenatide twice daily versus glimepiride for prevention of glycaemic deterioration in patients with type 2 diabetes with metformin failure (EUREXA): an open-label, randomised controlled trial. The Lancet 379(9833):2270–2278. https://doi.org/10.1016/S0140-6736(12)60479-6

21. Gao L, Lee BW, Chawla M, et al (2023) Tirzepatide versus insulin glargine as second-line or third-line therapy in type 2 diabetes in the Asia-Pacific region: the SURPASS-AP-Combo trial. Nat Med 29(6):1500–1510. https://doi.org/10.1038/s41591-023-02344-1

22. Garvey WT, Frias JP, Jastreboff AM, et al (2023) Tirzepatide once weekly for the treatment of obesity in people with type 2 diabetes (SURMOUNT-2): a double-blind, randomised, multicentre, placebo-controlled, phase 3 trial. The Lancet 402(10402):613–626. https://doi.org/10.1016/S0140-6736(23)01200-X

23. The ORIGIN Trial Investigators (2012) Basal Insulin and Cardiovascular and Other Outcomes in Dysglycemia. N Engl J Med 367(4):319–328. https://doi.org/10.1056/NEJMoa1203858

24. Gerstein HC, Colhoun HM, Dagenais GR, et al (2019) Dulaglutide and cardiovascular outcomes in type 2 diabetes (REWIND): a double-blind, randomised placebo-controlled trial. The Lancet 394(10193):121–130. https://doi.org/10.1016/S0140-6736(19)31149-3

25. Giles TD, Elkayam U, Bhattacharya M, Perez A, Miller AB (2010) Comparison of Pioglitazone vs Glyburide in Early Heart Failure: Insights From a Randomized Controlled Study of Patients With Type 2 Diabetes and Mild Cardiac Disease. Congest Heart Fail 16(3):111–117. https://doi.org/10.1111/j.1751-7133.2010.00154.x

26. Giorgino F, Benroubi M, Sun J-H, Zimmermann AG, Pechtner V (2015) Efficacy and Safety of Once-Weekly Dulaglutide Versus Insulin Glargine in Patients With Type 2 Diabetes on Metformin and Glimepiride (AWARD-2). Diabetes Care 38(12):2241–2249. https://doi.org/10.2337/dc14-1625

27. Gough SCL, Bode BW, Woo VC, et al (2015) One-year efficacy and safety of a fixed combination of insulin degludec and liraglutide in patients with type 2 diabetes: results of a 26-week extension to a 26-week main trial. Diabetes Obes Metab 17(10):965–973. https://doi.org/10.1111/dom.12498

28. Green JB, Bethel MA, Armstrong PW, et al (2015) Effect of Sitagliptin on Cardiovascular Outcomes in Type 2 Diabetes. N Engl J Med 373(3):232–242. https://doi.org/10.1056/NEJMoa1501352

29. Green JB, Everett BM, Ghosh A, et al (2024) Cardiovascular Outcomes in GRADE (Glycemia Reduction Approaches in Type 2 Diabetes: A Comparative Effectiveness Study). Circulation 149(13):993–1003. https://doi.org/10.1161/CIRCULATIONAHA.123.066604

30. Guo L, Dong X, Ma J, et al (2025) Efficacy and safety of tirzepatide added to basal insulin in patients with type 2 diabetes in China (SURPASS-CN-INS): a double-blind, multicentre, randomised, placebo-controlled, phase 3 trial. Lancet Diabetes Endocrinol 13(12):1015–1029. https://doi.org/10.1016/S2213-8587(25)00248-7

31. Henry RR, Staels B, Fonseca VA, et al (2014) Efficacy and safety of initial combination treatment with sitagliptin and pioglitazone—a factorial study. Diabetes Obes Metab 16(3):223–230. https://doi.org/10.1111/dom.12194

32. Holman RR, Cull CA, Turner RC (1999) A randomized double-blind trial of acarbose in type 2 diabetes shows improved glycemic control over 3 years (U.K. Prospective Diabetes Study 44). Diabetes Care 22(6):960–964. https://doi.org/10.2337/diacare.22.6.960

33. Holman RR, Bethel MA, Mentz RJ, et al (2017) Effects of Once-Weekly Exenatide on Cardiovascular Outcomes in Type 2 Diabetes. N Engl J Med 377(13):1228–1239. https://doi.org/10.1056/NEJMoa1612917

34. Home PD, Shamanna P, Stewart M, et al (2015) Efficacy and tolerability of albiglutide versus placebo or pioglitazone over 1 year in people with type 2 diabetes currently taking metformin and glimepiride: HARMONY 5. Diabetes Obes Metab 17(2):179–187. https://doi.org/10.1111/dom.12414

35. Hong J, Zhang Y, Lai S, et al (2013) Effects of Metformin Versus Glipizide on Cardiovascular Outcomes in Patients With Type 2 Diabetes and Coronary Artery Disease. Diabetes Care 36(5):1304–1311. https://doi.org/10.2337/dc12-0719

36. Husain M, Birkenfeld AL, Donsmark M, et al (2019) Oral Semaglutide and Cardiovascular Outcomes in Patients with Type 2 Diabetes. N Engl J Med 381(9):841–851. https://doi.org/10.1056/NEJMoa1901118

37. Inagaki N, Takeuchi M, Oura T, Imaoka T, Seino Y (2022) Efficacy and safety of tirzepatide monotherapy compared with dulaglutide in Japanese patients with type 2 diabetes (SURPASS J-mono): a double-blind, multicentre, randomised, phase 3 trial. Lancet Diabetes Endocrinol 10(9):623–633. https://doi.org/10.1016/S2213-8587(22)00188-7

38. Inzucchi SE, Claggett BL, Vaduganathan M, et al (2022) Efficacy and safety of dapagliflozin in patients with heart failure with mildly reduced or preserved ejection fraction by baseline glycaemic status (DELIVER): a subgroup analysis from an international, multicentre, double-blind, randomised, placebo-controlled trial. Lancet Diabetes Endocrinol 10(12):869–881. https://doi.org/10.1016/S2213-8587(22)00308-4

39. Jabbour SA, Frías JP, Ahmed A, et al (2020) Efficacy and Safety Over 2 Years of Exenatide Plus Dapagliflozin in the DURATION-8 Study: A Multicenter, Double-Blind, Phase 3, Randomized Controlled Trial. Diabetes Care 43(10):2528–2536. https://doi.org/10.2337/dc19-1350

40. Kahn SE, Haffner SM, Heise MA, et al (2006) Glycemic Durability of Rosiglitazone, Metformin, or Glyburide Monotherapy. N Engl J Med 355(23):2427–2443. https://doi.org/10.1056/NEJMoa066224

41. Kaku K, Araki E, Tanizawa Y, et al (2019) Superior efficacy with a fixed-ratio combination of insulin degludec and liraglutide (IDegLira) compared with insulin degludec and liraglutide in insulin-naïve Japanese patients with type 2 diabetes in a phase 3, open-label, randomized trial. Diabetes Obes Metab 21(12):2674–2683. https://doi.org/10.1111/dom.13856

42. Kawamori R, Haneda M, Suzaki K, et al (2018) Empagliflozin as add-on to linagliptin in a fixed-dose combination in Japanese patients with type 2 diabetes: Glycaemic efficacy and safety profile in a 52-week, randomized, placebo-controlled trial. Diabetes Obes Metab 20(9):2200–2209. https://doi.org/10.1111/dom.13352

43. Kosiborod MN, Petrie MC, Borlaug BA, et al (2024) Semaglutide in Patients with Obesity-Related Heart Failure and Type 2 Diabetes. N Engl J Med 390(15):1394–1407. https://doi.org/10.1056/NEJMoa2313917

44. Li B, Luo Y-R, Tian F, et al (2020) Sitagliptin attenuates the progression of coronary atherosclerosis in patients with coronary disease and type 2 diabetes. Atherosclerosis 300:10–18. https://doi.org/10.1016/j.atherosclerosis.2020.03.015

45. Lingvay I, Catarig A-M, Frias JP, et al (2019) Efficacy and safety of once-weekly semaglutide versus daily canagliflozin as add-on to metformin in patients with type 2 diabetes (SUSTAIN 8): a double-blind, phase 3b, randomised controlled trial. Lancet Diabetes Endocrinol 7(11):834–844. https://doi.org/10.1016/S2213-8587(19)30311-0

46. Ludvik B, Giorgino F, Jódar E, et al (2021) Once-weekly tirzepatide versus once-daily insulin degludec as add-on to metformin with or without SGLT2 inhibitors in patients with type 2 diabetes (SURPASS-3): a randomised, open-label, parallel-group, phase 3 trial. The Lancet 398(10300):583–598. https://doi.org/10.1016/S0140-6736(21)01443-4

47. Marso SP, Daniels GH, Brown-Frandsen K, et al (2016) Liraglutide and Cardiovascular Outcomes in Type 2 Diabetes. N Engl J Med 375(4):311–322. https://doi.org/10.1056/NEJMoa1603827

48. Marso SP, Bain SC, Consoli A, et al (2016) Semaglutide and Cardiovascular Outcomes in Patients with Type 2 Diabetes. N Engl J Med 375(19):1834–1844. https://doi.org/10.1056/NEJMoa1607141

49. Marso SP, McGuire DK, Zinman B, et al (2017) Efficacy and Safety of Degludec versus Glargine in Type 2 Diabetes. N Engl J Med 377(8):723–732. https://doi.org/10.1056/NEJMoa1615692

50. Matthaei S, Catrinoiu D, Celiński A, et al (2015) Randomized, Double-Blind Trial of Triple Therapy With Saxagliptin Add-on to Dapagliflozin Plus Metformin in Patients With Type 2 Diabetes. Diabetes Care 38(11):2018–2024. https://doi.org/10.2337/dc15-0811

51. Matthews DR, Paldánius PM, Proot P, Chiang Y, Stumvoll M, Del Prato S (2019) Glycaemic durability of an early combination therapy with vildagliptin and metformin versus sequential metformin monotherapy in newly diagnosed type 2 diabetes (VERIFY): a 5-year, multicentre, randomised, double-blind trial. The Lancet 394(10208):1519–1529. https://doi.org/10.1016/S0140-6736(19)32131-2

52. Mazzone T, Meyer PM, Feinstein SB, et al (2006) Effect of Pioglitazone Compared With Glimepiride on Carotid Intima-Media Thickness in Type 2 Diabetes: A Randomized Trial. JAMA 296(21):2572. https://doi.org/10.1001/jama.296.21.joc60158

53. McGill JB, Sloan L, Newman J, et al (2013) Long-Term Efficacy and Safety of Linagliptin in Patients With Type 2 Diabetes and Severe Renal Impairment. Diabetes Care 36(2):237–244. https://doi.org/10.2337/dc12-0706

54. McGuire DK, Marx N, Mulvagh SL, et al (2025) Oral Semaglutide and Cardiovascular Outcomes in High-Risk Type 2 Diabetes. N Engl J Med 392(20):2001–2012. https://doi.org/10.1056/NEJMoa2501006

55. McMurray JJV, Ponikowski P, Bolli GB, et al (2018) Effects of Vildagliptin on Ventricular Function in Patients With Type 2 Diabetes Mellitus and Heart Failure. JACC Heart Fail 6(1):8–17. https://doi.org/10.1016/j.jchf.2017.08.004

56. Mita T, Katakami N, Yoshii H, et al (2016) Alogliptin, a Dipeptidyl Peptidase 4 Inhibitor, Prevents the Progression of Carotid Atherosclerosis in Patients With Type 2 Diabetes: The Study of Preventive Effects of Alogliptin on Diabetic Atherosclerosis (SPEAD-A). Diabetes Care 39(1):139–148. https://doi.org/10.2337/dc15-0781

57. Mita T, Katakami N, Shiraiwa T, et al (2016) Sitagliptin Attenuates the Progression of Carotid Intima-Media Thickening in Insulin-Treated Patients With Type 2 Diabetes: The Sitagliptin Preventive Study of Intima-Media Thickness Evaluation (SPIKE). Diabetes Care 39(3):455–464. https://doi.org/10.2337/dc15-2145

58. Miyagawa J, Odawara M, Takamura T, Iwamoto N, Takita Y, Imaoka T (2015) Once-weekly glucagon-like peptide-1 receptor agonist dulaglutide is non-inferior to once-daily liraglutide and superior to placebo in J apanese patients with type 2 diabetes: a 26-week randomized phase III study. Diabetes Obes Metab 17(10):974–983. https://doi.org/10.1111/dom.12534

59. Nauck MA, Di Domenico M, Patel S, Kobe M, Toorawa R, Woerle H-J (2016) Linagliptin and pioglitazone combination therapy versus monotherapy with linagliptin or pioglitazone: A randomised, double-blind, parallel-group, multinational clinical trial. Diab Vasc Dis Res 13(4):286–298. https://doi.org/10.1177/1479164116639229

60. Neal B, Perkovic V, Mahaffey KW, et al (2017) Canagliflozin and Cardiovascular and Renal Events in Type 2 Diabetes. N Engl J Med 377(7):644–657. https://doi.org/10.1056/NEJMoa1611925

61. Nicholls SJ, Pavo I, Bhatt DL, et al (2025) Cardiovascular Outcomes with Tirzepatide versus Dulaglutide in Type 2 Diabetes. N Engl J Med 393(24):2409–2420. https://doi.org/10.1056/NEJMoa2505928

62. Nissen SE, Nicholls SJ, Wolski K, et al (2008) Comparison of Pioglitazone vs Glimepiride on Progression of Coronary Atherosclerosis in Patients With Type 2 Diabetes: The PERISCOPE Randomized Controlled Trial. JAMA 299(13):1561. https://doi.org/10.1001/jama.299.13.1561

63. Oyama J, Murohara T, Kitakaze M, et al (2016) The Effect of Sitagliptin on Carotid Artery Atherosclerosis in Type 2 Diabetes: The PROLOGUE Randomized Controlled Trial. PLOS Med 13(6):e1002051. https://doi.org/10.1371/journal.pmed.1002051

64. Perkovic V, Jardine MJ, Neal B, et al (2019) Canagliflozin and Renal Outcomes in Type 2 Diabetes and Nephropathy. N Engl J Med 380(24):2295–2306. https://doi.org/10.1056/NEJMoa1811744

65. Perkovic V, Tuttle KR, Rossing P, et al (2024) Effects of Semaglutide on Chronic Kidney Disease in Patients with Type 2 Diabetes. N Engl J Med 391(2):109–121. https://doi.org/10.1056/NEJMoa2403347

66. Persson F, Rossing P, Vart P, et al (2021) Efficacy and Safety of Dapagliflozin by Baseline Glycemic Status: A Prespecified Analysis From the DAPA-CKD Trial. Diabetes Care 44(8):1894–1897. https://doi.org/10.2337/dc21-0300

67. Petrie MC, Verma S, Docherty KF, et al (2020) Effect of Dapagliflozin on Worsening Heart Failure and Cardiovascular Death in Patients With Heart Failure With and Without Diabetes. JAMA 323(14):1353. https://doi.org/10.1001/jama.2020.1906

68. Pfeffer MA, Claggett B, Diaz R, et al (2015) Lixisenatide in Patients with Type 2 Diabetes and Acute Coronary Syndrome. N Engl J Med 373(23):2247–2257. https://doi.org/10.1056/NEJMoa1509225

69. Pratley R, Amod A, Hoff ST, et al (2019) Oral semaglutide versus subcutaneous liraglutide and placebo in type 2 diabetes (PIONEER 4): a randomised, double-blind, phase 3a trial. The Lancet 394(10192):39–50. https://doi.org/10.1016/S0140-6736(19)31271-1

70. Ridderstråle M, Rosenstock J, Andersen KR, Woerle HJ, Salsali A, on behalf of the EMPA-REG H2H-SU trial investigators (2018) Empagliflozin compared with glimepiride in metformin-treated patients with type 2 diabetes: 208-week data from a masked randomized controlled trial. Diabetes Obes Metab 20(12):2768–2777. https://doi.org/10.1111/dom.13457

71. Rodbard HW, Rosenstock J, Canani LH, et al (2019) Oral Semaglutide Versus Empagliflozin in Patients With Type 2 Diabetes Uncontrolled on Metformin: The PIONEER 2 Trial. Diabetes Care 42(12):2272–2281. https://doi.org/10.2337/dc19-0883

72. Rosenstock J, Perkovic V, Johansen OE, et al (2019) Effect of Linagliptin vs Placebo on Major Cardiovascular Events in Adults With Type 2 Diabetes and High Cardiovascular and Renal Risk: The CARMELINA Randomized Clinical Trial. JAMA 321(1):69. https://doi.org/10.1001/jama.2018.18269

73. Rosenstock J, Allison D, Birkenfeld AL, et al (2019) Effect of Additional Oral Semaglutide vs Sitagliptin on Glycated Hemoglobin in Adults With Type 2 Diabetes Uncontrolled With Metformin Alone or With Sulfonylurea: The PIONEER 3 Randomized Clinical Trial. JAMA 321(15):1466. https://doi.org/10.1001/jama.2019.2942

74. Rosenstock J, Kahn SE, Johansen OE, et al (2019) Effect of Linagliptin vs Glimepiride on Major Adverse Cardiovascular Outcomes in Patients With Type 2 Diabetes: The CAROLINA Randomized Clinical Trial. JAMA 322(12):1155. https://doi.org/10.1001/jama.2019.13772

75. Rosenstock J, Wysham C, Frías JP, et al (2021) Efficacy and safety of a novel dual GIP and GLP-1 receptor agonist tirzepatide in patients with type 2 diabetes (SURPASS-1): a double-blind, randomised, phase 3 trial. The Lancet 398(10295):143–155. https://doi.org/10.1016/S0140-6736(21)01324-6

76. Rosenstock J, Frías JP, Rodbard HW, et al (2023) Tirzepatide vs Insulin Lispro Added to Basal Insulin in Type 2 Diabetes: The SURPASS-6 Randomized Clinical Trial. JAMA 330(17):1631. https://doi.org/10.1001/jama.2023.20294

77. Scirica BM, Bhatt DL, Braunwald E, et al (2013) Saxagliptin and Cardiovascular Outcomes in Patients with Type 2 Diabetes Mellitus. N Engl J Med 369(14):1317–1326. https://doi.org/10.1056/NEJMoa1307684

78. Tuttle KR, Lakshmanan MC, Rayner B, et al (2018) Dulaglutide versus insulin glargine in patients with type 2 diabetes and moderate-to-severe chronic kidney disease (AWARD-7): a multicentre, open-label, randomised trial. Lancet Diabetes Endocrinol 6(8):605–617. https://doi.org/10.1016/S2213-8587(18)30104-9

79. (1998) Intensive blood-glucose control with sulphonylureas or insulin compared with conventional treatment and risk of complications in patients with type 2 diabetes (UKPDS 33). UK Prospective Diabetes Study (UKPDS) Group. Lancet 352(9131):837–853

80. (1998) Effect of intensive blood-glucose control with metformin on complications in overweight patients with type 2 diabetes (UKPDS 34). The Lancet 352(9131):854–865. https://doi.org/10.1016/S0140-6736(98)07037-8

81. Vaccaro O, Masulli M, Nicolucci A, et al (2017) Effects on the incidence of cardiovascular events of the addition of pioglitazone versus sulfonylureas in patients with type 2 diabetes inadequately controlled with metformin (TOSCA.IT): a randomised, multicentre trial. Lancet Diabetes Endocrinol 5(11):887–897. https://doi.org/10.1016/S2213-8587(17)30317-0

82. Wang W, Nevárez L, Filippova E, et al (2019) Efficacy and safety of once-weekly dulaglutide versus insulin glargine in mainly Asian patients with type 2 diabetes mellitus on metformin and/or a sulphonylurea: A 52-week open-label, randomized phase III trial. Diabetes Obes Metab 21(2):234–243. https://doi.org/10.1111/dom.13506

83. Weinstock RS, Guerci B, Umpierrez G, Nauck MA, Skrivanek Z, Milicevic Z (2015) Safety and efficacy of once-weekly dulaglutide versus sitagliptin after 2 years in metformin-treated patients with type 2 diabetes (AWARD -5): a randomized, phase III study. Diabetes Obes Metab 17(9):849–858. https://doi.org/10.1111/dom.12479

84. White WB, Cannon CP, Heller SR, et al (2013) Alogliptin after Acute Coronary Syndrome in Patients with Type 2 Diabetes. N Engl J Med 369(14):1327–1335. https://doi.org/10.1056/NEJMoa1305889

85. Wiviott SD, Raz I, Bonaca MP, et al (2019) Dapagliflozin and Cardiovascular Outcomes in Type 2 Diabetes. N Engl J Med 380(4):347–357. https://doi.org/10.1056/NEJMoa1812389

86. Yabe D, Nakamura J, Kaneto H, et al (2020) Safety and efficacy of oral semaglutide versus dulaglutide in Japanese patients with type 2 diabetes (PIONEER 10): an open-label, randomised, active-controlled, phase 3a trial. Lancet Diabetes Endocrinol 8(5):392–406. https://doi.org/10.1016/S2213-8587(20)30074-7

87. Yamada Y, Katagiri H, Hamamoto Y, et al (2020) Dose-response, efficacy, and safety of oral semaglutide monotherapy in Japanese patients with type 2 diabetes (PIONEER 9): a 52-week, phase 2/3a, randomised, controlled trial. Lancet Diabetes Endocrinol 8(5):377–391. https://doi.org/10.1016/S2213-8587(20)30075-9

88. Yki-Järvinen H, Rosenstock J, Durán-Garcia S, et al (2013) Effects of Adding Linagliptin to Basal Insulin Regimen for Inadequately Controlled Type 2 Diabetes. Diabetes Care 36(12):3875–3881. https://doi.org/10.2337/dc12-2718

89. Yoshii H, Onuma T, Yamazaki T, et al (2014) Effects of Pioglitazone on Macrovascular Events in Patients with Type 2 Diabetes Mellitus at High Risk of Stroke: The PROFIT-J Study. J Atheroscler Thromb. https://doi.org/10.5551/jat.21626

90. Zinman B, Wanner C, Lachin JM, et al (2015) Empagliflozin, Cardiovascular Outcomes, and Mortality in Type 2 Diabetes. N Engl J Med 373(22):2117–2128. https://doi.org/10.1056/NEJMoa1504720

91. Zinman B, Aroda VR, Buse JB, et al (2019) Efficacy, Safety, and Tolerability of Oral Semaglutide Versus Placebo Added to Insulin With or Without Metformin in Patients With Type 2 Diabetes: The PIONEER 8 Trial. Diabetes Care 42(12):2262–2271. https://doi.org/10.2337/dc19-0898
